# Linking leadership development programs for physicians with organization-level outcomes: a realist review

**DOI:** 10.1186/s12913-023-09811-y

**Published:** 2023-07-21

**Authors:** Maarten Debets, Iris Jansen, Kiki Lombarts, Wietske Kuijer-Siebelink, Karen Kruijthof, Yvonne Steinert, Joost Daams, Milou Silkens

**Affiliations:** 1 Amsterdam UMC, Medical Psychology, Univ of Amsterdam, Amsterdam Public Health, AMC, Meibergdreef 9, 1105AZ Amsterdam, Netherlands; 2 School of Education, Research On Responsive Vocational and Professional Education, HAN University of Applied Sciences, Nijmegen, Netherlands; 3 Research On Learning and Education, Radboud University Medical Centre, Radboudumc Health Academy, Nijmegen, Netherlands; 4 Amsterdam UMC, Vrije Universiteit Amsterdam, Amsterdam Public Health, De Boelelaan 1117, Amsterdam, Netherlands; 5 Faculty of Medicine and Health Sciences, Institute of Health Sciences Education, McGill University, Montreal, Canada; 6 Medical Library, Amsterdam University Medical Centers, Amsterdam, Noord-Holland Netherlands; 7 Department of Health Services Research & Management, City University of London, London, UK; 8 Erasmus School of Health Policy & Management, Erasmus University, Rotterdam, Netherlands

**Keywords:** Leadership Development Programs (LDPs), Leadership, Physicians, Realist review, Middle-range program theory, Organization-level outcomes, Quality Improvement

## Abstract

**Background:**

Hospitals invest in Leadership Development Programs (LDPs) for physicians, assuming they benefit the organization’s performance. Researchers have listed the advantages of LDPs, but knowledge of how and why organization-level outcomes are achieved is missing.

**Objective:**

To investigate how, why and under which circumstances LDPs for physicians can impact organization-level outcomes.

**Methods:**

We conducted a realist review, following the RAMESES guidelines. Scientific articles and grey literature published between January 2010 and March 2021 evaluating a leadership intervention for physicians in the hospital setting were considered for inclusion. The following databases were searched: Medline, PsycInfo, ERIC, Web of Science, and Academic Search Premier. Based on the included documents, we developed a LDP middle-range program theory (MRPT) consisting of Context-Mechanism-Outcome configurations (CMOs) describing how specific contexts (C) trigger certain mechanisms (M) to generate organization-level outcomes (O).

**Results:**

In total, 3904 titles and abstracts and, subsequently, 100 full-text documents were inspected; 38 documents with LDPs from multiple countries informed our MRPT. The MRPT includes five CMOs that describe how LDPs can impact the organization-level outcomes categories ‘culture’, ‘quality improvement’, and ‘the leadership pipeline’: 'Acquiring self-insight and people skills (CMO1)', 'Intentionally building professional networks (CMO2)', 'Supporting quality improvement projects (CMO3)', 'Tailored LDP content prepares physicians (CMO4)', and 'Valuing physician leaders and organizational commitment (CMO5)'. Culture was the outcome of CMO1 and CMO2, quality improvement of CMO2 and CMO3, and the leadership pipeline of CMO2, CMO4, and CMO5. These CMOs operated within an overarching context, the leadership ecosystem, that determined realizing and sustaining organization-level outcomes.

**Conclusions:**

LDPs benefit organization-level outcomes through multiple mechanisms. Creating the contexts to trigger these mechanisms depends on the resources invested in LDPs and adequately supporting physicians. LDP providers can use the presented MRPT to guide the development of LDPs when aiming for specific organization-level outcomes.

**Supplementary Information:**

The online version contains supplementary material available at 10.1186/s12913-023-09811-y.

## Introduction

Hospitals are offering leadership development programs (LDPs) to physicians to ensure the delivery of high-quality, accessible, and affordable patient care [1–7]. For example, 65% of academic health centers in the United States provide formal LDPs [1]. Evidence shows that these LPDs can benefit individual-level outcomes (e.g., enhanced leadership knowledge), team-level outcomes (e.g., better teamwork), and organization-level outcomes (e.g., less complications) [2, 4–10]. At the same time, researchers have focused less on describing *how* and *why* LDPs produce these outcomes. While some studies are instrumental in explaining the links between LPD components and individual-level and team-level outcomes [11, 12], knowledge of how and why LPDs achieve organization-level outcomes is limited [2, 4, 5]. This lack of knowledge may exist because it is challenging to systematically investigate how LDPs produce organization-level outcomes, due to the heterogeneity of LDPs and the organizational contexts in which they operate [5]. Realist reviews can account for this complexity as they aim to explain how and why interventions work in particular contexts to generate outcomes [13].

This realist review builds on previous research by investigating how and why LPDs impact organization-level outcomes, which we, based on studying the literature on LDPs, define as outcomes that reflect changes in culture, quality improvement in patient care or organizational processes, and the leadership pipeline at the organizational level. The leadership pipeline refers to the availability of a pool of well-prepared leaders, i.e., the organization's leadership succession bench. Motivating physicians to lead and realize organization-level outcomes is important, as they have the medical expertise to identify quality improvement opportunities, access to scarce healthcare resources, and possible positions to persuade other healthcare professionals to adjust their way of working [3, 14, 15]. The competency-based framework, CanMEDS, states that physicians as leaders engage with others to contribute to a vision of a high-quality healthcare system and take responsibility for delivering excellent patient care [16]. Hospitals led by physicians perform better on quality of care but do not outperform CEOs with economic or managerial backgrounds regarding resource management and financial performance [17, 18].

A few studies tried to unravel how LDPs produce organization-level outcomes. Two systematic reviews provide insight into LDPs’ design considerations and the likelihood of achieving these outcomes [2, 5]. Geerts et al. found that LDPs with multiple learning approaches, project work, and mentoring most reliably produce organization-level outcomes [5]. In contrast, Lyons et al. found no clear associations between LDPs’ content and achieving organization-level outcomes [19]. One realist evaluation aimed to describe the impacts of a LDP on participants and the organization [19]. It provided information on how stakeholders’ perceived the working mechanisms of that LDP and insight into critical enablers (e.g., senior management support) and barriers (e.g., time constraints) [19]. However, extensive evidence about how and why LDPs for physicians lead to organization-level outcomes is lacking. Therefore, this study aims to answer the following research question: how, why, and under which circumstances can LDPs for physicians impact organization-level outcomes? LDP providers may use this knowledge to optimize LDPs for physicians and more effectively realize hospitals’ ambitions, including improved patient care.

## Methods

Realist reviews aim to understand how complex interventions work (or not) and how intervention components interact to generate outcomes [13, 20, 21]. Realist reviews are suited when an explorative focus is needed to identify how and why complex interventions work [13, 20, 21]. This is especially true when other methods, such as meta-analyses, are inadequate because interventions are heterogeneous, have multiple components, and are implemented in different organizational contexts [13, 20, 22] – which is the case for LDPs [5]. Both systematic and realist reviews employ a systematic search and screening of the literature [20]. However, whereas systematic reviews focus on determining whether interventions are effective, realist reviews adopt an explanatory analysis discerning why interventions may or may not be successful, under what circumstances, and for whom [20]. Following realist methodology, ‘program theories’ use Context-Mechanism-Outcome (CMO) configurations (hereafter CMOs) to explain how specific contexts (C) and mechanisms (M) work together to generate outcomes (O) [13, 20, 21]. Realist researchers develop program theories at various abstraction levels, ‘normal’ program theories provide the most granular explanations about how and what works in specific settings [13, 23]. Middle-range program theories (MRPT) are more abstract and apply to broader settings [13, 23]. We developed an MRPT, given the heterogeneity in LDPs and the organizational context in which they are conducted.

Developing a program theory helps to determine the review’s scope and structure the findings. Successful realist reviews begin with an *initial* program theory and end with a more *refined* program theory [13, 20, 21]. We inspected relevant systematic reviews to develop our initial MRPT (Supplementary material A) [2, 4–10]. In this initial MRPT, we were able to identify important design choices (e.g., needs assessments), contextual factors (e.g., safe learning environment), and ingredients for potential mechanisms (e.g., improved confidence) to impact organization-level outcomes (e.g., enhanced patient care). However, in this initial MRPT, we could not distinguish between particular contexts and mechanisms that work together to generate specific organization-level outcomes. To answer our research question and refine our MRPT, we designed this study following the RAMESES guidelines [21, 22] and four iterative steps formulated by Pawson et al. [20]. These steps are described below.

### Clarify scope

The research team formulated the research question using realist terminology (how, why, under what circumstances) and investigating the literature on LDPs for physicians. We conducted a pilot search (screening titles and abstract and full text articles), after which we extracted data and conducted an analysis of 10 key articles. Key articles were selected through discussion in the research team, which assessed the articles’ relevance to the research question, link to our initial MRPT, and methodological rigor. The purpose of this was to get acquainted with the literature and potential theories explaining the working of LDPs. Consequently, based on theoretical (e.g., changing perspectives on physician leadership) and practical (e.g., feasibility given the resources of this study) arguments, we decided to narrow the scope of this review, which is considered best practice [21]. We narrowed this review's scope by considering documents published between January 2010 and March 2021 from various hospital settings, e.g., academic, non-academic, public, private. We excluded studies with resident physicians only and those focusing on educational leadership (see Table 1).

**Table 1:** Eligibility criteria

Inclusion	Exclusion
Leadership intervention	Interventions not explicitly using the term leadership or leadership development was a minor objective
Content of the intervention is sufficiently described	Intervention’s content is insufficiently described
Outcomes reported beyond participant satisfaction	No, or very limited, outcome description
Hospital setting	Primary care and other non-hospital settings
At least one physician (in combination with other healthcare professionals e.g., nurses)	No physicians (residents were also excluded)
Published between January 2010 and March 2021^a^	Educational or academic leadership only
	Languages other than English

^a^ None of included documents reported on LDPs conducted within the specific context of the COVID-19 pandemic

### Search for evidence

The search strategy was developed over time in multiple sessions with a librarian (JD). Using VOS viewer [24], we were able to iteratively focus our search by discussing the inclusion or exclusion of several search terms [25]. The findings of our exercises to get acquainted with the literature, pilot screenings, and focusing the scope of this review informed our final search strategy. We searched for various combinations and synonyms of the words: leadership, program, development, and physician in the following databases: Medline, PsycInfo, ERIC, Web of Science and Academic Search Premier. Supplementary material B presents the comprehensive search strategies.

### Appraise primary studies and extract data

#### Eligibility criteria

Table 1 presents the inclusion and exclusion criteria of this review. Only documents (grey literature and scientific studies, hereafter articles or studies) describing and evaluating a leadership intervention were included. We defined a leadership intervention as an educational course, curriculum or program that included one or more interventions for which developing leadership skills, attributes, or competencies was the primary goal. Studies on leadership-related topics, such as collaboration or quality improvement, that did not explicitly use the term leadership were excluded. The LDPs had to focus on physicians working in hospitals: secondary (community hospitals) and tertiary care (academic medical centers and teaching hospitals). Included articles had to describe the interventions’ content sufficiently, i.e., the duration of the intervention, topics addressed, and learning methods used. The results of the leadership intervention had to be reported on a level beyond participant satisfaction (> Kirkpatrick’s level 1) [26].

#### Data screening process

Articles were screened in two steps: 1) title and abstract screening and 2) full-text screening. The main author and a research assistant (ResA) independently screened all titles and abstracts in four batches. MD and ResA resolved conflicts by discussion until consensus. Members of the research team (IJ, KL, WK, KK, YS, MS) were consulted for doubtful cases or when no consensus was reached. Rayyan QCRI software facilitated the title-abstract screening [27].

For full-text screening, MD and ResA used a screening template and independently assessed each eligibility criterion in the following order: 1) physicians, 2) hospital context, 3) leadership intervention (focus), 4) outcomes adequately reported, and 5) intervention sufficiently described. The reviewers terminated full-text screening when a criterion was not met. Full-text articles were screened in six batches, and for doubtful or conflicting cases, the research team was consulted. We adjusted the PRISMA Flow Diagram to present the different phases of our systematic search and screening process [28].

#### Data extraction

Two data extraction forms were developed based on a pilot screening and analysis conducted within the research team. The first extraction form was an Excel file with multiple potential CMOs in the columns and the included studies in rows. From each study, fragments that evidenced the context, mechanism, or outcome were extracted (Table 2). The second form was a table in Word extracting the main study and LDP characteristics. The first author extracted information in batches (five to ten studies), which was verified multiple times by the research team who also read and extracted data fragments for subsets of the data.

**Table 2:** Operationalization of context, mechanism and outcome in this study

Context	Pertains to the relational and dynamic features that shaped the mechanisms through which LDPs work [29]
Mechanism	Mechanisms describe how the resources embedded within a LDP influence the reasoning and behavior of program physicians [30]
Outcome	Refers to intended, unintended, or unexpected program outcomes on various levels, e.g., individual, team, organization [30]. In synthesizing the evidence, we focused on organization-level outcomes

In addition, and in line with realist review guidelines [21], MD assessed the rigor (high/low) and relevance (high/low) of included articles. The assessment of relevance was based on the article’s contribution to the MRPT; rigor was about the trustworthiness of results in relation to the methods used. As systematic reviews indicated that the overall study quality of LDP evaluations is low [2, 4], this exercise was mainly performed to obtain insight into the relative rigor and relevance of the studies in our sample. The most rigorous and relevant articles – both scoring high on rigor and relevance [19, 31–37] – received the most weight during data synthesis.

### Synthesize evidence and draw conclusions

During and after data extraction, an iterative process of theory refinement was performed. This meant integrating and refining all potential CMOs from the data extraction sheet towards a smaller set of CMOs relevant to the abstraction level of the research question, i.e., focusing on explaining organization-level outcomes. The most robust and relevant articles formed the foundation of this reconfiguration exercise and therefore received the most weight. A study provided evidence for a CMO if it included proof of at least the C and M, or C and O, or M and O. Moving up the abstraction ladder was complicated due to the diversity of reported LDP outcomes and the different methods researchers used to classify outcome-levels, e.g., own classification [36] or Kirkpatrick’s approach [34, 35]. Based on discussions within the research team and the iterative process of theory refinement, we identified three organization-level outcomes categories for our final program theory: culture, quality improvement (patient care and organizational processes), and the leadership pipeline. MD led the iterative process of theory refinement which encompassed going back and forth between included studies, extracted documents, and revising CMO formulations. Multiple sessions with the research team were conducted until we reached consensus about a final set of CMOs.

## Results

**Fig. 1:**
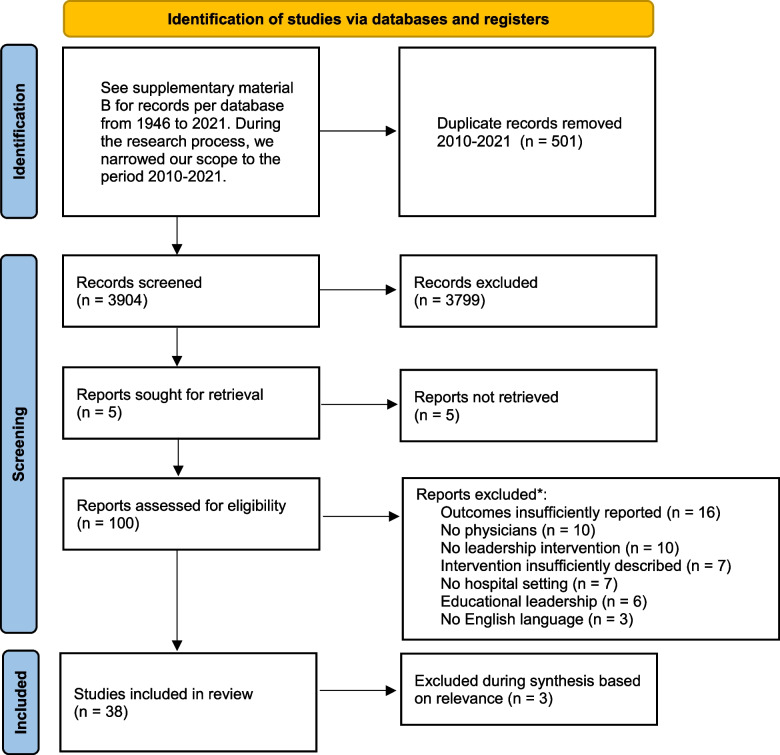
Screening procedure

In total, 3904 titles and abstracts and 100 full-text articles were screened (Fig. 1). Of these articles, 59 were excluded based on exclusion criteria. During the synthesis of evidence, three articles were excluded from the final results because they were not informative to our MRPT based on relevance.

### Characteristics of included LDPs

Thirty-eight articles were included to inform our MRPT (Supplementary material C) [19, 31–67]. These articles reported on 35 unique LDPs,^1^ 23 took place in the United States [31, 33, 34, 36, 37, 39, 40, 43–45, 47, 48, 50, 51, 57–63, 65, 66], 5 in the United Kingdom [19, 35, 41, 54, 64], 2 in the Netherlands [38, 67], 2 in Australia [49, 52], 1 in Canada [53], 1 in Iran [46] and 1 in multiple countries located in Sub-Saharan Africa [42]. Twenty-one LDPs were classified as in-house programs [19, 31, 32, 34, 36, 37, 40, 43, 44, 47, 50, 52, 54, 57–64, 66], meaning that they were conducted within, and developed for, participants from one healthcare institution or system (multiple hospitals within one region). Fourteen LDPs were classified as external programs [33, 35, 38, 39, 41, 42, 45, 46, 48, 49, 51, 53, 65, 67]. The profiles of the (post-residency) physicians participating in LDPs were diverse in terms of medical discipline, being a faculty member, level of seniority, and having a formal leadership role (e.g., medical director). The duration of the LDPs varied from one day [41] to two years [45, 62]. Twenty-seven LDPs had a time span of 6 months or more [19, 32, 34, 35, 37–40, 42–47, 49–54, 57–59, 62, 63, 65, 66]. Most LDP’s primary goal included training physicians to realize organizational change and improve healthcare [19, 31, 33, 36, 38, 39, 42, 46, 48, 49, 54, 57, 59, 60, 63] or prepare them for leadership roles and strengthen the organization’s leadership pipeline [40, 43, 44, 46, 47, 52, 61]. With one exception [41], all LDPs used multiple learning methods with diverse content delivered to participants. Examples of frequently included topics were: leadership theory and styles, quality improvement, health systems, emotional intelligence, group dynamics, negotiating and conflict management, quality improvement, and administrative skills. Studies on LDPs reported a great diversity of outcomes between and within programs, on different levels. For example, Bhalla et al. reported enhanced quality improvement skills and the outcomes of quality improvement projects on diverse domains [39], whereas Berghout’s et al. primary outcome was describing how LDP participation adjusted physicians’ leadership identities [38].

### Middle-range program theory

**Fig. 2:**
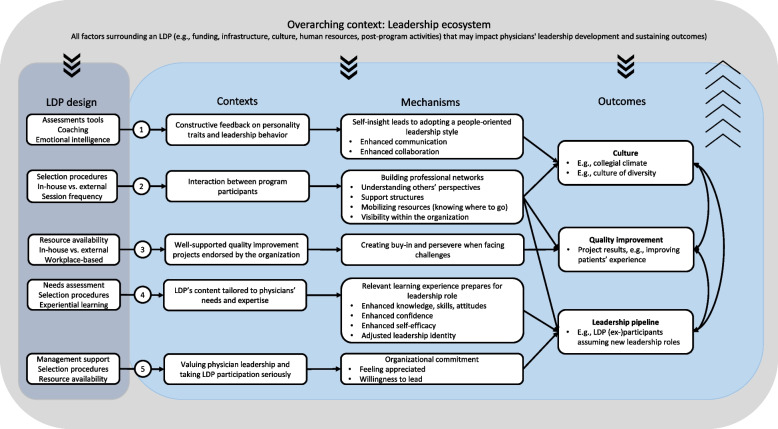
Middle-range program theory

Figure 2 presents our MRPT, which summarizes how, why, and under which circumstances LDPs for physicians can impact the organization-level outcome categories: culture, quality improvement, and the leadership pipeline. Figure 2 shows that LDPs are embedded within and interact with an overarching context: the leadership ecosystem. The leadership ecosystem encompasses all factors surrounding a LDP that may impact physicians’ leadership development and the sustainability of perceived outcomes, including funding, infrastructure (e.g., alignment with other training programs, clear career paths), culture (e.g., recognizing the value of program participants, role models), human resources (e.g., educators, coaches), and post-program activities (e.g., alumni networks, follow-up learnings). Leadership ecosystems aid physicians in transferring their learnings to the workplace after program participation and therefore help to sustain outcomes. Studies of in-house and external LDPs illustrate that adequate leadership ecosystems prevent skill attrition [42, 61], enhance the uptake of leadership behaviors [19, 38, 57], and contribute to the durability of quality improvement projects [39, 51, 59]. Especially LDPs with a relatively long duration or those that were conducted regularly (e.g., annually) seemed to interact with the leadership ecosystem by producing tangible and intangible resources, e.g., quality improvement project outcomes, trained program staff, and networks with external speakers or institutions [31, 36, 59, 60, 63]. The following description illustrates this:

**“**Previous [name LDP] graduates serve as coaches for current attendees, which helps broaden the learning resources for new students and reinforce previous training for coaches.**”** [59]

The leadership ecosystem determines whether LDP providers can introduce intervention components adequately to create a particular learning context for participants. Figure 2 presents critical design aspects of LDPs that contribute to creating specific learning contexts that enhance the likelihood of initiating mechanisms and realizing organization-level outcomes. For example, within some leadership ecosystems, it might be more challenging to create a constructive feedback context through assessment tools than in others. However, the extent to which such a context is realized determines the likelihood of physicians acquiring self-insight and adopting a people-oriented leadership style, benefitting the organization’s culture (CMO 1). Figure 2 presents the five identified CMOs, which are the main body of our program theory. Table 3 spells out each CMO and shows which studies provide supporting evidence for each CMO. The CMOs are depicted linearly to illustrate the main pathways of how and why LDPs achieve organizational outcomes and to be instrumental to LDP providers. The two-way arrows illustrate that the outcome categories may influence each other. In their paper, Smith et al. illustrate the interaction between the outcome categories ‘leadership pipeline’ and ‘the organization’s culture’ [63]:

*“The [name of LDP] was an important vehicle to prepare and promote women for intra-departmental leadership progression, creating role models in leadership positions and thus enhancing the value and culture of the organization.”*

**Table 3:** CMOs and supporting evidence

CMO title and description	Studies that provide (partial) evidence
**Acquiring self-insight and people skills (CMO1)** If LDPs include constructive feedback on physicians’ personality traits and leadership behavior [C], physicians become more self-aware and acquire insight into the needs and preferences of the people they lead. Accordingly, they adopt a people-oriented leadership style which benefits communication and collaboration [M], and thereby the organization’s culture [O]	[19, 32–37, 43–45, 47, 54, 57, 58, 61, 62, 64, 67]
**Intentionally building professional networks (CMO2)** If LDPs stimulate interaction between program participants [C], physicians build professional networks [M], which may impact the organization’s culture [O_1_], quality improvement [O_2_] and the leadership pipeline [O_3_]. When participants are from the same organization, professional networks seem most effective for realizing organization-level outcomes [C] • Due to building professional networks, physicians gain understanding in the perspectives of others (e.g., administrators, other medical disciplines) and collaborate better [M]. Networks also function as support structures [M], benefitting the organization’s culture [O_1_] • Professional networks mobilize resources: physicians know where to go for collaborations or when facing challenges [M], leading to more effective quality improvement [O_2_] • Due to building professional networks, physicians become more visible within the organization and are more likely to be promoted [M], strengthening the organizations’ leadership pipeline [O_3_]	[19, 31, 32, 34–37, 45, 48, 50, 51, 53, 55, 57, 61]
**Supporting quality improvement projects (CMO3)** If LDPs include well-supported quality improvement projects (i.e., coaching or mentoring (hereafter coaching), project management support, funding, protected time, facilities) endorsed by the organization [C], this allows physicians to create buy-in and be more perseverant when facing challenges [M]. This increases the likelihood of successful implementation of the project and quality improvement [O] Note: by quality improvement projects, we refer to a wide range of LDP projects that cover numerous topics. Project topics include reducing patients' waiting time, enhancing internal communication, more competitive purchasing of medical supplies, reducing unnecessary laboratory testing, and standardizing clinical processes	[19, 31, 32, 34, 38–40, 42, 43, 49, 56, 57, 59, 60, 63]
**Tailored LDP content prepares physicians (CMO4)** If LDPs' content is tailored to physicians' leadership needs and expertise [C], physicians perceive the LDP content as relevant, and the learning experience prepares (i.e., knowledge, skills, attitudes, confidence, self-efficacy, identity as leader) them for current or future leadership roles [M]. They are more willing to assume leadership roles and considered competent, leading to new leadership roles and strengthening the leadership pipeline [O]	[19, 31–39, 41, 43, 44, 46, 47, 49, 50, 52, 53, 55, 58, 60–62, 66]
**Valuing physician leaders and organizational commitment (CMO5)** If LDPs reflect that hospitals value physician leaders by facilitating program participation and taking the program seriously [C], physicians feel appreciated, commit to the organization, and are more willing to adopt new leadership roles [M]. This strengthens the leadership pipeline [O], which seems especially true for underrepresented groups in the organization’s leadership pipeline [C]	[19, 31, 32, 34, 36, 43, 44, 50, 52, 61, 63]

In the remainder of this Results section, we elaborate on each CMO.^2^ Supplementary material D provides more precise insight into the data, i.e., original text fragments from included studies, backing up this program theory (CMOs, the interconnectedness of outcomes categories, leadership ecosystems).

#### Acquiring self-insight and people skills (CMO1)

Self-assessments and modules on emotional intelligence or self-awareness gave physicians feedback on their personality traits and leadership behavior [33–36, 43–45, 47, 57, 58, 61, 62, 67]. The perception of a safe learning environment allowed physicians’ to share their feelings [64]. Various tools were used to gather feedback, one example of such a tool is the Dominance Influence Steadiness Contentiousness (DISC)-360 degrees assessment [34, 43]. These tools were most effective when physicians were coached to interpret feedback constructively [19, 43, 45, 57, 58, 64]. Feedback enhanced physicians’ insight into their leadership strengths and weaknesses [57, 58, 61]. In particular, co-workers’ feedback helped physicians to adjust their leadership behavior to the preferences and needs of the people they lead [19, 34–37, 44, 47, 61], i.e., a people-oriented leadership style. For example:

“It made me more confident as a leader and yet more willing to listen to others and give credit to them for their ideas.” [36]

Physicians with a people-oriented leadership style listened better [37, 47, 54, 61, 67] and acknowledged others’ contributions [36, 47]. Consequently, such a leadership style led to more effective communication and collaboration, which benefitted the organization’s culture [19, 33, 36, 37, 43, 47, 58]. For example, Vitous et al. showed that a LDP positively changed the culture within a surgical department due to promoting people-oriented leadership [37]. Rask et al. suggest that a critical mass of LDP (ex-)participants is needed to achieve culture change [60].

#### Intentionally building professional networks (CMO2)

A cohort-based training model, having multiple meetings over time, protected time to interact, and working in teams on projects facilitated interaction between participants [19, 31, 32, 35, 36, 48, 50, 53, 55, 57]. Some LDPs intentionally used these principles to stimulate networking [32, 36, 45, 48, 50, 55], while for others building professional networks seemed an unintended outcome [34, 35, 53, 61]. Other LDP aspects – i.e., in-house vs. external, selecting participants and speakers – influenced the professional networks’ composition and resulting outcomes. For example, in-house programs with participants from multiple departments stimulated interdepartmental networks and collaborations within the organization [19, 36, 57]. In contrast, networks from external programs functioned as a non-power-based source of advice and personal support [48], sometimes with global reach [48], or led to lasting research collaborations [55]. The following illustrates how an in-house LDP stimulated networking:

“I met people who I still have interactions with. That was the best. I made connections helpful from both work and personal perspectives. After 3 or 4 of the meetings, people were comfortable with each other and could say whatever.” [31]

As a result of networking, physicians gained insight into the perspectives of other professions (e.g., managers) and perceived the value of their efforts and skills [19, 31, 34, 37]. Professional networks led to the breakdown of silos between departments and improved inter-professional collaboration [19, 34–36, 57]. These networks established support structures for physicians [34, 35, 48, 61]. Physicians used the obtained contacts to overcome various challenges, start collaborations [31, 34], and avoid duplication of efforts [19]. Lastly, there were indications that professional networks enhanced participants' visibility within the organization, contributing to career advancement [31, 50, 51]. In these ways building professional networks benefitted culture, quality improvement, and the leadership pipeline.

#### Supporting quality improvement projects (CMO3)

LDPs that incorporated quality improvement projects led to improvements in various domains [19, 31, 32, 34, 39, 43, 49, 51, 56, 57, 59, 60, 63], for example: improving efficiency in inpatient or emergency department settings, enhancing transitional care among patients, and reducing hospital-acquired infections or improving sepsis care [39]. Quality improvement projects were described as innovation incubators as they led to innovative ideas to combat healthcare organizations' challenges [40, 43, 57].

Quality improvement projects that were in line with the strategic priorities of the organization were more likely to be successful [19, 32, 39, 43, 51, 56, 59, 60, 63]. This is because such alignment enabled physicians to obtain buy-in from management, project funding, and other required resources [19, 43, 51, 60]. In-house LDPs facilitated alignment between projects and institutional priorities [31, 32, 34, 43, 59, 60]. Matching physicians’ motivation and institutional priorities took up to 8 weeks in a LDP with project work in external organizations [56]. Ongoing project management or coaching allowed physicians to create buy-in among colleagues and persevere when facing implementation barriers [19, 32, 38, 39, 42, 51, 56, 60]. Daniels’ et al. study further illustrates this [42]:

*“She [the participant] referenced the important ongoing support her mentor provided in helping her implement the project. She stated that ‘My primary mentor was an obstetrician like myself....he would sit down with me to figure out what needed to be done [on the project]”*

Adequate support from colleagues and management, and the availability of sufficient resources (e.g., funding, protected time, facilities) were critical for projects’ quality improvement success [19, 38, 39, 42, 51, 56, 57, 59]. When support and resources diminished or were taken away altogether, often after LDP completion, positive outcomes faded or projects ceased to exist [19, 38, 39, 42, 51, 56, 57, 59].

#### Tailored LDP content prepares physicians (CMO4)

LDP providers tailored LDPs’ content to physicians’ needs and expertise by employing needs assessment and rigorous selection and nomination procedures [32, 37, 39, 43, 44, 46, 47, 53, 58, 66]. To this end, longitudinal programs used participating physicians’ feedback [31, 36, 39, 43, 48, 55, 58, 59, 62, 65]. Two LDPs had separate tracks with different content based on physicians’ leadership expertise [60, 66]. Experiential learning methods accommodated physicians’ needs for leadership development in the workplace [32, 35, 36, 41, 43, 49, 55, 60].

Tailored LDP content prepared physicians for leadership roles because the content was perceived as relevant and required for adequate professional performance [19, 31, 33, 34, 41, 47, 49, 52, 66]. Physicians who found the content irrelevant did not perceive LDP’s to be beneficial [19, 34, 37]. After LDP participation, physicians reported enhanced knowledge and skills, attitudes [19, 31–37, 39, 41, 43, 44, 46, 47, 49, 50, 53, 58, 60, 61, 66], organizational literacy [35, 36, 49, 53], confidence and self-efficacy as a leader [34, 35, 41, 47, 58]. Moreover, researchers investigating two specific LDPs, reported that physicians’ leadership identity shifted: from an ‘individualistic’ towards a more ‘collaborative’ identity [38, 62]. Physicians were also motivated and considered competent to assume new leadership roles [19, 31, 33–36, 44, 52, 55, 60, 61, 66]. The study of Fernandez et al. illustrates further [33]:

“*Interestingly, a large majority of respondents reported receiving a promotion or other similar expansion of role opportunity since completing the course, and all who reported such a job expansion indicated that the skills learned in the course helped prepare them for the new opportunity.*”

Supporting physicians after participation in a LDP, e.g., through coaching or formalized career trajectories, seemed vital for physician leaders to remain at the organization and be willing to assume leadership positions [19, 31, 32, 38, 57].

#### Valuing physician leadership and organizational commitment (CMO5)

Physicians felt honored to be selected for LDPs [34, 43] and recognized the opportunity to participate as a sign that the organization believed in them [44, 50]. They considered LDPs to symbolize the hospital's investment in developing its ‘own’ leaders [36, 44]. The competitiveness and prestige of LDPs enhanced these perceptions [32, 36, 43, 44]. According to physicians, the presence and support of (senior) management during LDPs showed that the organization regarded leadership development as a priority [19, 52]. The availability of adequate resources indicated that the organization earnestly invested in leadership development [19, 43]. As a result, physicians felt more connected and committed to the institution [32, 34, 43] and assumed new leadership roles [19, 31, 34, 36, 44, 52, 63]. The following description illustrates this CMO:

“*Across interviews, the participants stated that they felt their involvement in the leadership development program was an investment by the Center in their personal development and growth. They perceived that the investment meant that the Center believed in them. The participants agreed that their engagement was positively impacted by this perception. This is important to physicians because they do not like to stay in the wrong or be unappreciated.*” [44]

Underrepresented groups in leadership positions, felt especially appreciated and assumed new leadership roles [31, 36]. Several LDPs targeted underrepresented groups to achieve a more diverse leadership pipeline [31, 36, 50, 61, 63].

## Discussion

### Main findings – middle-range program theory

This study resulted in a MRPT explaining how, why and under which circumstances LDPs for physicians impact organization-level outcomes. The MRPT presented considers three organization-level outcome categories: culture, quality improvement, and the leadership pipeline. For enhancing culture, a person-oriented leadership style and professional networks were important mechanisms triggered respectively by contexts that provided physicians with feedback on their leadership style and facilitated interaction between participants. Well-supported quality improvement projects endorsed by the organization enabled physicians to create buy-in and persevere when facing challenges, increasing the likelihood of quality improvement. Also, professional networks aided quality improvement by mobilizing resources within organizations. LDPs enhanced the organization's leadership pipeline by preparing physicians for leadership roles. Tailoring LDP’s content to physicians' needs and expertise facilitated the firing of this mechanism. Organizations showing appreciation of physician leaders through LDPs, promote commitment of the physicians to the organization and thereby strengthen the leadership pipeline. Professional networks benefitted the leadership pipeline because potential physician leaders gained visibility. Lastly, the leadership ecosystem is crucial to realizing and sustaining organization-level outcomes.

### Explanation of main findings

We further explain our MRPT by elaborating on the concept of a ‘leadership ecosystem’ and the five identified CMOs. Other researchers have recommended that healthcare organizations view leadership development as an ecosystem rather than an isolated course or program [68]; our MRPT reveals why considering the leadership ecosystem is crucial for impacting organization-level outcomes. In this study, the leadership ecosystem encompassed all factors surrounding a LDP that may impact physicians' leadership development and sustaining organization-level outcomes, including funding, infrastructure, culture, human resources, and post-program activities. By considering the leadership ecosystem, LDP providers can ensure that LDPs’ objectives match the resources available. Moreover, adequate leadership ecosystems help physicians transfer LDP learnings to the workplace. A LDP interacts with the surrounding leadership ecosystem, for example, when alumni serve as coaches for the next cohort of participants [59, 63]. Von Thiele et al. confirm the importance of an ecosystem for designing interventions with maximum impact [69]. Other studies report the constraints of inadequate leadership ecosystems, such as physicians intending to leave the organization because their skills are underutilized [35, 38]. Therefore, it is crucial to consider leadership development in its broader context.

The finding ‘*Acquiring self-insight and people skills (CMO1)*’ confirms that co-workers’ feedback is crucial for enhancing physicians’ professional performance, especially regarding people skills [12, 70]. For LDPs in hospitals where physicians are not used to receiving feedback, it might be more challenging to create a constructive feedback context than in hospitals where physicians regularly receive feedback on their professional performance [71, 72]. In many healthcare systems, feedback systems and cultures have been implemented with positive effects on physicians’ professional development and performance [72–74]. In this review, co-workers’ feedback gave physicians insight into their leadership style and the needs of the people they lead, making them adopt a people-oriented leadership style. Researchers have shown that people-oriented leadership styles can promote positive workplaces and enhance healthcare professionals’ occupational well-being [75–78]. People-oriented leadership styles among physicians may be underdeveloped as traditional medical training generally devotes little attention to these skills; it usually focuses on solving medical problems and autocratic leadership in emergencies [78, 79]. However, since the introduction of competency-based professionalism frameworks, medical schools are revisiting curricula to pay attention to novel leadership competencies. While one leadership style is not universally best – a crisis may require a directive style [79] – LDPs seem able to identify and address underdeveloped leadership competencies in physicians – people skills – with benefits to the organization.

Interestingly, ‘*Intentionally building professional networks (CMO2)*’ impacted all outcome categories. Leadership development researchers consider intentionally building communities as best practice [9]. According to social capital theory (SCT), professional networks are social capital as they produce resources relevant to the individual and organization [80]. For example, we found that professional networks provide a support structure for physicians and mobilize resources for quality improvement. Three types of social capital can be distinguished: bonding social capital (e.g., relationships between physicians from one department), bridging social capital (e.g., relationships between physicians from different departments or organizations), and linking social capital (e.g., relationships physicians and the hospital's board (different hierarchical levels)) [81]. Based on our findings, LDP providers may develop a particular 'type' of social capital via recruitment and selection of participants. Also, professional networks seemed mainly beneficial to organization-level outcomes when participants came from the same organization. Professional networks with people from other organizations may gain importance as hospitals and policymakers see inter-organizational collaborations as a way to promote the quality and cost-efficiency of patient care [82–84].

Previous researchers have shown the importance of quality improvement work in LDPs for realizing organization-level impact [2, 4, 5]. The finding ‘*Supporting quality improvement projects (CMO3)*’ confirms this and simultaneously shows that quality improvement projects are only likely to succeed with adequate support (e.g., coaching, protected time). Physicians working in cultures where their colleagues regard medical tasks as superior and relatively unrelated to quality improvement may face the most resistance to implementing quality improvement projects [38, 85]. While there is abundant evidence that quality improvement projects can effectively target specific organizational priorities [5, 39, 59], hospitals should not include them in LDPs without careful thought. This is because sufficiently supporting quality improvement projects requires significant resources per participant (high dose [86, 87]). As a result, these LDPs often target high-potential physician leaders to participate (low reach [86, 87]). Some LDP providers may reduce the resources spent per participant (low dose) to reach a larger number of physicians (high reach) and a critical mass within the organization. Strategic HRM researchers suggest that different development approaches are needed based on the uniqueness and expected return of the human capital to be developed [88]. Providers of LDPs for physicians may use such frameworks for determining the optimum between reach and dose concerning realizing organization-level goals.

Our result, ‘*Tailored LDP content prepares physicians (CMO4)*’, shows LDPs' ability to realize one of its primary purposes: to prepare physicians for their leadership roles and strengthen the organization's leadership pipeline. Notably, this CMO shows that a ‘whole-person’ approach to developing and preparing physician leaders is needed. Such an approach includes attention to physicians’ knowledge, skills and attitudes, leadership identity, confidence, and self-efficacy as a leader. Previous studies have recommended incorporating insights from professional identity formation theory into leadership development interventions [38, 89, 90]. Bandura’s self-efficacy theory [91] and review findings [92] show that confidence and self-efficacy are vital to effective leadership. Physicians with high self-efficacy view issues at work as challenges that should be managed rather than avoided due to perceptions of inadequate skills [91]. This aligns with the concepts of resilience and a growth mindset, as physicians possessing these qualities have the resources to deal with adversities and see them as an opportunity to learn and grow [93, 94]. When physicians believe in themselves and grow as leaders, they are more willing to expand their leadership roles. They experience that they can change things they thought were unchangeable, indicating a sense of psychological ownership over the working environment, which has its roots in self-efficacy, self-identity, and belonging [95, 96]. Psychological ownership may benefit organizational performance and physicians’ well-being and may develop due to enhanced organizational literacy or expanded responsibilities in leadership roles [95, 96].

According to Meyer and Allen, affective commitment refers to the employee’s emotional attachment to, identification with, and involvement in, the organization [97]. Employees with strong affective commitment continue employment with the organization because they want to. According to the literature, this is based on an exchange relationship [97, 98], reflecting our finding ‘*Valuing physician leadership and organizational commitment (CMO5)*’. This CMO showed that when physicians felt valued as leaders by the organization through investing in their professional growth, they reciprocated this favor by assuming leadership roles. CMO5 may become even more critical for the next generation of physicians as they highly value personal development and sense-making [99]. Surprisingly, this exchange mechanism seemed most effective for underrepresented groups in leadership positions, e.g., women and participants from the Asian Pacific region. These groups may not have experienced the same leadership opportunities due to ingrained institutional disadvantages [100, 101]. Striving for equal leadership opportunities for all individuals regardless of their gender or background is not only the morally right thing to do, but establishing an inclusive and diverse leadership pipeline should also be high on hospitals’ agendas considering organizational performance [102, 103].

### Strengths and limitations

This realist review is the first to comprehensively investigate why and how LDPs for physicians impact organization-level outcomes in hospital settings. One strength of this realist review was the diverse research team with expertise in physicians’ professional performance, medical education, leadership development, strategic human resources, sociology, and realist review methodology. Other strengths were conducting a pilot search, screening and analyzing key articles, and developing our search strategy iteratively with a librarian.

The results of this realist review should be considered in light of several limitations. First, the generalizability of our MRPT to other healthcare professions and settings may be limited due to its focus on physicians, the hospital setting, and the fact that most included studies come from Western countries. Mainly including studies from Western countries, brings a particular perspective on leadership, which might have influenced our findings. On the other hand, our MRPT has a higher abstraction level than ‘normal’ program theories and applies across broader settings [23]. Moreover, multiple LDPs included physicians and other healthcare professionals as participants (see Supplementary material C). Additional research is needed to investigate whether our program theory is generalizable to other health professions and settings.

Second, the publication bias in the literature about LDPs for physicians might have influenced our results [2, 4]. Our program theory may not fully capture the aspects of LDPs that do not work and might overestimate the likelihood of some contexts and mechanisms to produce outcomes. However, all the mechanisms found are grounded in broader theoretical perspectives.

A third limitation of this study pertains to the lack of grey literature. Most included documents were scientific articles, although we also incorporated grey literature [19, 57, 62]. While our search strategy enabled for retrieval of grey literature within the inspected databases, there is much information available on the internet about LDPs. Inspecting all these sources was deemed unfeasible given the resources of this study.

### Implications for research and practice

This study presented a LDP MRPT about the working of LDPs for physicians in hospital settings regarding organization-level outcomes. Researchers could further verify and refine our MRPT for physicians and other healthcare professionals. For example, they could investigate the relative strengths of the CMOs found and start with investigating more fine-grained CMOs, i.e., linked to specific outcomes at the organizational level. High-quality LDP evaluations facilitate this endeavor. We encourage researchers and LDP providers to employ methods such as realist evaluations to collect more precise knowledge on contextual factors, working mechanisms, and program aspects that do not work. More insight into specific inputs (e.g., costs, time investments) in relation to LDP outcomes is needed as it allows for better judgments on ‘what works’. Only a few studies indicated LDP’s costs [34, 39, 43, 52, 56], an important aspect of effective programming. Also, more objective data on LDP outcomes are welcomed in addition to most self-perceived evidence, for example, comparing the promotions of physician LDP participants against a control group to evaluate improvements in the leadership pipeline.

Another important direction for future research is the relationship between LDP participation and physicians’ well-being [75, 104]. In this review, well-being outcomes were largely absent, which is surprising, given the alarming burnout levels reported among physicians [105]. Professionally fulfilled physicians are needed to navigate challenging healthcare developments, such as aging populations with comorbidities [106]. Future research could specifically investigate the effects of LDPs on the well-being of physicians and the people they lead. Moreover, these studies can consider adverse well-being outcomes, e.g., enhanced workload due to the LDP or dissatisfaction due to peers not recognizing their leadership qualities.

Furthermore, the results add to the existing literature by not only revealing what ‘ingredients’ may be needed for effective LDPs, but also describing ‘how to prepare the meal’. That is, *how* and *why* LDP aspects in certain contexts trigger mechanisms and generate results. Improved understanding of LDPs for physicians may enable LDP providers to develop more effective LDPs and fit-for-purpose evaluations. According to our MRPT, the following topics are essential for LDPs for physicians in hospital settings aiming to impact organization-level outcomes: 1) acquiring self-insight and people skills, 2) intentionally building professional networks, 3) supporting quality improvement projects, 4) tailoring LDP’s content to physicians’ needs and expertise, 5) valuing physician leadership and organizational commitment, and 6) ongoing leadership development embedded in a leadership ecosystem. Guidelines for designing effective leadership interventions [5, 9, 69] recommend related topics and may also be instrumental to LDP providers. In addition, LDP providers could include modules on healthcare providers' well-being and incorporate positive (e.g., professional fulfillment) and negative (e.g., burnout) well-being indicators in the program evaluation.

## Conclusions

This study offers a MRPT explaining how, why, and under which circumstances LDPs for physicians impact the organization-level outcomes: culture, quality improvement, and the leadership pipeline. The MRPT includes one overarching context, the leadership ecosystem, and five CMOs. Ongoing leadership development within a leadership ecosystem is crucial to realizing and sustaining organization-level outcomes. Moreover, creating learning contexts that fire the working mechanisms of LDPs often requires adequate support and resources for participating physicians. This MRPT may guide the development of LDPs for physicians to realize specific hospital ambitions effectively. Hospitals need a solid physician leadership pipeline to cope with major developments in healthcare. By valuing physician leaders and investing in their leadership development, hospitals can create a cadre of physician leaders who want to go the extra mile for the organization and the patients they serve.

## Supplementary Information

**Additional file 1: Supplementary material A.** Initial middle-range program theory.

**Additional file 2: Supplementary material B.** Comprehensive search strategies.

**Additional file 3: Supplementary material C.** Characteristics of included LDPs and studies. Note: subsequent rows with a light grey background indicate that the studies in these rows describe the same LDP.

**Additional file 4: Supplementary material D.** Evidence backing up MRPT. Note: we do not exhaustively give all possible available fragments from studies that might support our MRPT. Note: we included studies in this table if they included information on the C and M, or C and O, or M and O, of the proposed CMO.

## Data Availability

All data generated or analyzed during this study are included in this published article [and its supplementary information files].

## Footnotes

1 Here we cited Hopkins et al. for the Stanford Leadership Development Program and Daniels et al. for the Afya Bora Fellowship as more than one study reported on these LDPs.

2 Note that referencing may not be exhaustive. In the process of theory refinement, the main goal was backing up the program theory with supporting evidence and not counting the frequency of occurrence.
