# Supplementary figures and images for: Linking leadership development programs for physicians with organization-level outcomes: a realist review
Source: BMC Health Serv Res. 2023 Jul 21;23:783. doi: 10.1186/s12913-023-09811-y (PMC10362722; doi:10.1186/s12913-023-09811-y)

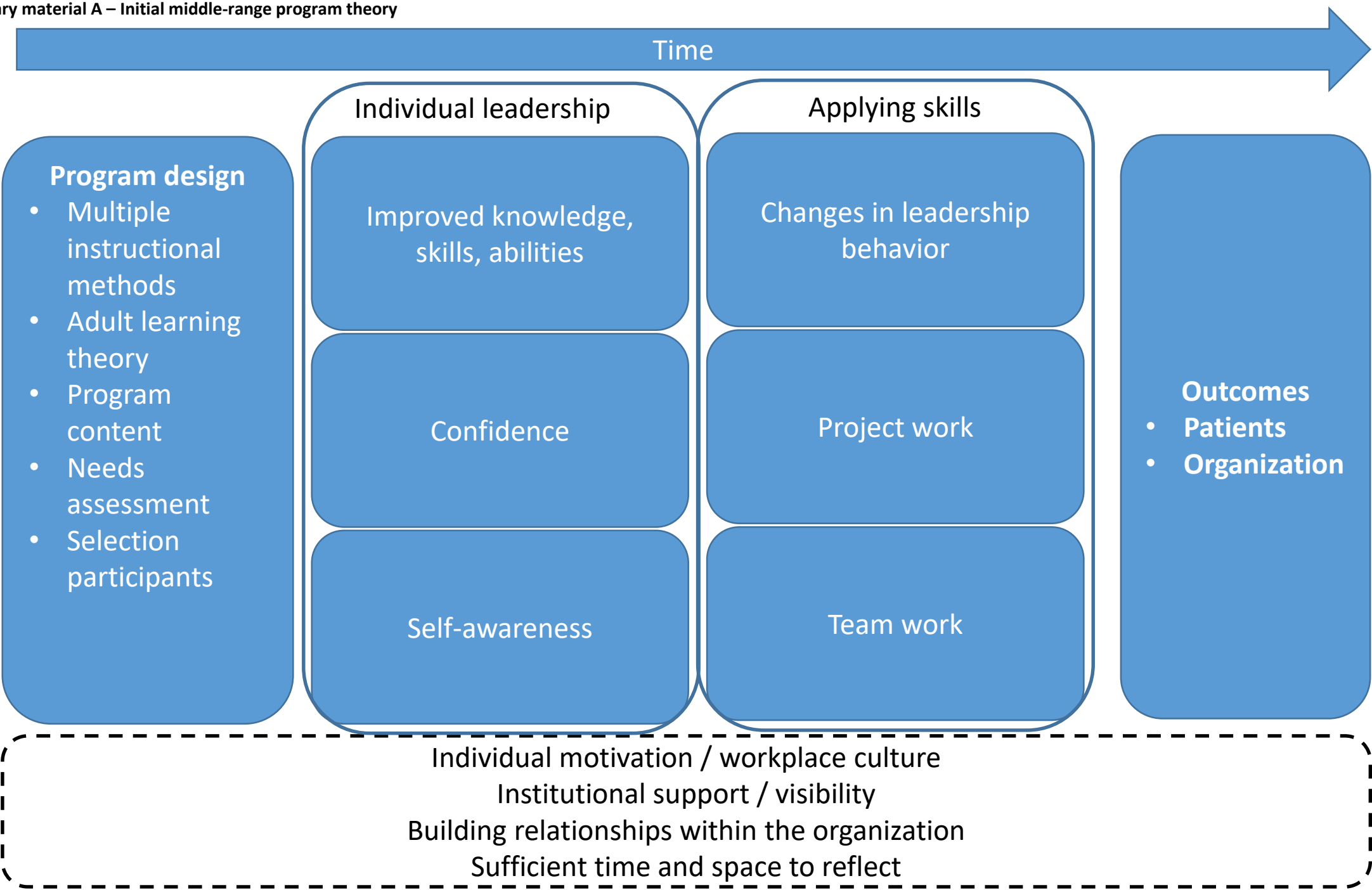

Supplement: Supplementary file 1 — Additional file 1: Supplementary material A. Initial middle-range program theory. [file 12913_2023_9811_MOESM1_ESM.pdf]
