# Supplementary material for: Linking leadership development programs for physicians with organization-level outcomes: a realist review
Source: BMC Health Serv Res. 2023 Jul 21;23:783. doi: 10.1186/s12913-023-09811-y (PMC10362722; doi:10.1186/s12913-023-09811-y)
Supplement: Supplementary file 2 — Additional file 2: Supplementary material B. Comprehensive search strategies. [file 12913_2023_9811_MOESM2_ESM.pdf]

**Supplementary material B – Comprehensive search strategies**

| | **Ovid MEDLINE(R) ALL <1946 to March 19, 2021>**<br>**Search date: 22 March 2021** | |
|---|---|---|
| **#** | **Searches** | **Results** |
| 1 | (((manager? or management or leadership or leader?) adj3 (programme? or program? or train* or workshop or develop* or academy or track?)) or mba or "master of bu?siness").ab,kf,ti. | 49389 |
| 2 | (physician? or clinician? or fellow* or clinical leader* or medical leader* or faculty or doctor?).ab,hw,kf,ti. | 974791 |
| 3 | (evaluat* or assess* or survey? or inventor* or questionnair* or effect* or outcome? or quality).ab,hw,kf,ti. | 13325950 |
| 4 | and/1-3 | 6513 |
| 5 | limit 4 to yr="2000-current" | 5624 |
| 6 | (Symptom? or Drug? or side effect?).ti. | 555374 |
| 7 | (syndrome? or heart failure or adhd or comorbidity or dose or Fracture or osteoporosis or algorithm or disease management programme or coronary heart disease or athlete or agent or prognosis or pregnancy or pharmacotherapy or dmp).ab,kf,ti. | 3949411 |
| 8 | or/6-7 [VOS cluster 2] | 4414821 |
| 9 | (airway management or airway or intubation or ems or difficult airway or airway management training or household or mosquito or simulator).ab,kf,ti. [VOS cluster 3] | 315470 |
| 10 | (primary care clinic or obesity or diabetes or body mass index or diabete or bmi or diet or weight loss or weight management program or obese patient or weight management progamme or hypertension or blood pressure or self management support or physical activity or stroke or glycemic control).ab,kf,ti. [VOS cluster 4] | 1963702 |
| 11 | (medication therapy management or mtm or beneficiary or medical home or pcmh or nursing home resident or chronic disease management pro or accountable care organization).ab,kf,ti. [VOS cluster 6] | 8691 |
| 12 | or/8-11 [VOS NOTing out] | 6150981 |
| 13 | 5 not 12 | 4038 |
| 14 | ("leader's edge" or "coaching and leadership" or (doctor? and equip)).ab,kf,ti. | 158 |
| 15 | 2 and "leaders growing leaders".mp. | 1 |
| 16 | or/13-15 | 4188 |

| | Ovid APA PsycInfo <1806 to March Week 3 2021><br>Search date: 22 March 2021 | |
|---|---|---|
| **#** | **Searches** | **Results** |
| 1 | (((manager? or management or leadership or leader?) adj3 (programme? or program? or train* or workshop or develop* or academy or track?)) or mba or "master of bu?siness").ab,id,ti. | 35850 |
| 2 | (physician? or clinician? or fellow* or clinical leader* or medical leader* or faculty or doctor?).ab,hw,id,ti. | 220182 |
| 3 | (evaluat* or assess* or survey? or inventor* or questionnair* or effect* or outcome? or quality).ab,hw,id,ti,tm. | 2763481 |
| 4 | and/1-3 | 2070 |
| 5 | limit 4 to yr="2000-current" | 1832 |
| 6 | (Symptom? or Drug? or side effect?).ti. | 107423 |
| 7 | (syndrome? or heart failure or adhd or comorbidity or dose or Fracture or osteoporosis or algorithm or disease management programme or coronary heart disease or athlete or agent or prognosis or pregnancy or pharmacotherapy or dmp).ab,id,ti. | 358558 |
| 8 | or/6-7 [VOS cluster 2] | 447380 |
| 9 | (airway management or airway or intubation or ems or difficult airway or airway management training or household or mosquito or simulator).ab,id,ti. [VOS cluster 3] | 33099 |
| 10 | (primary care clinic or obesity or diabetes or body mass index or diabete or bmi or diet or weight loss or weight management program or obese patient or weight management progamme or hypertension or blood pressure or self management support or physical activity or stroke or glycemic control).ab,id,ti. [VOS cluster 4] | 179896 |
| 11 | (medication therapy management or mtm or beneficiary or medical home or pcmh or nursing home resident or chronic disease management pro or accountable care organization).ab,id,ti. [VOS cluster 6] | 2285 |
| 12 | or/8-11 [VOS NOTing out] | 625110 |
| 13 | 5 not 12 | 1510 |
| 14 | ("leader's edge" or "coaching and leadership" or (doctor? and equip)).ab,id,ti. | 160 |
| 15 | leaders growing leaders.mp. | 0 |
| 16 | or/13-15 | 1665 |

| | Web of Science<br>Search date: 22 March 2021 | |
|---|---|---|
| **#** | **Searches** | **Results** |
| 1 | TS=(((manager? or management or leadership or leader?) NEAR\2 (programme? or program? or train* OR workshop or develop* or academy or track?)) or mba or "master of bu?siness") | 5071 |
| 2 | TS=(physician? or clinician? or fellow* or clinical leader* or medical leader* or hospital? or medicine or doctor?) | 1173522 |
| 3 | #1 AND #2 Indexes=SCI-EXPANDED, SSCI, A&HCI, ESCI Timespan=20102021 | 91 |

| | Ovid ERIC <1965 to January 2021><br>**Search date: 22 March 2021** | |
|---|---|---|
| **#** | **Searches** | **Results** |
| 1 | (((manager? or management or leadership or leader?) adj3 (programme? or program? or train* or workshop or develop* or academy or track?)) or mba or "master of bu?siness").tw. | 33652 |
| 2 | (physician? or clinician? or clinical leader* or medical leader* or doctor? or hospital?).tw. | 21251 |
| 3 | (evaluat* or assess* or survey? or inventor* or questionnair* or effect* or outcome? or quality).tw. | 950958 |
| 4 | and/1-3 | 280 |
| 5 | limit 4 to yr="2000-current" | 143 |

| | **Ebscohost Academic Search Premier**<br>**Search date: 22 March 2021** | |
|---|---|---|
| **#** | **Searches** | **Results** |
| 1 | AB (((manager? or management or leadership or leader?) n/2 (programme? or program? or train* OR workshop or develop* or academy or track?)) or mba or "master of bu?siness") | 4135 |
| 2 | TI (((manager? or management or leadership or leader?) n/2 (programme? or program? or train* OR workshop or develop* or academy or track?)) or mba or "master of bu?siness") | 1138 |
| 3 | SU (((manager? or management or leadership or leader?) n/2 (programme? or program? or train* OR workshop or develop* or academy or track?)) or mba or "master of bu?siness") | 143 |
| 4 | KW (((manager? or management or leadership or leader?) n/2 (programme? or program? or train* OR workshop or develop* or academy or track?)) or mba or "master of bu?siness") | 354 |
| 5 | S1 OR S2 OR S3 OR S4 | 4621 |
| 6 | AB (physician? or clinician? or fellow* or clinical leader* or medical leader* or hospital? or medicine or doctor?) | 1271037 |
| 7 | TI (physician? or clinician? or fellow* or clinical leader* or medical leader* or hospital? or medicine or doctor?) | 289798 |
| 8 | SU (physician? or clinician? or fellow* or clinical leader* or medical leader* or hospital? or medicine or doctor?) | 743099 |
| 9 | KW (physician? or clinician? or fellow* or clinical leader* or medical leader* or hospital? or medicine or doctor?) | 183018 |
| 10 | S6 OR S7 OR S8 OR S9 | 1813536 |
| 11 | S5 AND S10 - Publication date: 20000101-20210430 | 220 |
