# Supplementary material for: Linking leadership development programs for physicians with organization-level outcomes: a realist review
Source: BMC Health Serv Res. 2023 Jul 21;23:783. doi: 10.1186/s12913-023-09811-y (PMC10362722; doi:10.1186/s12913-023-09811-y)
Supplement: Supplementary file 3 — Additional file 3: Supplementary material C. Characteristics of included LDPs and studies. Note: subsequent rows with a light grey background indicate that the studies in these rows describe the same LDP. [file 12913_2023_9811_MOESM3_ESM.pdf]

Supplementary material C – Characteristics of included LDPs and studies

Note: subsequent rows with a light grey background indicate that the studies in these rows describe the same LDP.

No.	First author, year	Name LDP	Country LDP	In-house LDP (Yes/No)	Duration LDP	Main goal LDP and study aim	Content and learning methods LDP	Participants selected (Yes/No)	Participants LDP	Main outcomes
1	Berghout, 2020	Medical leadership development programme (MLDP)	The Netherlands	No	1-year (six collective sessions (total of 9 days) and three 2-hour in-house hospital sessions)	LDP goal: To enable physicians to take the lead in the continuous improvement of healthcare.<br><br>Study aim: To illustrate how physicians, by means of participating in a MLDP, try to undo their often-assumed stable professional identity by reinterpreting their relational position towards hospital contexts to better deal with perceived institutional pressures.	Module 1: Introduction, theory on leadership styles, group discussion about medical leadership, personal reflection exercise.<br><br>Module 2: Theory on quality dimensions, theory on short-cycle improvements, individual exercise in applying these on personal projects.<br><br>Module 3: Guest speaker on leadership in 'high performance organisations', exercise in applying insights on personal projects.<br><br>Module 4: Guest speaker (physician) on personal leadership, guest speaker (psychologist) on relations in teams, session with directors on lifestyle and prevention.<br><br>Module 5: Guest speakers (director top-clinical hospitals, patient) on shared decision-making, guest speaker (director Dutch inspectorate) on policy-making and accountability, guest speaker (financial advisor) on healthcare costs and efficiency, expert panel healthcare entrepreneurs.<br><br>Module 6: Guest speakers (two physicians, one nurse) about their role as 'medical leader', guest speaker (director Dutch Council for Public Health and Society).<br><br>In-house sessions (3): participants present the developments of their individual improvement projects to hospital directors, facilitators and Peers.<br><br>In addition to the collective sessions, every participant carried out a hospital-based improvement project.	Yes	23 physicians (six hospitals) representing 13 different medical disciplines. (September 2017–July 2018).	The results illustrate how physicians initially construct conflicting leadership narratives – heroic (pioneer), clinical (patient's guardian) and collaborative (linking pin) leader – in reaction to changing organisational and clinical demands.
2	Bhalla, 2018	Clinical Quality Fellowship Program (CQFP)	United States	No	15 months (see article for frequency, days per year of sessions)	LDP goal: To develop necessary skills in competitively selected physician and nurse "quality champions," and to equip them with the necessary knowledge and support to promote and sustain quality improvement in their clinical settings.<br><br>Study aim: One goal of this article is to describe the curricular structure and key features of the CQFP. Accordingly, additional goals of this article are to share information on participants'	1. *Off-site didactic and participatory training:* The program begins with 4 full-day training "retreat" sessions, with in-depth instruction on relevant quality improvement and patient safety topics, through a combination of didactic content, group exercises, and role-play scenarios.<br><br>2. *Longitudinal and interactive learning*: Following the initial training retreats, interactive educational webinars and in-person sessions are held to continue instruction about quality leadership, quality improvement tools and techniques, and relevant and timely health care issues. Fellows complete 5 homework assignments during the program, which include individual and group work.	Yes	Of the 89 fellows during the period, 75 (84%) were physicians, and the remainder were nurses. Seventy-four physician fellows represented multiple medical specialties as follows The 89 fellows represented 45 hospitals or organizations affiliated with hospitals across the greater New York region. The annual number of fellows by	Among program participants completing self-assessment evaluations, significant improvements were observed across all quality improvement skill areas. Capstone project categories included inpatient efficiency, transitional care, and hospital infection. Fifty-six percent of participants obtained promotions following program completion.

						perceptions of the effectiveness of the CQFP, to describe improvement efforts initiated at participant organizations as part of the CQFP, and to describe pertinent career advancements of participants following program completion.	3. *Applied project and mentorship:* Fellows are required to design and lead a quality improvement project—the capstone. The project is conducted with an interdisciplinary team of clinicians at the fellows' home institutions to advance organizational or departmental quality or patient safety goals.		year was as follows: 16 (in 2010), 15, 18, 20, and 20 (in 2014).	
3	Christensen, 2016	Leading in Health Care (LHC)	United States	Yes	10 months (10 full-day sessions)	LDP goal: Somewhat unclear, two goals: leadership pipeline and innovation incubator.<br><br>Study aim: To describe the rationale for and spectrum of leadership development programs, highlighting experience at a large healthcare institution.	The curriculum of the course is highly participatory and is organized around three themes:<br><br>1. Organizational mission, vision, and values, as informed by a series of conversations with organizational<br>leaders and discussions about organizational history and culture.<br><br>2. Fundamentals of health care finance (including how to develop a formal business plan).<br><br>3. Organizational development, which includes emotional intelligence, teambuilding, strategic planning, conflict resolution, and executive presentation skills, among others.<br><br>In addition to the skill-based sessions listed above, 360° feedback evaluations provide insight to each attendee, while also providing an opportunity to receive executive coaching on the same.<br><br>Teams developing a full business plan over 10 months.	Yes	As originally offered, the course enrolled physicians.<br><br>The course was expanded to its current interdisciplinary cohort of approximately 70 physicians, nurses, and administrators.<br><br>Leading in Health Care is currently in its 13th year.	Perhaps the greatest indicator of the program's impact and success is that 43% of participants have been promoted to leadership positions at Cleveland Clinic over the ensuing decade, and an analysis that shows that the baseline emotional intelligence correlates of longterm promotion are having self-confidence, being a change catalyst, and having an achievement orientation.
4	Cohen, 2019	The Crucible	United Kingdom	No	1 day	LDP goal: To help develop clinical leadership skills and understanding of the new NHS structure .<br><br>Study aim: This study aims to test for first time if participation in a behavioral simulation is an acceptable and effective modality to improve clinical management and leadership capability and the understanding of health care reforms.	A single-day behavioral role-playing simulation. Participants are told in their briefing pack that the poor performance of health services in the boroughs has been highlighted in a recent television documentary in which stakeholders were interviewed to provide a synopsis of current tensions and challenges facing health care in the region. Statements from stakeholders describing their positions and concerns are provided to participants. The local council and health groups are keen that this escalating situation is resolved and plans to improve health care delivery are developed; this is the setting for The Crucible simulation.	No	Of the 69 participants, 32 were currently working at Consultant grade and 35 at Registrar grade. Two participants did not identify their current grade. Participants came from a wide range of specialties, including medicine, surgery, public health, dentistry, psychiatry, and anaesthetics.	Significant improvements were shown in perceived knowledge, capability, attitudes, subjective norms, intentions, and leadership competency following the program. Nearly one third of participants reported that they had implemented knowledge and skills from the simulation into practice within 4 weeks.
5	Daniels, 2014	Afya Bora Fellowship	Botswana, Kenya, Uganda, Tanzania	No	6 weeks of classroom-based training, a 6-month practicum experience and mentoring.	LDP goal: To effect leadership behavior change among participants who would catalyze and support the long-term improvement of health institutions.<br><br>Study aim: To identify and describe evidence of individual health leadership behavior change among training participants during and shortly after the pilot year of the program.	The classroom-based training component was offered at African partner academic institutions and included the following weeklong modules: Leadership, Communication, Project Management, Health Information Systems, Monitoring and Evaluation, and Implementation Science. The practicum included projects in the four African host countries.	Yes	19 African and three US medical post-graduates, post-residency physicians and master's-level nurses were selected.	In the short term, fellows demonstrated increased leadership development during and shortly after the intervention. However, expanded interventions and/or additional time may be needed to support behavior change toward the maintenance stages.

6	Monroe-Wise, 2016	Afya Bora Fellowship	Botswana, Kenya, Uganda, Tanzania	No	1-year (Involves three classroom learning blocks each lasting three weeks, separated by two five month experiential attachment site rotations)	LDP goal: To provide future global health leaders with practical leadership and management skills that are not part of traditional health professional training.<br>Study aim: The purpose of this study was to assess what career changes, if any, the Afya Bora Fellowship's alumni have experienced since completing the fellowship, and to describe those changes.	Learning block 1: Leadership, Communication, Monitoring & Evaluation.<br><br>Learning block 2 Implementation Science, Health Informatics, Effective Grant Writing.<br><br>Learning block 3: Human Resources & Budget Management, Global Health Policy & Governance.<br><br>Attachment site 1: Attachment Site Rotations, Structured Mentorship, Online Modules, Responsible Conduct in Research, Research Methods.<br><br>Attachment site 2: Attachment Site Rotations, Structured Mentorship, Online Modules, HIV/AIDS as a Global Health Challenge, Project Management.	Yes	Between January 2011 and June 2013, 42 fellows from five countries participated in the Afya Bora Fellowship. This included 22 fellows in the pilot year (January–July 2011), and 20 fellows in the first full year of the program (June 2012-July 2013). Twenty-one fellows were nurses, and 21 were physicians. The fellows were employed in various health sectors.	Twenty-one (68 %) reported changes to their position at work; of those, sixteen (76 %) believed the change was due to participation in the fellowship. All alumni reported improved performance at work, and cited the application of a wide range of fellowship skills, including leadership, research, communication, and mentoring. Twenty-six (84 %) alumni spearheaded improvements in their workplaces and almost all (97 %) remained in contact with colleagues from the fellowship.
7	Nakanjako, 2015	Afya Bora Fellowship	Botswana, Kenya, Uganda, Tanzania	No	1 year (a total of 8 weeks of didactic lectures offered as three classroom learning blocks. Two 4.5-month long experiential trainings)	LDP goal: To respond to the increased need of innovative experiential leadership training for nurses and doctors in an African setting.<br><br>Study aim: To guide further development of in-service training opportunities to enhance leadership skills of nurses and doctors in Uganda and other developing countries.	The didactic modules were designed to equip fellows with skills in communication, leadership, monitoring and evaluation, implementation science, health informatics, research methods, grant writing, human resources, and budgeting, as well as global health policy and governance. The classroom-based modules employ case- based discussions in small groups and are draw on Africa focused case studies.<br><br>Two 4.5-month long experiential trainings at identified local governmental and non-governmental organizations involved in health-related activities including the Ministries of Health in the four African countries. The curriculum included four additional online modules, namely responsible conduct of research, research methods, project management, and HIV/AIDS updates.	Yes	Between January 2011 and January 2015, out of 51 applications,15 fellows (nine doctors and six nurses) participated in the program [Uganda].	New responsibilities assigned to fellow due to skills acquired during the fellowship.<br><br>Improvements in quality of work.<br><br>New projects/innovations/changes developed after the Afya Bora fellowship.<br><br>Dissemination conferences attended.<br><br>Other ways in which fellowship experience has improved health care delivery.
8	DeRusso, 2020	Leadership program	United States	Yes	10 months (9 didactic sessions that alternated between a half-day session (n = 5) and a full-day session (n = 4))	LDP goal: To strengthen the skills of emerging clinical leaders and to develop a pipeline of physician leaders.<br><br>Study aim: unclear.	The leadership program lasted 10 months and included several types of learning experience: individual assessments (eg, 360-degree evaluation), executive coaching, sharing of personal leadership experiences by physician leaders, description of the organization by hospital administrators, didactic sessions, experiential learning through a leadership project, career mentoring, readings, and up to 55 Continuing Medical Education (CME) credit hours (Table I).<br><br>The full-day sessions included topics on emotional intelligence, negotiation and conflict resolution, leadership in all life domains, and organizational culture. Half-day sessions included presentations by leaders on strategy development, organizational structure, enterprise initiatives, stakeholder analyses, identifying mentors, and experiential learning through leadership projects.	Yes	A total of 125 physicians have completed the program. Among these individuals, 52% were female, 60% were from the department of pediatrics, and 49% were associate professors. Many were medical directors leading hospital clinical programs or hospital units or were clinical directors within their department/division.<br><br>4 cohorts of participants in fiscal years 2016, 2017, 2018, and 2019.	Survey results indicated that all 125 participants from the 4 cohorts agreed their leadership skills were enhanced, they felt more connected to the institution, and they were committed to contributing to the enterprise-wide mission.<br><br>Participants used learnings from program sessions to create a vision, motivate others, resolve conflict, provide feedback, and improve communication.<br><br>Information from 93 participants in the first 3 cohorts showed that since completion of the program,

										53% were in a new leadership role at CHOP.
9	Ennis-Cole, 2019	Physician leadership development program	United States	Yes	6 months. Twice a month for two hours over six consecutive months (24 h of classroom instruction)	LDP goal: Strengthening the succession pipeline to increase the number of candidates who were ready for leadership roles.<br><br>Study aim: Explore the goals of physician leadership programs, models of leadership, cost reduction initiatives associated with physician leadership, information technology use, physician leadership program curricula, organizational challenges, and participant perceptions after an in-house leadership development program).	The content of the program was modeled after a previously implemented program that had been successful at the center. Activities in the physician leadership program included orientation, six months of training/exposure to curriculum content, monthly meetings with an Executive Team Leader, and mentoring and evaluation.<br><br>A different topic was presented each month of the program, and it was based on the center's Performance Standards. The topics were communication, development and learning, management and planning, relationship and team building, innovation and change, and patient centricity. For each topic there were learning goals, program, individual, and site metrics. There were two-hour classroom sessions with national speakers, a 60-min book club, and homework.<br><br>The program utilized a blended learning approach which included instructor-led classes, assigned readings, self-directed learning via a binder used as a resource guide, case studies, and online experiences.	Yes	10 physicians	Nine out of the 10 participants graduated from the program and moved into new or enhanced leadership positions. Participants reported that their capacity for collaboration increased and their new leadership skills were utilized in their new leadership roles.
10	Fassiotto, 2018	Stanford Leadership Development Program (SLDP)	United States	Yes	9 months (six one-and-one-half day sessions)	LDP goal: Focusing on basic leadership skills for Stanford Medicine faculty (somewhat unclear).<br><br>Study aim: To undertake a long-term, mixed-methods evaluation of an academic physician leadership program to understand impacts on both individual participants and on the organization.	The curriculum was comprised of competencies recommended for healthcare leaders including personal development as a leader, managing people and relationships, managing groups and projects, managerial finance and accounting, and understanding the organizational system. Sessions were taught using interactive teaching methods based on adult learning principles. During the program, participants led interdisciplinary teams to complete action learning projects related to their leadership role.	Yes	[Included in analysis]<br><br>131 participants, 82 (control-group).<br><br>2006 (n=23), 2007 (n=23), 2008 (n=11), 2009(n=22), 2010 (n=26), 2011 (n=26)	Program participants rated higher than non-participants across 25 of 30 items measuring leadership knowledge, skills, and attitudes, and were more likely to hold regional/national leadership titles and to have gained in leadership since program participation. Asian program participants were significantly more likely than Asian non-participants to have been promoted, and women participants were less likely to have left the institution than non-participants. Finally, qualitative interviews revealed the long-term impact of leadership learning and networking, as well as the enduring, sustained impact on the organization of projects undertaken during the program.
11	Hopkins, 2018	Stanford Leadership Development Program (SLDP)	United States	Yes	9 months (six one-and-one-half day sessions)	LDP goal: To strengthen implementation of quality, safety, patient-centered and cost effectiveness projects; create common physician behavior expectations; and improve participatory decision-making.	The curriculum was created based on competency models, institutional needs assessments, and recurring topics found in other programs.<br><br>Personal development as a leader: Assessments, Leadership styles.<br><br>Managing people and relationships: Performance management, Power, influence, and authority,	Yes	Between 2008 and 2011 (four cohorts), 113 individuals were enrolled. Nineteen of those enrolled in the program were high-level staff leaders in the school and hospital; the remaining	Reaction: The program was rated highly by participants (mean = 4.5 of 5). Learning: Significant improvements were reported in knowledge, skills, and attitudes surrounding leadership competencies. Behavior: The majority (80%–100%) of

						Study aim: To design a leadership program using established models for continuing medical education and to assess its impact on participants' knowledge, skills, attitudes, and performance.	Managing Conflict, Negotiation, Recruiting and developing talent, mentoring, Team dynamics, Diversity in appointments and promotions, Difficult conversations, Legal land mines in human resource management.<br><br>Managing groups and projects: Project planning and management, Organizational alignment, Change management, Meeting management, Managing teams.<br><br>Managerial finance and accounting: Financial statements, funds accounting, Revenue cycles, cost accounting budgets, Incentives, Business plans.<br><br>Understanding the system: Organizational Structure – school, hospital, Planning and decisions, Finances, Decision makers in the organization.<br><br>Each participant carried out an active learning project leading a multidisciplinary team.		94 were faculty members. Multiple medical disciplines.	participants reported plans to use learned leadership skills in their work. Improved team leadership behaviors were shown by increased engagement of project team members. Results: All participants completed a team project during the program, adding value to the institution.
12	Fernandez, 2016	The American College of Obstetrics and Gynecologists Robert C. Cefalo National Leadership Institute (ACOG NLI)	United States	No	3.5 days	LDP goal: To help ACOG leaders understand transformation and change in the context of understanding oneself and others; create effective teams and organizational cultures; understand the critical issues of cost-effective care and using epidemiological evidence to guide policy recommendations; learn skills to negotiate effectively; lead change; and become translators of complex scientific medical findings into simple, understandable language for the media as well as other physicians.<br><br>Study aim: To examine whether such a short-burst, intensive, experiential leadership development approach positively impacts physician's perceived skill level and application of a targeted set of leadership skills and whether participants perceived that the skills learned at the ACOG NLI remained strong even 6 months postprogram.	The ACOG NLI is comprised of a series of interactive skills-building workshops and includes completing a series of leadership and psychological assessment tools, including a 360-degree assessment. Participants meet with an executive coach to debrief their individual assessment findings, while small and larger group sessions teach boundary spanning leadership skills in organizational settings as a physician leader.<br><br>Skills Targeted in the ACOG NLI:<br>1. Creating collaborative organizational cultures<br>2. Leading others and empowering their success<br>3. "Selling" a change message<br>4. Leading change successfully<br>5. Motivating others at work<br>6. Applying advocacy skills using a science-based approach<br>7. Managing media communications<br>8. Negotiation skills<br>9. Women's health policy and high level leadership<br>10. Maximizing my personal leadership success while avoiding derailment	Yes	37 physicians who attended the eighth cohort (2013) of the ACOG NLI.<br><br>To date, 360 ACOG Fellows have completed the ACOG NLI through 10 annual cohorts of approximately 36 participants.	Course completion and 6-month postcourse scores indicated statistically significant improvements in scores on all 10 competency areas. Qualitative data gathered at the 6-month postcourse survey provide examples of how participants had applied their skills.
13	Ferris, 2018	The International Palliative Care Leadership Development Initiative (LDI)	United States	No	2 years (3 weeklong residential courses)	LDP goal: To expand the global network of palliative care leaders in low- and moderate-resource countries who are well positioned to apply their new leadership skills.	The two-year curriculum that included three thematic residential courses, mentorship, and site visits by senior global palliative care leaders and personal projects to apply their new leadership skills. Focus on self-reflection, leadership behaviors and practices, strategic planning, high-level communication, and teaching skills.	Yes	39 leaders representing 25 low-and moderate-resource countries	The leaders are using their new leadership skills to grow palliative care capacity through significant changes in policy, improved opioid/other medication availability, new and enhanced educational

						Study aim: describing the program (somewhat unclear).				curricula and continuing education activities, and development/expansion of palliative care programs in their organizations and regions.
14	Gholipour, 2018	The district health management fellowship training programme	Iran	No	9 months (11 courses over 24 days)	LDP goal: To build managerial capability in Iranian district health managers.<br><br>Study aim: To evaluate the district health management fellowship training programme in the north-west of Iran.	Educational courses:<br>1. Management, leadership (2 days)<br>2. Managing the district (2 days)<br>3. Quality improvement (3 days)<br>4. Planning and evaluation (3 days)<br>5. Health information management (2 days)<br>6. Health resources management and economics (3 days)<br>7. Community participation (2 days)<br>8. Epidemiology (2 days)<br>9. Research in health systems (3 days)<br>10. Human resources and organisational creativity (1day)<br>11. Rules and ethics (1 day)	Yes	72 participants (46 medical doctors), 2 cohorts. (2015-2016)	An improvement in knowledge of health system management. The courses on managing the district (51%), research in the health system (42%), and human resources and creativity (37%) had the most positive differences between pretest and post-test scores.
15	Hackworth, 2018	Core Leadership Program (CLP)	United States	Yes	10 months (4 days and 7 monthly 4-hour meetings)	LDP goal: To develop a cadre of outstanding faculty leaders across the career continuum, foster peer mentorship and coaching among participants, and encourage faculty leaders to create a culture of service, appreciation, inclusion, teamwork, and collaboration.<br><br>Study aim: describing the program (somewhat unclear).	The structure of the program included 68 hours of learning activities occurring over a 10-month time frame:<br><br>1. Two 1-day onsite seminars with experiential activities, presentations, videos, case studies, small group cohort discussions (exploration teams), learning triads, and 360° feedback on emotional intelligence.<br><br>2. A 2-day offsite session with experiential activities (eg, high ropes course), team-building activities to practice new leadership behaviors, “life stories” sharing, and personal strengths feedback.<br><br>3. 7 monthly 4-hour meetings with presentations on core skills by senior faculty leadership team members and exploration team breakout sessions to discuss leadership foundational skills and lessons learned from assigned readings and practice.<br><br>4. Reading assignments based on Discover Your True North and How Full is Your Bucket?<br><br>5. A half-day for “leadership profile” presentations by each participant.	Yes	A total of 99 faculty leaders participated in the first 4 cohorts of the program. The participants were multidisciplinary, including physicians, psychologists, biostatisticians, basic scientists, and others.	Significantly improved confidence, self-awareness, and strategic, operational, and relational skills. Qualitative data analysis demonstrated the positive impact of the CLP on the development of social networks, improved communication, and engagement.
16	Howell, 2019	Pathology Leadership Academy	United States	No	1.5 days year one, and 1 day year two	LDP goal: […] aid in development of faculty leaders for<br>departments of pathology and laboratory medicine, increase the diversity of faculty within the specialty, and enhance the functionality of diverse teams.<br><br>Study aim: The purpose of this report is to: (1) describe the PLA’s development, curriculum, and evaluations from its first 2 years; (2) illustrate how the	Drexel University’s Executive Leadership Program in Academic Medicine (ELAM) served as a major inspiration for the PLA.<br><br>The PLA curriculum for both years also reflects topics identified in the 2014 chairs’ survey and included speakers external to the APC, in addition to speakers who were active and former chairs and members of APC. Pathology Leadership Academy provided lunch programs with networking opportunities and informal table discussions with senior fellows (previous chairs of pathology departments) and active chairs.	Yes	In the first year of the PLA, there were 58 participants from 43 departments in 19 different states.<br><br>In the second year of the program, there were 37 participants from 22 departments in 17 different states.	Chairs reported that Pathology Leadership Academy provided value to their faculty through preparation for a future leadership role, enhancing skills for a current role, and enhancing understanding of opportunities and challenges in academic medicine.

						program has met leadership development needs of academic pathology departments and individual faculty participants; and (3) share how experiences to date could be used to improve the PLA, as well as benefit other leadership programs.	Topics curriculum year 2: Leadership styles, Finding strengths as leader, Pathways to leadership, Negotiating for wat you need.			
17	Leggat, 2016	Clinical Leadership in Quality and Safety Course	Australia	No	1 year (somewhat unclear)	LDP goal: To educate current and potential clinical leaders in the knowledge, skills and competencies required to successfully lead the healthcare quality and safety agenda, incorporating both an organisational and system-wide perspective.<br><br>Study aim: To develop clinical leadership among health professionals working in public sector organisations to improve their skills in ensuring high quality and safe health services.	The curriculum comprised a quality and safety simulation and a workplace project, which were supported by online materials, face-to-face workshops and regular communication with other participants and the program faculty.	No	The program was targeted to clinicians, and of the 62 total participants, 15 (24%) clinicians reported a management role. All health professions were represented with seven (11%) medical, 22 (35%) allied health and 33 (54%) nursing participants. (2011/12 and 2013).	Significant improvements in the leadership practices inventory, emotional intelligence, psychological empowerment, and patient safety skill score. Patient safety attitudes did not change significantly. Qualitative results showed strengthened quality and safety knowledge and skills, and developments in organizational and systems literacy. Participants reported that many projects had resulted in distinct and recognizable changes to practice.
18	Levine, 2015	Johns Hopkins University School of Medicine Leadership Program for Women Faculty (LPWF)	United States	Yes	10 months (9 half-day interactive session)	LDP goal: The LPWF was developed to enhance leadership skills and networking opportunities for women faculty.<br><br>Study aim: To present a description and evaluation of a longitudinal, cohort-based, experiential leadership program for women faculty at the Johns Hopkins University School of Medicine.	The LPWF focuses on specific gender and leadership content areas as well as the institutional culture and uses both to accomplish curricular goals.<br><br>The LPWF includes 8 modules (Table 1). Each session begins with unstructured time for participants to network. The LPWF includes opening and closing events that highlight current women leaders in the SOM as guest speakers. The modules are designed to promote experiential learning by incorporating interactive case studies, facilitated discussion, skills building using role-play, and reflective practice.<br><br>Modules:<br>1. Working Together:<br>2. Crucial Conversations<br>3. Speak Like a Pro<br>4. Influencing for Impact:<br>5. Creating Agreement and Managing Conflict<br>6. Leadership Challenges for Women: Overview of Decision-Making and Risk-Taking Strategies<br>7. Leadership Challenges for Women: Facilitating Group Decision-Making<br>8. Total Leadership	Yes	Since the inception of the LPWF in 2009, 174 women have participated in the LPWF.<br><br>Cohort 1 (2009-2010) n=40<br>Cohort 2 (2011-2012) n=45<br>Cohort 3 (2011-2012) n=50<br>Cohort 4 (2012-2013) n=39<br><br>Multiple medical disciplines	Significant improvements in skills across 11 leadership domains with the exceptions of 2 domains, Public Speaking and Working in Teams The greatest increase in rankings occurred within the domain of Negotiation Skills. Qualitative results identified two major themes: (1) Networking and reflecting with other women is valuable, and (2) Negotiation skills are important and can be developed.
19	Lewis, 2021	The Hedwig von Ameringen Executive Leadership in Academic Medicine program (ELAM)	United States	No	1 year	LDP goal: to help address ongoing underrepresentation of women among the ranks of senior leaders in academic medicine.<br><br>Study aim: To explore the experience of ELAM Fellows and leaders from one institution to elucidate how institutional	ELAM offers intensive leadership training, coaching and mentoring. A core component of the curriculum is the Institutional Action Project (IAP), through which fellows incorporate and translate curricular objectives into tangible outcomes. [focus of the article].	Yes	11 faculty from the institution who enrolled in ELAM between 1999 and 2017, 6 physicians.	Project work had bidirectional impact fellows and the institution. Leading indicators of institutional outcomes included contributions to institutional leadership and culture, and mutual enhancement of the reputation of the fellow and of the institution.

						factors influence project implementation and outcomes.				
20	Macphail, 2014	Clinical leadership program (CLP)	Australia	Yes	9 to 10 months (one 2-hour session on-site once per month)	LDP goal: To foster leadership capability and encourage engagement of staff in decision-making within their team and department.<br><br>Study aim: To appraise whether an in-house CLP is feasible and effective.	The framework for appraisal considered three aspects of the CLP, leadership development, multidisciplinary teamwork and leadership learning. The key elements of the CLP programme were: one 2-hour session on-site once per month for nine to ten months (equivalent to 20 hours), with a guest speaker and group discussion; one self-organised external site visit and one mini-project, both completed in small, interdisciplinary groups; and a presentation to peers and executive staff of their learning from the site visit and the mini-project. Nine on-site sessions were completed in the pilot programme.	2011 Yes<br>2012 No	In 2011, 17 participants, 3 medical. In 2012, 22 participants, 3 medical.	The CLP significantly increased willingness to take on leadership roles. Five of the 11 participants from the 2011 programme had taken on a new leadership role 18 months later. Senior executive feedback was positive especially around the engagement and building of staff confidence.
21	Maddalena, 2015	Physician Management and Leadership Program (PMLP)	Canada	No	1 year (10 modules)	LDP goal: To provide current or aspiring physician leaders with an introduction to the kinds of skills they will need to function within the health-care administrative environment.<br><br>Study aim: To document the process the province of Newfoundland and Labrador used to develop an innovative Physician Management and Leadership Program (PMLP).	Of the PMLP's ten modules, three are delivered online (asynchronous) and seven are delivered in a classroom setting. In-class modules range in length from 4 to 7 hours:<br>1. A Self-Discovery Approach to Leadership<br>2. Strategic Planning<br>3. Managing Competing Priorities<br>4. Project & Change Management<br>5. Organizational Structures<br>6. Evaluation Methods<br>7. Public Relations & Communications<br>8. Patient Safety<br>9. Recruiting & Performance Development<br>10. Leading vs. Managing	Unclear	In the 2012-2013 PMLP Pilot (n=35 physicians; n=2 non-physicians). In the 2013-2014 PMLP (n=33 physicians; n=1 non-physician).	Significant pre-/post-increases in knowledge and confidence. 95.07% reported feeling more prepared for their leadership responsibilities. 95% also agreed that it enhanced their interest in a leadership position. Data demonstrates application of the knowledge/skills obtained from PMLP in the workplace.
22	McCray, 2018	postgraduate medical leadership development programme	United Kingdom	Yes	1 year, monthly meetings	LDP goal: It was hoped that this group of people would be the first to begin to initiate a change in the organizational culture, which would impact positively on performance, establish new networks for collaboration and the integration of services, and improve the quality and resilience of care delivery needed at organizational, team and individual level.<br><br>Study aim: To explore the influence of one cycle of a learning set experience in a postgraduate medical leadership development programme.<br><br>[action learning (AL) and critical action learning (CAL) perspective]	The programme was held on one day a month for one year and comprised three events: (1) a reflective commentary and discussion led by the Trust's chief executive; (2) learning sets of five to six people; (3) lecturer-led input to support a work-based project centred on integration of services that formed a key area for learning set discussion on progress.	Unclear	Participants are nine members from an 11-member senior medical doctor cohort enrolled in a postgraduate leadership programme; [Study participants].	The paper affirms other study findings that CAL in the development of participants' collective reflexivity has the potential to deal with emotions and power relations in organizational life. An original contribution lies in advancing the idea that CAL can help build resilience in doctor leaders and groups in uncertain conditions such that they are able to challenge current care delivery and effect change in organizational performance.
23	Miani, 2013	Barking, Havering and Redbridge University Hospitals NHS Trust Fellowships in Clinical Leadership Programme	United Kingdom	Yes	1 year	LDP goal: To develop (or strengthen) individual leadership skills while learning about change implementation and organisational management.<br><br>Study aim: To better understand the impact of the Fellowships in Clinical Leadership Programme,	The Fellowships in Clinical Leadership Programme (the Programme), introduced in March 2012 for a period of 12 months, involved the appointment of clinicians (doctors, nurses, midwives) on a one-year contract at the Trust to lead on a range of diverse quality improvement (QI) projects. The combination of learning activities, clinical duties and QI project work sought to enable participants to transfer and manifest new competencies in their QI projects, and improve the quality of care within the Trust.	Yes	Two schemes, with Scheme A (10 external Fellows, 20 senior clinicians) involving clinicians from a variety of specialties while Scheme B (4 external Fellows, 8 internal Fellows, 6 neonatal nurses, 12 senior	The Programme had notable impacts at individual and organizational levels. Individual impact included enhanced communication and negotiation skills or increased confidence. At the organizational level, participants reported indications of behavior

						both on individual and group behaviours as well as on service quality improvement or organisational development, NHS London commissioned RAND Europe, in collaboration with Improvement Science London, to conduct an evaluation of the Programme.			clinicians) focused on maternity services in particular and involved mainly midwives and nurses.	change among staff, with evidence of spill-over effects to non-participants towards a greater focus on patient-centered care.
24	O'Neil, 2019	Applied Physician Leadership Academy™ (APLA)	United States	Yes	18 months, 8 modules	LDP goal: To grow the talent of its existing leaders to manage change alongside THS's health system executives.<br><br>Study aim: To illustrate the incorporation of an evidence-based management approach in support of evidence-based organizational development practice.	The academy utilizes multiple learning strategies including learning and application modules, 1:1 assessment and coaching, and Action Learning teams and projects to foster rapid leadership development. The APLA's multi-modality approach is designed to build physician engagement and strengthen physician leadership capability.<br><br>Leading Self, Leading Others, Leading Change, Leading for Results	Unclear	Unclear<br><br>Twenty-six emerging physician leaders joined together from across the organization to kick off a second cohort of the APLA in January 2017.	Post-APLA results demonstrated an increase in the frequency with which the participants reported they exhibit these competencies. Leadership behavior change (approaching leadership tasks, giving feedback, importance attributed to management role).
25	Pradarelli, 2016	Leadership Development Program	United States	Yes	8 months (1 full day per month)	LDP goal: To train surgical faculty to enhance their leadership abilities.<br><br>Study aim: To evaluate critically a Leadership Development Program for practicing surgeons by exploring how the program's strengths and weaknesses affected the surgeons' development as physician-leaders.	Surgeon-participants gathered in-person for 1 full day per month for 8 consecutive months for didactic and experiential learning. Examples of curricular content that was surgeon-specific included surgery-oriented business case studies (eg, Harvard Business School case on building an ambulatory surgery center) and finance sessions, where departmental and division administrators shared financial statements from the Department of Surgery. Participants also conducted longitudinal team projects that focused on improving clinical care, education, or research processes within the Department of Surgery. Each participant underwent baseline, 360-degree evaluations by colleagues and supervisors and by direct reports regarding their leadership performance.<br><br>Four domains: Leadership, Team-building, Business acumen/finance, Health care context.	Yes	21 surgical faculty members (2012).	surgeons reported personal improvements in the following 4 areas: self-empowerment to lead, self-awareness, team-building skills, and knowledge in business and leadership. Surgeons felt "more confident about stepping up as a leader" and more aware of "how others view me and my interactions." They described a stronger grasp on "giving feedback" as well as a better understanding of "business/organizational issues." Overall, surgeon-participants reported positive impacts of the program on their day-to-day work activities and general career perspective as well as on their long-term career development plans.
26	Rao, 2017	Partners Clinical Process Improvement Leadership Program	United States	Yes	4 months (3 sessions ranging from 1 to 2 days each followed by one final day of project presentations.)	LDP goal: to develop a longitudinal, local, interdisciplinary, team-based QI educational program for active clinicians and administrators to align with institutional priorities in QI and engage frontline clinical teams.<br><br>Study aim: This article describes the Partners Clinical Process Improvement Leadership Program (CPIP).	The Partners Clinical Process Improvement Leadership Program (CPIP) is a 6-day experiential program. Interdisciplinary teams complete a QI project framed by didactic sessions, interactive exercises, case-based problem sessions, and a final presentation.<br><br>Besides project work, session topics included: History of QI, Change concepts, Using SCP in Healthcare.	Yes	From March 2010 to July 2015, 13 cohorts consisting of a total of 239 teams and 516 individuals participated in CPIP. Forty-five percent (233/516) are practicing physicians; 35% (179/516) are registered nurses/other clinicians; and 20% (104/516) are administrators.	A total of 239 teams composed of 516 individuals have graduated CPIP. On completion, participant satisfaction scores average 4.52 (scale 1-5) and self-reported understanding of QI concepts improved. At 6 months after graduation, 66% of survey respondents reported sustained QI activity.
27	Rask, 2011	P1. Leadership for Healthcare Improvement Course	United States	Yes	P1. 2 days<br>P2. 4-months, 12 days	LDP1 goal: To equip leaders and supervisors with a basic understanding of QI concepts and vocabulary, along with a	P1. Lectures and small group discussion.<br>The Case for Quality: Current state of health care, Current state of Emory's health care system, Emory's	P1. Yes<br>P2. No	P1. 545 leaders participated, 44 (8.1%) of the participants were physicians, and 136	P1 participants significantly improved knowledge in all content areas, and self-assessments revealed high

		P2. Practical Methods for Healthcare Improvement Course				clearly articulated vision of the quality culture that EHC seeks to engender. The expectation is that leaders completing the course will be able to actively promote the desired attributes of culture and will engage, enable, and sustain frontline staff in their QI activities.<br><br>LDP2 goal: To develop frontline employees capable of leading quality initiatives and department managers capable of managing improvement processes in their local clinical service areas.<br><br>Study aim: We describe the design and evolution of a unique two-pronged, internally delivered QI training program that targeted both health system leaders and frontline staff around a central mission to improve health system performance.	strategy and structure of the Office of Quality, Public reporting of quality data and public accountability.<br><br>Processes Have Behaviors: Distribution and variation.<br><br>Health Outcomes Result from Processes: Human factors and reliability science, Health care processes, Quality and cost.<br><br>Tools for Process Management: Improvement techniques.<br><br>P2 Didactic, group discussion, group exercises, case studies, role play, experiential, small group breakout sessions, panel discussion.<br><br>Content examples: Contributing factors and important roles in highly functioning teams, barriers to team function, negotiating areas of conflict, history of modern PI, examples of successful PI practice in our system, introduction to Lean, introduction to 5S, 5S exercise, experiences doing test of change, examples of PI.<br><br>Project presentations, the context for improvement, health information technology, data warehouse, culture transformation, leading change, sustainment and spread.		(25.0%) had a nursing background, multi-disciplinary. (9x, 2008 and 2009).<br><br>P2. 85 participants of whom 16 (18.8%) were physicians and 18 (21.2%) were nurses. Multiple medical disciplines. (4x, 2008-2009).	comfort levels with QI principles following the training.<br><br>All P2 participants were able to initiate and implement QI projects. Participants described significant challenges with team functionality, but a majority of the QI projects made progress toward achieving their aim statement goals. A review of completed projects shows that a significant number were sustained up to one year after program completion.
28	Sanfey, 2011	Leadership in Academic Medicine (LAM)	United States	Yes	10 weeks (10 3 to 7-hour sessions)	LDP goal: To further develop faculty members who had demonstrated leadership potential within the School of Medicine.<br><br>Study aim: The purpose of this study is to elicit faculty perspectives on their leadership skills before and after participation in a Leadership in Academic Medicine (LAM) Program.	The LAM curriculum is based on the women-only Executive Leadership in Academic Medicine program at Drexel University, which focuses on departmental and institutional fiscal planning, emerging issues, and personal and professional development. Transformational leadership and emotionally competent is a priority of the curriculum. All participants engage in extensive self-analysis using standardized tests such as the Myers Briggs personality test and the Leadership Skills Inventory 360◦. The instructional sessions focus on key professional development areas, such as managing organizational change, making strategic decisions, assessing the dynamics of successful leaders, financial management, and finding life balance in a growing career.	Yes	18 of the 32 participants were male (56%), and 22 (69%) held an M.D. degree. (2008).<br><br>Post-survey to 110 prior participants (2004-2007).	All participants reported improved leadership skills, but the percentages were lower for Long-Term Post-LAM participants than for the Immediate Post-LAM participants. In addition, although 58% of Immediate Post-LAM women, compared with 19% of Immediate Post-LAM men ($p < .05$), were actively seeking leadership roles, this was reversed in the long-term group (26% of women vs. 66% of men; $p < .05$).
29	Shah, 2013	Sailing a Safe Ship" (SASS)	United Kingdom	Yes	2 days (8 sessions)	LDP goal: to facilitate ophthalmologists' understanding<br>and analysis of their personal learning needs in relation<br>to the task ahead, and so be more proactive in accelerating their transition period.<br><br>Study aim: to describe a 2-day program focused on new and prospective consultant specialist ophthalmic surgeons entitled "Sailing a Safe Ship" (SASS).	The program used gaming, team challenges, meta-planning, role play and professional actors, interactive presentations, and self-analysis tools.<br><br>Sessions:<br>1. Role-playing session using case scenarios of challenging clinical and managerial situations.<br>2. Focusing on leadership and the power of the team<br>3. Risk management strategies<br>4. It's all about communication<br>5. Repeat role-play sessions.<br>6. How the consultants can maximise support and collaboration within the wider team<br>7. Understanding the General Medical Council and National Clinical Assessment Service agenda<br>8. Summary and close	Unclear	Unclear, consultant specialist ophthalmic surgeons.	Participants' insights reflected 4 key themes: admitting vulnerability and uncertainty, taking responsibility for managing risk, being self-aware and reflexive, and internalizing authentic leadership.

30	Smith, 2014	Faculty Leadership Academy (FLA)	United States	Yes	2 years (quarterly seminars)	LDP goal: To assist physicians in successfully navigating the difficult complexities facing healthcare today.<br><br>Study aim: The purpose of this study was to describe and analyze the perspectives of physicians who attended the Faculty Leadership Academy (FLA) to determine their perspectives on program effectiveness in developing leadership competencies and skills, successful instructional strategies and the impact of the FLA on their intentions to pursue leadership opportunities.	The academy is a two year program where participants attend quarterly seminars focused on skill development of competencies required to face critical issues in healthcare. Academy I modules are Negotiation Skills and Principles, Quality Improvement Imperative, Drivers of Financial Performance, and Problem Solving Using Analytic Tools. Academy II modules are Clinical Capital Investing, Managing Disruptive Behavior, Towards a Higher Standard of Patient Safety, and Mediating Medical Staff Conflict.	Yes	Approximately 30 members are enrolled into the FLA program each year. A total of four cohorts and 113 graduates have completed the coursework.<br><br>A total of 11 physicians were interviewed.	Participants indicated finding the course effective, especially the negotiations and finance modules. These modules provided new language, a better understanding of processes and an opportunity to develop skills through interactive class exercises such as case studies. Participants described an increased self-awareness of their interpersonal skills and expressed a desire for greater exposure to emotional intelligence principles. Participants experienced a transformational shift in how they constructed their identity as a physicians and leaders, and questioned assumptions about the physician's role in healthcare.
31	Smith, 2019	The Radiology Leadership Academy (RLA)	United States	Yes	9 months (one full day per month)	LDP goal: To develop highly engaged leaders who offer innovative solutions to adaptive challenges within the department, institution, and community.<br><br>Study aim: To share our experience in developing and implementing an interprofessional leadership development program within a large academic radiology department.	Principles central to the curriculum design were (1) broadening awareness and understanding of our department's multifaceted role within the academic health center and (2) building key leadership competencies. One important design consideration was cohort composition. We chose to include both faculty and non-faculty in each cohort to offer the best opportunity for shared awareness, meaningful learning, and positive organizational outcomes. With a core curriculum as the program foundation, individual course modules may be revised annually based on participant feedback regarding relevance to and impact on the learning experience, current environment or industry shifts, and organizational needs.<br><br>Key competency areas:<br>1. Leadership theory and concepts<br>2. Self-awareness and management<br>3. Leading teams and developing others<br>4. Leading an organization<br>5. Business concepts<br>6. Communication skills	Yes	109 participants (physicians, nurses, administrators), 9 cohorts (2009-2010, 2010-2011, 2011-2012, 2012-2013, 2014-2015, 2015-2016, 2016-2017, 2017-2018).<br><br>75 participants from cohort 1/8 participated in study.	Over the past decade, 100 participants have completed the program with 80% retention, substantial professional growth, and increased capacity for mentoring others.<br><br>RLA team projects emphasized four areas: (1) improving the patient or customer (eg, referring physician) experience, (2) improving the employee experience, (3) business process improvement, or (4) improved safety or quality of images or services. Nearly all of the team projects that were developed and piloted within the RLA have been partially or fully implemented in the department.
32	Steele, 2020,	Association of American Medical Colleges (AAMC) Council of Deans (COD) Fellowship Program	United States	No	1 year	LDP goal: Support medical school deans in their role (somewhat unclear).<br><br>Study aim: The purpose of this study was to determine the outcomes of the COD Fellowship Program with respect to participants' achieving the goals of becoming a medical school dean and developing leadership skills.	The yearlong program has 4 main components: (1) dean mentoring; (2) an introduction to the strategic goals and activities of the COD through attendance at COD meetings; (3) completion of a leadership project that addresses an issue of importance to the fellow's institution; and (4) attendance at the AAMC Executive Development Seminar for Deans, an executive leadership development program for new deans.21	Yes	Of the 37 COD fellows invited to participate in the survey, 27 (73%) responded. Multiple medical disciplines.	The web-based search found that 27% (10/37) of the fellows became medical school deans (average tenure 5.6 years); 2 fellows became deans of other types of schools. The majority (88%, 23/26) indicated their fellow experience persuaded them to pursue being a dean; 2 (8%) indicated it did not.

33	Throgmorton, 2015	Physician Leadership Academy (PLA)	United States	Yes	9 month (10 sessions)	LDP goal: To strengthen leadership skills and help physicians thrive in the ever-changing, fast paced world of healthcare.<br><br>Study aim: To outline the evaluation strategy and outcomes of the inaugural year of a Physician Leadership Academy (PLA) developed and implemented at a Michigan-based regional healthcare system.	The organizational needs assessment and best practice research resulted in a program that sequenced topics and experiences to build self-awareness/intra-personal, interpersonal, and organizational effectiveness.<br><br>Topics:<br>1. Introduction<br>2. Intra & Interpersonal Effectiveness<br>3. Personal Resiliency<br>4. Coaching<br>5. Communication<br>6. Teamwork<br>7. Change Management<br>8. Business Acumen<br>9. Quality Focus<br>10. Wrap Up Session	Unclear	21 physicians (2021 chohort). Multiple medical disciplines.	Four themes emerged from the interview data: increasing self-awareness; building relationships; applying new skills; and building confidence.<br><br>Completions of the program activities outlined in Level 2 results were also indicative of behavior change and engagement.<br><br>Organization based results were evaluated via survey from a stakeholder audience to whom PLA participants presented their team based project work, 97 percent agreed the work of the teams illustrated alignment with the organization's stated strategies.
34	Toma, 2020	Scottish Quality and Safety Fellowship (SQSF)	United Kingdom	No	10 months	LDP goal: To develop clinicians with advanced quality improvement knowledge, technical ability and essential leadership skills.<br><br>Study aim: The evaluation explores four levels of educational and practice outcomes associated with (1) the reaction of fellows to SQSF participation, (2) learning gained, (3) subsequent behaviour changes and (4) the overall impact on national and international level capability and capacity building.	The educational programme content consists of interactive learning sessions focusing on developing leadership skills and promoting QI principles and values, as well as residential workshops to facilitate learning from global experts, and practical opportunities to apply and spread the learning in the workplace setting and beyond.<br><br>Main topics:<br>1. Understanding of Quality Improvement Methodology<br>2. Demonstrate ability to design and deliver a QI project<br>3. Deepen understanding of Leadership skills<br>4. Develop networking capability	Unclear	Of the 222 participants in cohorts 1 to 10), the majority were Scottish based (n=143, 64.0%), female (n=129, 58.1%) and medical doctors (n=138, 62.0%).	Most participants reported improved social, behavioural and emotional skills, knowledge and attitudes and, with sustained support of their host organisations, were able to apply and share learning in their workplace. The impact of the SQSF on a wider national and international level capability and capacity was both mediated and moderated by a widerange of interrelated contextual factors.
35	Torbeck, 2018	Leaders Growing Leaders	United States	Yes	Program with 4 tiers (T)<br><br>T1. During 2014 to 2016 (7-modules, once every 3 months)<br><br>T2. 1 year (monthly)<br><br>T3. 1 year (monthly)<br><br>T4. 3 to 6 months	LDP goal: To cultivate an interest in leadership education and to enhance faculty members' capabilities as leaders, especially as it pertains to their administrative skills.<br><br>Study aim: The purpose of this paper is to describe the structure of a tier-based leadership development program called Leaders Growing Leaders, to identify the major curricular components to each tier including measures and outcomes, and to share lessons learned for those who may want to begin a similar leadership development program.	T1: (1) Introduction to leadership: the art of changing the conversation, (2) strategic planning, (3) negotiation and conflict management, (4) strategic marketing of health care, (5) creating change, (6) creating and communicating a compelling vision, and (7) managing difficult people (especially junior faculty).<br><br>T2. Action learning. (1) What makes a great leader?, (2), Goal setting: spending time in quadrant II, (3) Culture and office politics, (4) The role of trust and accountability, (5) Managing your time and managing failure (junior faculty who had leadership roles).<br><br>T3. Action learning. (1) Principles of decision-making, (2) The role of emotional intelligence, (3) Evaluating performance, (4) Preparing a program budget, (5) The role of trust and accountability, (6) Crucial conversations, (7) The science and art of recruitment, (8) Changing the culture, (9) Managing time, (10)	T1. No<br><br>T2. Yes<br><br>T3. Yes<br><br>T4. Yes	T1. Average attendance was ~20 to 25 faculty (20% of faculty) at each session; 57 out of 121 faculty attended at least 1 session<br><br>T2. 6 faculty<br><br>T3. 9 faculty<br><br>T4. 5 senior leaders	The "deliverables" from the Tier II and III leadership sessions were very insightful and indicated being impactful on both the learning and behavior outcome levels.<br><br>Additionally, in the last 5 years, 5 of the 6 division chiefs have turned over and all have been replaced by internal candidates whom we have groomed through various leadership skill building programs.

							Managing conflict (faculty who had demonstrated leadership success in junior roles).<br><br>T4. Strategies for development included 360° leadership evaluation and feedback, executive coaching for 3 to 6 months with targeting of issues identified in the 360° evaluation, and monthly coaching sessions with the chair. (senior leadership roles).			
36	Tsoh, 2019	UCSF-Coro Faculty Leadership Collaborative (FLC)	United States	Yes	20 weeks (10 sessions)	LDP goal: To catalyze individual and collective change to benefit both the participants and the broader UCSF community.<br><br>Study aim: To describe perspectives from our 12-year experience cultivating a formal faculty LDP within an academic health center and longitudinal outcomes of our LDP.	Coro's design of curriculum and learning activities encourages participants to accept new tools introduced in the training, apply the tools, and to then adapt the tools, as needed, based on one's experiences and relevancy after a process of reflection. Coro uses an array of tools to focus on developing six core leadership competencies: 1) self-awareness, 2) critical thinking, 3) effective communication, 4) inclusion, 5) collaboration, and 6) empowered professionalism (confidence to represent oneself consistently in many different environments), rather than following a specific leadership model.	Yes	137 participants: 108 Medicine, 14 Dentistry, 8 Nursing, 6 Pharmacy (7 cohorts, 2005-2012)	The proportions of graduates attaining leadership positions within UCSF such as deans or department chairs among all, URM, and women URM graduates were 9.6%, 33.3% and 45.5%, respectively. Graduates' perceived impacts from 8 months to 8 years after program completion and showed 91.7% of survey respondents felt the program both increased their understanding of UCSF as an organization and demonstrated the University's commitment to foster faculty development. Qualitative results indicated that graduates perceived benefits at individual, interpersonal, and organizational levels.
37	Vitous, 2019	Leadership development program (LDP)	United states	Yes	Unclear, 1 year cohort	LDP goal: Unclear<br><br>Study aim: To explore the impacts of implementing a leadership development program on the culture of the Department of Surgery at University of Michigan, Ann Arbor.	The program was structured around four domains: leadership, team building, business acumen, and health care context.	Yes	A total of 21 surgeons participated in the program: 15 men and 6 women.(2012-2013). Multiple surgical clinical areas.	The program influenced surgical culture in the following ways: (1) promoted a more participative leadership style, providing tools for surgeons to create a more collaborative environment; (2) increased the culture of diversity, with leaders in the department valuing a more inclusive and wide range of skill sets; and (3) strengthened the collegial environment as evidenced by improved morale and relationships within the department.
38	Vreeling, 2019	Mindful leadership for medical specialists	Netherlands	No	10-weekly 5-hour sessions	LDP goal: To become a better leader (unclear)<br><br>Study aim: To explore how a 'Mindful leadership for medical specialists' course affected medical specialists' leadership.	The course included the original Mindfulness-Based Stress Reduction (MBSR) programme developed by Jon Kabat-Zinn. In addition to the MBSR, the course consisted of experiential teaching sessions on cognitive behavioural therapy, compassion and three frameworks relevant to becoming a better leader.	No	In total, five cohorts attended the mindful leadership course, totalling 52 medical specialists overall. Multiple medical disciplines. Seventeen invitees agreed to participate	Themes were defined within these categories: awareness of self, open mind, insight and appreciation of self (A), emotional and cognitive self-regulation, letting go of unhelpful behavior and developing helpful behaviour (B), differences in attitude to others such as awareness of (impact on) others, keeping an open mind about others,

										allowing difficult emotions associated with others, appreciating of others (C), and communicating more effectively, providing direction, empowering and caring for others (D).
