# Supplementary material for: Linking leadership development programs for physicians with organization-level outcomes: a realist review
Source: BMC Health Serv Res. 2023 Jul 21;23:783. doi: 10.1186/s12913-023-09811-y (PMC10362722; doi:10.1186/s12913-023-09811-y)
Supplement: Supplementary file 4 — Additional file 4: Supplementary material D. Evidence backing up MRPT. Note: we do not exhaustively give all possible available fragments from studies that might support our MRPT. Note: we included studies in this table if they included information on the C and M, or C and O, or M and O, of the proposed CMO. [file 12913_2023_9811_MOESM4_ESM.pdf]

Supplementary material D – Evidence backing up MRPT

Note: we do not exhaustively give all possible available fragments from studies that might support our MRPT

Note: we included studies in this table if they included information on the C and M, or C and O, or M and O, of the proposed CMO.

Acquiring self-insight and people skills (CMO1)<br><br>If LDPs include constructive feedback on physicians' personality traits and leadership behavior [C], physicians become more self-aware and obtain insight into the needs and preferences of the people they lead. Accordingly, they adopt a people-oriented leadership style which benefits communication and collaboration [M], and thereby the organization's culture [O].	
Studies that provide (partial) evidence	DeRusso, 2020; Ennis-Cole, 2019; Fernandez, 2016; Ferris, 2018; Hackworth, 2018; Hopkins, 2018; McCray, 2018; Miani, 2013; O'Neil, 2019; Pradarelli, 2016; Sanfey, 2011; Shah, 2013; Smith, 2014; Throgmorton, 2015; Toma, 2020; Tsoh, 2019; Vitous, 2019; Vreeling, 2019.
Illustrative supporting evidence (text fragments, descriptions, data)	Individual assessments and the one-on-one coaching were essential attributes of the program. The 360-degree evaluation, the dominance, influence, steadiness, and conscientiousness (DiSC) behavior assessment tool, and the emotional intelligence assessment provided feedback that created an opportunity for change in behavior. Coaches assisted in interpreting the feedback and developing an action plan. [C/M] - DeRusso, 2020<br><br>Participants used learnings from program sessions to create a vision, motivate others, resolve conflict, provide feedback, and improve communication. [C/M] - DeRusso, 2020<br><br>The physicians who accepted the invitation received two self-assessments: an in-house 360-degree assessment and an insights profile. [C] - Ennis-Cole, 2019<br><br>Participants stated that they started to develop team-building skills, work toward consensus, and build others' trust prior to starting the physician leadership development program. Their participation in the program only enhanced these skills. [M] - Ennis-Cole, 2019<br><br>Through coaching and learning, this same participant realized that he needed to be deliberate about using these same skills 24/7 and transferring them outside of the clinic when he interacted with nurses and administrators. He learned that this would make him a more effective leader. [C/M] - Ennis-Cole, 2019<br><br>The ACOG NLI is comprised of a series of interactive skills-building workshops and includes completing a series of leadership and psychological assessment tools, including a 360-degree assessment. Participants (hereafter referred to as Fellows) meet with an executive coach to debrief their individual assessment findings. [C] - Fernandez, 2016<br><br>Twenty-three of the 26 Fellows (88.5%) completing the 6-month follow-up survey provided qualitative data, which provided examples of how skills from the course were utilized. Eighty-three percent of respondents cited improved communications skills, and 91% cited creating a team-based culture. [M/O] - Fernandez, 2016<br><br>"This course definitely was a life-changing experience. I have used many of the skills I learned to organize/run meetings, communicate more effectively with

	people, and seek out opportunities that fit my style better." [M] - Fernandez, 2016<br><br>Participants indicated that the mentorship and coach interactions were an important part of their learning experience. They helped me, the mentors with leadership in palliative care. Able to ask for guidance. The coach help me understand myself better and get emotionally stronger. It was a great experience for me especially since my mentor taught me how to analyze or assess my perceptions/feeling or attitude towards issues or people and make unbiased decisions about different issues. This has greatly improved my ability to work with a team and value every member. I think that the interaction with the mentors and the coaches is one of the most important aspects of the RC. In the lobbies, during breaks and lunches, there is a parallel curriculum that is as interesting as the programmed course. I learned a lot through the advice and conversations I had with my mentor and the rest of the LDI team. [C/M] - Ferris, 2018<br><br>The program was designed to (1) provide opportunities for self-reflection, peer support, and practice over an extended period; (2) use multiple learning formats, including onsite and offsite learning settings, 360° feedback, small groups, experiential learning, and guest speakers, to maintain energy and excitement; (3) initially focus on helping participants discover their personal strengths, limitations, and values before focusing on leadership competencies and core skills; (4) connect to participants' experiences with leadership at work as well as in their homes and communities; and (5) help participants acknowledge how personal viewpoints can positively or negatively impact their leadership influence. [C] - Hackworth, 2018<br><br>Participants also reported that the program increased their awareness of the impact of their leadership behaviors on others, increasing their utilization of positive leadership styles and behaviors; increased their ability to engage and empower members of their clinical/research teams; increased self-confidence in their leadership skills and better pre- pared them for future leadership roles; enhanced their emotional intelligence (eg, self-awareness and self-regulation); and increased their awareness of the importance of directly acknowledging others' accomplishments and contributions. [C/M] - Hackworth, 2018<br><br>Analysis of qualitative survey data provided by division directors indicated that on completion of the CLP, participants demonstrated improvements in leadership ability, confidence as a leader, listening skills, conflict management skills, and cross-disciplinary collaboration. [M/O] - Hackworth, 2018<br><br>Leadership program participants completed baseline assessments of their leadership competence and received 360-feedback from a supervisor, peers, and direct reports to identify strengths and improvement opportunities. They also completed the Myers Briggs Type Indicator and the Thomas Kilmann Conflict Mode Instrument. [C] -Hopkins, 2018<br><br>In terms of actual changes in leadership behavior, the self-reported distribution of work within project teams gradually shifted from the leader more to team members, as leaders acquired more team leadership skills. [M] - Hopkins, 2018<br><br>AL / CAL approach. Action learning (AL) is an experiential learning method in which participants learn by doing and then reflecting on what they have done. [C] - McCray, 2018<br><br>Not just listening for the sake of listening or paying lip service or pretending to

	listen or just. . .you know. But actually listening and listening to the detail and the importance and judging the importance of that detail and internally processing that in a way that is helpful to me and to the organization and the patients that I treat, and it comes back to that (AL4). - [M/O] - McCray, 2018<br><br>Figure 4 illustrates how some learning activities were considered to be especially useful and there was some suggestion that components such as 'self-understanding and interpersonal side of leadership' increased in perceived value over time. Participants emphasised the importance of self-understanding in order to improve working with others: The module where they tried to understand people, their personalities and the way they behave and how to interact with people was very useful because sometimes somebody may have difficulties, so [understanding] how to approach people and why they are reacting the way they are was very useful. (Int15) [C/M] **-** Miani, 2013<br><br>Participants reported that the taught modules mostly contributed to developing their leadership in areas such as confidence, communication skills, negotiation skills and team management skills. [C/M] **-** Miani, 2013<br><br>Skills were further developed by means of one-to-one coaching sessions delivered by QFI, alongside more informal coaching provided by mentors. One interviewee noted how individual coaching had 'helped understand yourself; and this is part of being a leader' (Int4), while another Fellow commented on the value of coaching being 'flexible' and tailored to the needs of the individual (Int1). [C/M] **-** Miani, 2013<br><br>This finding was supported in interviews, with mentions of opportunities to work better as a team (Int7) and to improve people management considered a strength of the Programme: The team work, the group work, I mean talking about different people and how we impact, how we think about making changes for the benefit of the patient. All this is happening as well and I think this is a good change and if we keep doing that it will make a huge difference in the communication and eventually it will make a great difference to the patient. (Int15) [M/O] **-** Miani, 2013<br><br>There was an overarching view that a perceived proactive and positive attitude of Fellows may have contributed to cultural change in the Trust. Interviewees reported that they tried to share what they had learned and so influence the work culture at the Trust (Int1, 5 and 7): I have been able to pass these skills on to my colleagues too. (Int7) [M/O] **-** Miani, 2013<br><br>In the CI interviews, participants also noted that the EI learning module, and the associated assessment and coaching provided by the session facilitator, helped them gain greater insights into their strengths and weaknesses. For example: I think that the emotional intelligence part really helped me... coaching me to really challenge myself to do things that the people in the room may not be happy with, but to do it in such a way... that you actually can walk away having disagreed and still having everyone say 'Okay, that was the right decision.' [C/M] - O'Neil, 2019<br><br>Surgeons also reported an increased level of self-awareness after participating, particularly referring to the 360-degree personal evaluations they underwent. One surgeon said the program "helped me understand how others view me and my interactions." Surgeons identified certain aspects of their behavior in leadership roles that they had not recognized previously, with 1 participant realizing that he/she always "appear busy. frazzled. unapproachable." These "self-revelations" were considered an effective portion of the program,

	enhancing surgeons' understanding of their own strengths and weaknesses as leaders in surgery. [C/M] **-** Pradarelli, 2016<br><br>Next, the satisfaction of having a coach to assist participating surgeons in developing a personalized leadership plan appeared to depend on both the individual coach and the individual participant. We found that a few surgeons perceived their coach to be less effective and felt that "the coaching was expensive and did not pay off." Some of the coaches with less experience tended to not be able to "make an impression, [and were] too generic." In contrast, surgeons who had more experienced and reputable coaches found the coaching sessions "insightful and nice to have [C] **-** Pradarelli, 2016<br><br>In line with the team-building curricular domain, participants reported an improved ability to foster collaborative relationships among team members. Some reported specific changes in behavior, such as "giving feedback, both positive and negative," and exercising patience. Other comments focused on the collegial environment that the leadership program created among the participating surgeons themselves. For example, one participant perceived the program to be a "morale-boosting event, bringing people together to bond over a common goal." [M/O] - Pradarelli, 2016<br><br>All participants engage in extensive self-analysis using standardized tests such as the Myers Briggs personality test34 and the Leadership Skills Inventory 360◦. [C] - Sanfey, 2011<br><br>Participants commented about gaining personal insight into strengths (25% of participants) weaknesses (22% of participants), or generally becoming more self-aware (25% of participants). [C] - Sanfey, 2011<br><br>Twenty-six (94%) participants indicated that they had changed their professional behavior as a result of LAM. Comments about a change in relationship behavior dominated, for example, becoming more encouraging (18% of participants): "I have tried to reach out more to others, to bring them into what I do and find out what they do" (M). Six (21%) people commented about gaining insight into oneself (2 participants), "I find I am much more reflective about my interactions with others" (W), or into others (4 participants). Sixteen (57%) respondents had made a change in their personal life. These changes included a change in communication with friends or family, "Listening more to what the other person is saying" (M), or taking up a hobby or activity. [M] - Sanfey, 2011<br><br>This change was expressed as a need for personal growth (36% of participants): "I realize the responsibility I have to act better, as I am the example" (W), "I am less concerned about being forceful as a leader or right, and more focused on leading group to a consensus that everyone is comfortable with" (W), and "Prior to course, I thought a good leader was someone who was good at getting people to do something by force of will. Now I see that someone is a good leader if they are aware of how they and others operate" [M] - Sanfey, 2011<br><br>Establishing a supportive, nonjudgmental environment allowed participants to release their feelings and anxieties about the task ahead of them: "I feel extremely vulnerable about what I'm getting into, but sharing your experience is very comforting." However, nurturant effects may be discomfiting, for example, by shaking complacency: "I was quite happy before I came here, now I'm stressed!" [C] **-** Shah, 2013<br><br>Heightened self-awareness helped participants to examine their existing self-perceptions, and also realize that success in their leadership role would depend

	on others' perceptions of them: "I never thought about how others see me. It has helped me to learn to know my limits." [C] -Shah, 2013<br><br>Miller; "I personally have done a number of EI measures and always learn something about myself and ways to improve the way I interact with people". [C/M] - Smith, 2014<br><br>The interviews and survey responses yielded several ideas to enhance the next PLA session: topic areas, timing of team projects, better explanations of how to work with a coach, how to make the most of a 360 evaluation process, and how to enhance usage of the SharePoint site. [C] - Throgmorton, 2015<br><br>The DiSC 360 was pretty enlightening to show me about myself and how to interact with different types of people [C/M] - Throgmorton, 2015<br><br>Biggest encompassing thing actually gets back to the personalities. I don't walk around saying oh there's a C and there's an S and there's a D, but I do think that I am a little more aware of where there is a different personality in front of me and I think that as physicians and surgeons specifically that if you paint us with a brush, okay this is how we do it and come along, what is it going to take for me to convince you, and I do think that I am a little more aware of having to chance my communication [...] tailoring how the conversation is going to go (depending on differing needs). [C/M] - Throgmorton, 2015<br><br>Summary of what was shared in an interview: A participant shared how she applied skills and encouraged a partner to do as well and it led to better cross departmental communication and problem solving. [M/O] - Throgmorton, 2015<br><br>While interviewees valued the domain knowledge/skills relating to practical QI approaches, many reported the content and practice opportunities relating to social, behavioral and emotional skill development as being the most impactful aspect. This has helped them become more confident clinicians who are now willing to take appropriate risks, make mistakes and learn from failures: I'm much more philosophical when there's problems at work so I'm trying to give people time and space to reflect on the problems that they've encountered. It's something developed in the Fellowship that's gone into my clinical arena and made me a better clinician overall (I7, Hospital Consultant). [C/M] - Toma, 2020<br><br>The training centers on self-reflection, critical evaluation, and basic leadership skills (e.g., how to run a meeting, how to take and give feedback, public speaking, etc.). [C] - Tsoh, 2019<br><br>At an individual level, the program fostered development of leadership skills in conflict resolution, team management, and giving and receiving feedback. Since completion of the FLC, a majority indicated noticeable changes in leadership skills (98.6%), and attitudes or behaviors related to leadership (91.7%). [C/M] - Tsoh, 2019<br><br>Qualitative responses supported impacts of participation in the FLC on personal growth, particularly in increasing self-awareness, confidence, and aspiration. [M] - Tsoh, 2019<br><br>Another participant indicated, '... [it] made me more confident as a leader and yet more willing to listen to others and give credit to them for their ideas.' [M] - Tsoh, 2019

	Some graduates described experiencing increased collaboration across and increased cohesion within departments or units. One graduate commented, '…helping me be a better leader within my unit, and helping my unit by serving as a bridge to other people across UCSF. [M/O] - Tsoh, 2019<br><br>When asked to reflect on how the LDP affected leadership style, many participants perceived that their approaches have become more collaborative and cited personal growth in a variety of areas, including the following: paying greater attention to strengths and weaknesses of colleagues, improving listening skills, decreasing agenda setting, and improving the ability to delegate. This transition has contributed to a more participatory approach to how decisions are made in the Department of Surgery at the University of Michigan. [M/O] - Vitous, 2019<br><br>Participants also described how relationships with colleagues in the department have changed since participating in the LDP. When prompted to reflect on how these relationships have changed, some surgeons pointed to areas of personal growth, such as becoming less sarcastic and having improved communication with colleagues. [M/O] -<br>Vitous, 2019<br><br>A more prevailing theme, however, was how these areas of personal growth translated to an improvement in the collegial environment, including an increase in morale and strengthened relationships. In discussing how the LDP influenced relationships, one surgeon asserted that the program "changed the environment" and "the framework with which I bring conversation" (participant 3). [M/O] - Vitous, 2019<br><br>Thematic analysis demonstrated that participation in a leadership development program influenced surgical culture in the following ways: (1) promoted a more participative leadership style, providing tools for surgeons to create a more collaborative environment; (2) increased the culture of diversity, with leaders in the department valuing a more inclusive and wide range of skill sets; and (3) strengthened the collegial environment as evidenced by improved morale and relationships within the department. [M/O] - Vitous, 2019<br><br>Mindfulness Leadership Course: These theories were chosen because of their relatedness to mindfulness. Each introduces overlapping themes of becoming more aware of self and others, being more in the present (instead of in the past or future), being more in the being-mode (vs the doing-mode), practicing more conscious (instead of automatic) decision making, listening more carefully and becoming more sensitive to employees' needs. [C/M] - Vreeling, 2019<br><br>The leadership program enhanced the following outcome categories:<br>A. Self - Attitude: Psychological attitude towards self.<br>B. Self - Behaviour: Behaviour towards self.<br>C. Other - Attitude: Psychological attitude towards others.<br>D. Other - Behaviour: Behaviour towards others. [C/M] - Vreeling, 2019<br><br>No. 14 – "...then it's listening, isn't it, really just listening and nothing else, just listening." No. 16 – "...being more open towards that and asking more questions. Asking 'why', 'can you pinpoint why exactly you aren't doing well?' 'Can you articulate what you want, what you need?' That I guess, asking more questions and more, well that means not drawing your own conclusions, but getting more to the bottom of things." No. 3 – "(...) then I just show there's nothing wrong with admitting you don't know something. Or that you'd like to use someone else's expertise." [M] - Vreeling, 2019

Intentionally building professional networks (CMO2)<br><br>If LDPs stimulate interaction between program participants [C], physicians build professional networks [M], which may impact the organization's culture [O_1], quality improvement [O_2] and the leadership pipeline [O_3]. When participants are from the same organization, professional networks seem most effective for realizing organization-level outcomes [C].<br>• Due to building professional networks, physicians gain understanding in the perspectives of others (e.g., administrators, other medical disciplines) and collaborate better. Networks also function as support structures [M], benefitting the organization's culture [O_1].<br>• Professional networks mobilize resources: physicians know where to go for collaborations or when facing challenges [M], leading to more effective quality improvement [O_2].<br>• Due to building professional networks, physicians become more visible within the organization and are more likely to be promoted [M], strengthening the organizations' leadership pipeline [O_3].	
Studies that provide (partial) evidence	Fassiotto, 2018; Ferris, 2018; Hopkins, 2018; Howell, 2019; Levine, 2015; Lewis, 2021; Maddalena, 2015; Miani, 2013; Monroe-Wise, 2016; O'Neil, 2019; Sanfey, 2011; Throgmorton, 2015; Toma, 2020; Tsoh, 2019; Vitous, 2019.
Illustrative supporting evidence (text fragments, descriptions, data)	I met people who I still have interactions with. That was the best. I made connections helpful from both work and personal perspectives. After 3 or 4 of the meetings, people were comfortable with each other and could say whatever (Female, Professor). [C/M] - Fassiotto, 2018<br><br>Finally, one of the most lasting pieces of the program beyond projects was the networking opportunity provided: The most lasting thing about [SLDP] is that meeting the other people helped me going forward. I learned how our interests were mutually beneficial. During the downtimes you get to know each other. Going forward, it has helped me because now I know these contacts and who to go to for different things (Male, Assistant Professor). [C/M/O_2] - Fassiotto, 2018<br><br>Participants were also asked which of the school's tripartite mission areas their projects impacted. A total of 18 of the 20 projects had impacts on patient care. Overall, ten projects impacted all three mission areas. Participants noted that projects were valuable both as a learning tool as well as a way to gain visibility within the institution. [C/O_3] - Fassiotto, 2018<br><br>The International Palliative Care Leadership Development Initiative (LDI) was a model demonstration project that aimed to expand the global network of palliative care leaders in low- and moderate-resource countries who are well positioned to apply their new leadership skills. [C/M] – Ferris, 2018<br><br>It is nearly impossible to capture and summarize the exhaustive details of what this energetic and powerful group has accomplished. Graduates of LDI have already demonstrated that they are the next generation of local, national, and global palliative care leaders. They have taught, published, networked, and served as visionary directors and/or coordinators of university pain and palliative care units, foundations, international, and national associations. [M] – Ferris, 2018<br><br>The program consisted of six sessions, each lasting one and one-half days, spread over 9 months, and held at a conference facility away from the institution. This supported participant networking and interspersed periods of learning with practice of newly acquired approaches. [C/M] -<br>Hopkins, 2018<br><br>The networking component of the PLA is a popular and key component of the

	program and includes an unparalleled opportunity for networking with pathology chairs at a variety of institutions nationwide, as well as fellow participants, and the APC's senior fellows, who are former pathology chairs. This latter group is a unique and valuable asset providing broad perspective and a safe non-power-based source of advice. [C/M/O_1,O_3] - Howell, 2019<br><br>The LPWF emphasizes networking within cohorts and with guest lecturers who include women institutional leaders and school of medicine deans. This provides participants with the opportunity to practice important networking skills and expand networks as they gain exposure to a diverse group of women colleagues and institutional leaders. Levine [C/M/O_3] - Levine, 2015<br><br>The program provides a safe environment for women to network with other women interested in leadership and for participants to explore their own leadership identity through the content and educational activities. An underlying theme of the program is to raise awareness of gender stereotypes and their impact on women's careers. [C/M] - Levine, 2015<br><br>I enjoyed this time to develop skills and reflect and interact with others facing similar challenges. I learned how important networking is. The program put me in touch with other women in similar places. Most important was networking with other women in the institution. The contacts that I have made are important to me. Allowed me a broader perspective of my role in the organization and helped me to feel more connected to the organization itself. Being part of a group of bright women who are considering these and other issues together was very helpful. [C/M/O_1,O_3] - Levine, 2015<br><br>Successful IAP implementation helped fellows enhance their visibility and reputation. Projects that aligned with institutional priorities enhanced the reputation of both the institution and fellow, either directly through project outcomes, or indirectly through development of the fellow as a future leader. When projects enhanced institutional missions and organizational culture, fellows had new leadership opportunities and their IAPs were more likely to achieve intended goals. [C/O_3] **-** Lewis, 2021<br><br>Several participants reported that the opportunity to network and collaborate with other physician leaders and managers was one of the most valuable aspects of PMLP. Several participants indicated that they had, in fact, participated with colleagues whom they work with on a regular basis and reported that this level of team involvement added to the experience as they could directly collaborate on projects during the program, and could see how/why they can collaborate in future. [C/M/O_3] - Maddalena, 2015<br><br>Several participants commented on the value of being offered, by the Program, in relation to the opportunity to meet and work with other staff they had not previously worked with. Improved collaboration was seen by some participants as one way to maximize efficiency through understanding how other staff in the Trust operate (Int5, 9), overcoming silos (Int4, 9, 12) or avoiding duplication of efforts (Int6). [C/M/O_1,O_2] - Miani, 2013<br><br>In the same way, some Fellows reported to having had difficulties in accessing relevant datasets for their projects; however, some acknowledged that networking opportunity provided by the Program had provided means to overcome this challenge. [M/O_2] - Miani, 2013<br><br>Almost all (97 %) Afya Bora alumni remained in contact with colleagues from the fellowship. Of these, alumni were most frequently still in contact with other

	Afya Bora fellows (90 %). Primary mentors were the next most common type of colleague that alumni were in touch with (71 %), followed by attachment site mentors (65 %). Additionally, 21 alumni (70 %) remained directly involved in Afya Bora-related activities, such as ongoing research or other projects with attachment site personnel. [M/O_2] - Monroe-Wise, 2016<br><br>With its interactive, case-based didactic curriculum, experiential attachment site rotations rooted in strong mentorship, and cultivation of interprofessional networks through multidisciplinary cohorts, the Afya Bora training program embodies these important principles. Indeed, in describing the professional successes they have enjoyed since completing the fellowship, Afya Bora's alumni frequently cite skills learned during both didactics and attachment site experiences, multidisciplinary cohorts, mentorship and networking as valuable aspects of the training curriculum [C/M] - Monroe-Wise, 2016<br><br>I think the connection, the forming of mentorship and bonds, and being able to talk with people who I wouldn't ordinarily interact with because in [my department] we're part of our own little unit and don't really interact with a lot of others. And being able to get different perspectives of what's going on in the whole health system - I think that was invaluable. [C/M] - O'Neil, 2019<br><br>The sponsors noted that the opportunity to work on team projects related to organizational topics of interest had a positive impact on the participants' networking and interpersonal relationships and improved trust and collaboration amongst the team members. [C/M/O_1] - O'Neil, 2019<br><br>Career advancement. When asked to list additional benefits received from LAM, "networking" was identified by 12 people: "Connections with faculty outside my own orbit" (M) and "The ability to meet my peers in other departments was very valuable" (M). Two people identified the significance of networking in terms of "Making valuable career contacts" (M), but 6 people identified the significance of networking in terms of "Building a sense of community" (W) or support, "We support each other" (W). Sixty-seven percent of the participants have been offered a leadership position since completing LAM. Sixty-six percent of men, compared with only 26% of women, were seeking leadership opportunities, $\chi2(1) = 8.124$, $p < .05$. [M/O_1,O_3] - Sanfey, 2011<br><br>I had no idea what to expect when we started and I was pleasantly surprised that there were so many doctors from different specialties and different locations that I had never met before. It was a nice opportunity for us to discuss issues that were common between units and specialties even though we all work in very different fields. [C] - Throgmorton, 2015<br><br>Overall, what I liked is that it seemed like there was a strong administrative presence throughout the PLA that was very encouraging, very respectful, and very helpful [...] Going through it, it was a great opportunity to meet with and bond with other healthcare professionals as well as get to work on a level playing field with administration and to see that we are all in this for a common good. [C/M] - Throgmorton, 2015<br><br>I know there is a support network. [M] - Throgmorton, 2015<br><br>I am really looking forward to taking that from class and then with these contacts, with these friends, being able to do things in the hospital later on. [M/O_2] - Throgmorton, 2015<br><br>The opportunity to network and make connections, the comrade support from

	an ever-expanding family of fellows and experts assisted with personal growth in terms of breaking down professional boundaries and removing stereotypes so I would say, stick your head above the parapet and forget about the impostor syndrome. (I4, Allied Healthcare Professional) [C/M/O_1] - Toma, 2020<br><br>The FLC aims to facilitate networking among its participants within the AHC through a cohort-based model of training. [C] - Tsoh, 2019<br><br>Graduates described how the program led to new collaborations. Most (90%) reported increased interpersonal leadership skills and 85% indicated their participation helped build a network of faculty leaders transcending internal departmental boundaries. Two-thirds (67%) said their participation gave them better understanding of group dynamics. Post FLC completion, 69% and 50% said they continued to interact with participants from their or other cohorts, respectively." [C/M] - Tsoh, 2019<br><br>Another way in which the LDP helped to shift the collegial environment was the increased opportunities to network and interact with colleagues. Participants described how one of the greatest advantages of the LDP was the guaranteed protected time that offered participants the opportunity to interact in ways that are often not possible in clinical settings. Although these interactions offered personal benefits, such as opening the doors to unique research opportunities, it also led to a change in how communication happened both within and outside of the department. [C/M/O_1,O_3] - Vitous, 2019<br><br>Surgeons also described the ways in which the department has become more diverse as a result of LDP. Specifically, participants reflected on how their divisions felt more inclusive, with a more diverse range of skill sets being deemed as valuable or important. Contrast this to how participants reflected on the culture post LDP, where sentiments reflected a shift in the culture of diversity, "There's been an increased sort of very public recognition of the value of diversity in the department, right? So, I guess I would say that the culture of the department has probably been more publicly pro diversity and pro, you know, promotion of people, of a variety of people" (participant 4). [O_1] - Vitous, 2019
<u>Supporting quality improvement projects (CMO3)</u><br><br>If LDPs include well-supported quality improvement projects (i.e., coaching, project management support, funding, protected time, facilities) endorsed by the organization [C], this allows physicians to create buy-in and be more perseverant when facing challenges [M]. This increases the likelihood of successful implementation of the project and quality improvement [O].	
Studies that provide (partial) evidence	Berghout, 2020; Bhalla, 2018; Christensen, 2016; Daniels, 2014; DeRusso, 2020; Fassiotto, 2018; Hopkins, 2018; Leggat, 2016; Miani, 2013; Nakanjanko, 2015; O'Neil, 2019; Rao, 2017; Rask, 2011; Smith, 2019; Throgmorton, 2015.
Illustrative supporting evidence (text fragments, descriptions, data)	In addition to the collective sessions, every participant carried out a hospital-based improvement project (see Appendix 1 for project examples and content of the programme). [C/O] - Berghout, 2020<br><br>Many interpreted the leadership programme as a means to deal with colleagues 'who do not want to change' and to learn 'how to cope with frustration' and to 'keep going'. [M] - Berghout, 2020<br><br>In addition, they were not always granted the extensive time required for executing improvement projects because the daily pull of clinical work was

	perceived as too strong. [M] - Berghout, 2020<br><br>Fellows are required to design and lead a quality improvement project—the capstone. The project is conducted with an interdisciplinary team of clinicians at the fellows' home institutions to advance organizational or departmental quality or patient safety goals. The capstone is intended to be the practical application of coursework and must be endorsed by the leadership of the fellow's organization. Each fellow is assigned to a faculty member who serves as a mentor to guide him or her through the capstone process, and to navigate potential organizational challenges. [C] - Bhalla, 2018<br><br>Another participant could not fulfill the capstone project requirement largely because of lack of leadership support. [C/M] - Bhalla, 2018<br><br>Time spent by fellows on capstone projects was not formally quantified as part of the present evaluation, fellows noted that the commitment was significant, encompassing several hours per month, both during work and off-hours. [C] - Bhalla, 2018<br><br>A descriptive summary of the 87 (74 physicians and 13 nurses) fellow capstone projects completed during the 5-year period is summarized in Table 3. Projects were highly varied in nature and scope. They encompassed efforts to improve safety, effectiveness, efficiency, timeliness, and patient-centeredness. Three categories comprised 51% of all capstone projects: (1) improving efficiency in inpatient or emergency department settings; (2) improving transitional care among inpatient, primary care, or other settings; and (3) reducing hospital-acquired infections or improving sepsis care. [O] - Bhalla, 2018<br><br>This course is also designed as an innovation incubator, with each attendee invited at course inception to propose an idea that he/she would like to implement. The top 12 ideas that the group most prefers are culled from the 70 submissions, and multi-disciplinary teams then form around each idea, with each team developing a full business plan over 10 months for implementation that is ultimately presented to Cleveland Clinic leadership at the final session. [C] - Christensen, 2016<br><br>Success as an innovation incubator is supported by the fact that 61% of the business plan ideas either have been implemented or have extinguished an idea that the team itself felt was infeasible based on fuller analysis. [O] - Christensen, 2016<br><br>This participant identified course materials from the intervention that informed her ongoing development in health leadership, and she referenced the important ongoing support her mentor provided in helping her implement the project. She stated that 'My primary mentor was an obstetrician like myself....he would sit down with me to figure out what needed to be done [on the project].' [C/M] - Daniels,2014<br><br>Nominations were reviewed to confirm that individual and departmental goals were aligned with the program and the institution. [C] - DeRusso, 2020<br><br>The experiential learning involved the development of a project focused on the area of expertise and the current leadership role of the individual participant. [C] - DeRusso, 2020<br><br>Several projects led to hospital funding based on their impact on clinical care and potential for innovative discoveries. [O] - DeRusso, 2020

	Participants had coaches and sponsors for their projects. See evaluation survey in article and paper Hopkins (same program). [C] - Fassiotto, 2018<br><br>Participants were also asked which of the school's tripartite mission areas their projects impacted. A total of 18 of the 20 projects had impacts on patient care. Overall, ten projects impacted all three mission areas. Participants noted that projects were valuable both as a learning tool as well as a way to gain visibility within the institution. [O] - Fassiotto, 2018<br><br>Participants were asked to estimate how many individuals their projects had impacted since program completion: 8 (40 percent) estimated that their project had impacted 10s of individuals; 5 (25 percent) estimated 100 s; and 7 (35 percent) reported that their SLDP projects had impacted 1000 s of individuals since implementation. This impact continues to grow: [My project] was fairly successful [at the time] – but the continued progress went beyond that. It's mushroomed. Now the benefit is an order of magnitude greater (Male, Assistant Professor). [O] - Fassiotto, 2018<br><br>In-house leadership development programs offer the advantage of greater alignment between program competencies and institutional priorities. [C] - Hopkins, 2018<br><br>An executive sponsor was required for each project, and a coach skilled in team process management worked with the leader. Projects were monitored at several points during the program both by the coach and through project progress reports submitted by participants. [C/M] - Hopkins, 2018<br><br>Over the full four program years (2008-2011), participants completed projects across a wide variety of areas including quality and process improvements (51%), new clinical programs (24%), business plans (10%), new research programs (9%), and new educational programs (7%). A number of important results were achieved including improvements in quality of care, patient safety, and efficiency of care processes; enhanced patient satisfaction; and new program development (Table 5). [O] - Hopkins, 2018<br><br>The program participants far exceeded expectation with 58% reaching level 3.0 (moderate improvement in process measures) and over one fifth of participants (22%) attaining level 4.0 or greater, indicating significant improvement in outcome measures. [O] - Hopkins, 2018<br><br>The workplace project required the participants to identify, plan, implement and evaluate a quality or safety initiative in their workplace. The project evolved and was progressively implemented in parallel with the simulation, online learning space and face-to-face workshops. The final workshop required the participants to present their projects in a scenario revolving around a 'minute' with the State Minister of Health. [C/M] - Leggat, 2016<br><br>Participants reported that many projects, with their focus on practical improvement and change, had resulted in distinct and recognizable changes to practice. They addressed a wide range of clinical practice and service provision and often focused on areas of practice highlighted in the Australian National Safety and Quality Health Service Standards. [C/O] - Leggat, 2016<br><br>In most cases, participants perceived the workplace projects to have a significant impact in terms of early outcomes, impact and reach. The majority had plans to extend the project or use it as a starting point for other quality

	improvement initiatives. Almost all participants and sponsors indicated they would continue to work on or support the project and its extension or evolution and many had specific plans to do so. Some organizations had the projects already built into their strategic and operational program indicating evidence of early embedding. This suggests the program has contributed to the type of capacity building that results in sustainable outcomes. [C/O] - Leggat, 2016<br><br>Projects that aligned with institutional priorities enhanced the reputation of both the institution and fellow, either directly through project outcomes, or indirectly through development of the fellow as a future leader [C] - Lewis, 2021<br><br>Project work had bidirectional impact on the fellows in the program and on the institution itself. Project enablers included: focusing projects on institutional priorities, obtaining sustainable support, and navigating institutional complexity. Leading indicators of institutional outcomes included contributions to institutional leadership and culture, and mutual enhancement of the reputation of the fellow and of the institution. [M/O] - Lewis, 2021<br><br>One core feature of the program was protected time for participants to engage in quality improvement activities, which was perceived as one of the key enablers of program success, along with the strong support by members of senior management. Other reported enablers included dedicated project management support, and the commitment of the program participants. Key challenges included financial and time constraints, staff resistance to change, and short program duration. [C/M] - Miani, 2013<br><br>As part of the scheme, clinical Fellows were paired with established senior staff (clinical leads or clinical directors) of whom some acted as mentors to support the implementation of Fellows' projects. [C/M] - Miani, 2013<br><br>When asked about the value of the program activities in relation to the delivery of QI projects (Figure 5), some features of the program were seen as very useful. These included the dedicated project management support and the taught sessions on project management and business planning. [C/M] - Miani, 2013<br><br>Participants identified a number of key enablers and challenges to delivering their QI projects. The most frequently mentioned enablers included: dedicated time to conduct the project, the contribution of motivated individuals, availability of resources, support from other staff, senior buy-in, teamwork or collaboration with other Fellows and participants, and QFI support. The main identified challenges mirrored the enablers, including time constraints, lack of financial resources, resistance of other staff, lack of middle and/or senior management support, limited duration of the program, and staff shortages (Table Interviewees further highlighted the role of mentors in supporting the delivery of QI projects, with two participants commenting how mentors were able to facilitate relationships between members of the Trust and to link with senior staff or non-clinical departments (Int5, 10). [C/M] - Miani, 2013<br><br>Duration of the program was closely linked to the sustainability of program outputs. Most participants expressed concerns over the sustainability of their initiatives and the possibility that with the end of the program, focus and resources which facilitated progress might be diverted: It's about how we maintain that through... But I am conscious of workload, and because we do not have time to do it... I hope it's not going to fall apart; all the things that they've put in place will hopefully stay here, but it is about how we embed that process. (Int12). [C/M/O] - Miani, 2013

	Participants reported on a series of key outputs from their QI projects; these included better team-working and increased communication within and across teams (see Section 'Working better as teams'). It was beyond the scope of the evaluation to systematically collect data on quantifiable outcome measures of individual QI projects. However, program participants did report on measurable clinical outputs that contributed to improvements in the quality of care. Given the nature of the evaluation and to maintain respondents' confidentiality, we here refrain from directly citing individuals' experiences, and instead document a small selection of high-level experiences. For example, one internal Fellow noted how their QI project had substantially enhanced patient experience in maternity services, which was seen as an important achievement by Scheme B participants. Other QI projects in maternity were also reported by some participants to have equally led to tangible measures of success, such as relieving some of the pressures in the maternity ward through greater use of ambulatory services. Similar successes were reported by scheme A participants. [O] - Miani, 2013<br><br>There appeared to be a relative lack of external dissemination of QI project findings (Figure 8), which was noted by one interviewee as presenting one of the weaknesses of the program (Int15), in particular against the perceived aims of the program to contribute to improving the Trust's reputation (see Appendix B). While overall the level of external dissemination of QI project findings was lower compared to internal dissemination mechanisms (Figure 7), Figure 8 highlights that dissemination activities did increase as the program evolved, with around one third of participants reporting to have given external presentations towards the completion of the program. [O] - Miani, 2013<br><br>Matching fellows to projects that were mutually beneficial to the fellow and institution was considered extremely important to the success of the attachment site experience although it required time, in some cases up to 8 weeks [C/M] - Nakanjanko, 2015<br><br>To qualify for accreditation, attachment institutions had to demonstrate availability of suitable infrastructure (e.g., office space to host the fellow and a variety of projects in which fellows could participate) as well as potential mentors to support fellows' training. Attachment site rotations allow learners to apply concepts and skills learned in the classroom to real-world problems faced by local health organizations. [C/M] - Nakanjanko, 2015<br><br>Having the fellows as leaders of their own attachment site experience required a paradigm shift from most in- service training programs that detach trainees from a work environment in a search for protected time. Mentorship by attachment site mentors from the respective sites and primary mentors from the Afya Bora program was critical to development of locally relevant projects. [C/M] - Nakanjanko, 2015<br><br>Overall, 14 (93%) of the 15 fellows conducted mentored projects, covering a wide spectrum of HIV/AIDS care and health system challenges, as highlighted in Table 1. [O] - Nakanjanko, 2015<br><br>President and CEO at THS, stated that, "Our physician leaders worked collaboratively to tackle some of the most pressing issues, and the recommendations they made during their Capstone presentations were among the most innovative ideas I've heard all year." The AL teams have continued to work together to advance their projects into the broader organizational system. [C/O] - O'Neil, 2019

	While more time in AL team meetings would have resulted in more in-depth project outcomes and opportunities for personal development, results shown from follow-up and evaluation demonstrated AL has impact even in a short time period. [M/O] - O'Neil, 2019<br><br>Teams with sponsors who kept the momentum going tended to continue working on their projects; whereas teams without a 'push' from their sponsors, tended to lose steam on their projects. As such, we learned that designing a structured process for AL teams to continue project work post-graduation is crucial to sustaining the learning and momentum of the program. [M/O] - O'Neil, 2019<br><br>Second, course directors need to determine how participants will apply their QI skills during the course. Real-world projects immerse participants in the challenges of stakeholder engagement and rapid cycle testing. If successful, this work can produce tangible results. However, these projects often present unexpected challenges for QI learners. CPIP augments learning from team projects with case studies that reinforce key concepts by removing some of the unpredictable features of real-world projects. [C/M] - Rao, 2017<br><br>Quality leaders review applications from their respective sites and select applicants based on the candidates' leadership potential, the alignment of the project proposal with the institution's goals, and the degree of support the candidates have from their sponsor. [C] - Rao, 2017<br><br>Additionally, each CPIP team has a coach to help participants master the skills taught in the course and also to help keep projects moving between teaching sessions. Coaches are assigned 1 to 2 teams. Their responsibilities include reviewing homework prior to each session and supporting teams to develop their final presentations. Previous CPIP graduates serve as coaches for current attendees, which helps broaden the learning resources for new students and reinforce previous training for coaches. [C/M] - Rao, 2017<br><br>Sixty-six percent (90/136) reported continued work on their course project, with 28% (38/136) having published their findings or presented their work externally. Sixty-two percent (84/135) reported working on another QI project utilizing the tools taught in the course, while 64% (87/137) taught the concepts to others. See table for example project and results. [O] - Rao, 2017<br><br>A key component of the training program is the design and implementation of a QI project. During the first year of the April 2011 program, project topics were open ended, allowing participants to select any priority area in their operating units. As the course evolved, however, teams were matched with topics that were a high priority for the health system. Project teams are formed after the first learning session and begin weekly meetings to advance their project work. Attendees are paired with a mentor who has experience in improvement methodologies and team leadership. The mentor relationship was designed to facilitate the process improvement project and assist with team dynamics. Leaders from the participant's department are engaged to serve as a resource for identifying and overcoming implementation barriers. [C/M] - Rask, 2011<br><br>Nineteen (86.4%) of the 22 participants reported that their project had been fully implemented as originally planned. One participant never received the necessary resources to implement it, while two other projects became obsolete because of concurrent system changes that eliminated the original target. Some 13 (68.4%) of the 22 projects remained active. In addition to the 2 projects that became obsolete because of concurrent system changes, 2 projects ended

	because of a lack of sufficient resources, and 2 projects were dropped because of a lack of interest on the part of the implementation team. The 4 projects that were not sustained addressed some aspect of patient clinical outcomes. Of the 13 projects that were sustained, the majority (8) addressed changes in work flow—for example, altered appointment templates, standardized phone policies, restructured work responsibilities, and standardized processes—that facilitated patient access or operational efficiency. Of April 2011 the remaining sustained projects, 1 project obtained additional resources to meet documentation needs, and 4 led to permanent prompts or reminder systems that affect patient care processes (for example, falls, restraint use, newborn care). [C/M/O] - Rask, 2011<br><br>Initially, as stated earlier, participants were encouraged to identify any QI project relevant to their operating unit. An advantage of this approach was that participants could identify a topic about which they were passionate. However, a disadvantage was that many early projects were narrow in scope, and it was challenging for participants to engage other staff and the resources required for effective implementation. Participants are now encouraged to identify QI projects that align with institutional priorities, as identified by the yearly strategic plans. Participants and instructors report that aligning projects with institutional goals has facilitated buy-in and promoted mentorship by supervisors, increasing the likelihood of both sustainability and spread. [C/M/O] - Rask, 2011<br><br>In addition to classroom learning and assessment tools, each participant was assigned to a four-person team to design, plan, and execute a real-world project aligned with a departmental strategic priority. The decision to include such a project was based on the knowledge that leadership programs may fall short of their intended impact if they are decoupled from real-life work settings. [C/M] - Smith, 2019<br><br>RLA team projects emphasized four areas: (1) improving the patient or customer (eg, referring physician) experience, (2) improving the employee experience, (3) business process improvement, or (4) improved safety or quality of images or services. Nearly all of the team projects that were developed and piloted within the RLA have been partially or fully implemented in the department (Table 3). “Partially implemented” means the project was implemented within a subsection of the department, such as a single site or modality, or is currently in the full implementation phase. Projects often expanded into a sustainable process or program with positive business or cultural impact. Highlighted next are four such projects. [O] - Smith, 2019<br><br>Coursework is supplemented with the following experiences to enhance learning: working as a learning cohort, completing a behavioral style assessment, completing a 360 degree feedback process, working with a coach, participating in online discussion via a PLA SharePoint site, accessing self-directed online learning resources, and working on a team project in a small group. [C] - Throgmorton, 2015<br><br>Organization based results were evaluated via survey from a stakeholder audience to whom PLA participants presented their team based project work, 97 percent agreed the work of the teams illustrated alignment with the organization’s stated strategies. Project work yielded results after the completion of the program by offering some timesaving communication tips and through the production of a physician communication video now used to orient new providers. [C/O] - Throgmorton, 2015

Tailored LDP content prepares physicians (CMO4)<br><br>If LDPs' content is tailored to physicians' leadership needs and expertise [C], physicians perceive the LDP content as relevant, and the learning experience prepares (i.e., knowledge, skills, attitudes, confidence, self-efficacy, identity as leader) them for current or future leadership roles [M]. They are more willing to assume leadership roles and considered competent, leading to new leadership roles and strengthening the leadership pipeline [O].	
Studies that provide (partial) evidence	Berghout, 2020; Bhalla, 2018; Cohen, 2019; DeRusso, 2020; Ennis-Cole, 2019; Fassiotto, 2018; Fernandez, 2016; Gholipour, 2018; Hackworth, 2018; Hopkins, 2018; Leggat, 2016; Levine, 2015; Macphail, 2014; Maddalena, 2015; Miani, 2013; Monroe-Wise, 2016; Pradarelli, 2016; Rask, 2011; Sanfey, 2011; Smith, 2014; Throgmorton, 2015; Toma, 2020; Torbeck, 2018; Tsoh, 2019; Vitous, 2019.
Illustrative supporting evidence (text fragments, descriptions, data)	Physicians are known for safeguarding their professional identities against organisational influences. However, this study shows how a medical leadership programme enables the reconstruction of professional identities that work with rather than against organisational and institutional contexts to improve quality and efficiency of care. [M] - Berghout, 2020<br><br>Our findings reveal that when ‘new’ (organisational) responsibilities, such as multidisciplinary collaboration, are not backed-up by a supportive environment this may lead to identity violations causing stress and work dissatisfaction. Although the MLDP offered an important supportive space to discuss these identity violations, physicians also needed a supportive space within the hospital environment itself to not become ‘isolated’ leaders with unrealized collaborative ambitions. [O] - Berghout, 2020<br><br>The CQFP application process is competitive. It requires hospital leadership to nominate eligible clinicians and to commit to allowing participants time off from clinical and administrative responsibilities to satisfy program requirements. The program is tailored to physicians and nurses with limited experience conducting quality improvement and patient safety initiatives. Applicants are not required to have a prior or current quality or safety position, or training. [C] - Bhalla, 2018<br><br>All pre-post p<.05<br>“Use quality improvement tools”<br>“Measure quality/use quality data”<br>“Implement quality improvement initiatives”<br>“Use health information technology to improve quality and patient safety”<br>“Organize teams/teamwork” [M] - Bhalla, 2018<br><br>“The relevance of the simulation was also commented upon It was a brilliant introduction to the reality (P50) as was the contextualization of the issues Bit really encourages active learning and understanding (P54).” [C] - Cohen, 2019<br><br>“Changes in behavior post-simulation. Forty-seven participants completed the behavior questionnaires in full pre- and post-simulation (Table 2). Capability was the only construct that showed a large and significant change post-simulation (p < .001, Cohen's r = 0.619). There was a moderate but significant change in Behavioral Intentions, Attitudes and Subjective Norms post-simulation, although there was no significant change detectable in Opportunity.” [M] - Cohen, 2019<br><br>“Self-efficacy in clinical leadership. Forty-six participants completed the General Self-Efficacy Scale both pre- and post-simulation. There was an increase in perceived self- efficacy post-simulation (This difference was significant at p G .005, but only at medium levels of impact (Cohen's r = 0.43, z value = 2.794).”

	[M] - Cohen, 2019<br><br>In the short time between the simulation and feedback, 22 clinicians stated that their experiences in The Crucible simulation had directly influenced their leadership practice. For example, one participant explained how understanding integrated care had directly impacted; As a community paediatrician, I am now keen to be part of the discharge planning process for children with neurodisability and enable a seamless transfer of care closer to home (P30).Others expressed that they were able to contribute more to departmental strategy; I feel more confident that I understand NHS changes, and that many of my colleagues are unlikely to have more knowledge than me. This has given me confidence in expressing my views in management meetings (P24).[M] - Cohen, 2019<br><br>In 2014, the Children's Hospital of Philadelphia (CHOP) assessed the needs of physician leaders across the institution, including individuals in academic department leadership positions and individuals in hospital administrative leadership roles. [C] - DeRusso, 2020<br><br>To be eligible, candidates had to be a strong performer in an existing leadership position or recently selected for a new leadership role, committed to enhancing personal leadership skills, and available to attend all program sessions. [C] - DeRusso, 2020<br><br>The experiential learning involved the development of a project focused on the area of expertise and the current leadership role of the individual participant. [C] - DeRusso, 2020<br><br>Survey results indicated that all 125 participants from the 4 cohorts agreed their leadership skills were enhanced, they felt more connected to the institution, and they were committed to contributing to the enterprise-wide mission. [M] - DeRusso, 2020<br><br>Information from 93 participants in the first 3 cohorts showed that since completion of the program, 53% were in a new leadership role at CHOP, 38% were in a new leadership role nationally or internationally, 43% chaired a division or departmental committee, 14% chaired a hospital committee, 31% participated in a hospital enterprise plan, and 17% were promoted to a division chief or vice chair role. [O] - DeRusso, 2020<br><br>Executive Team Leaders used pre-program activities to select participants, collect information that shaped the program, determine physician's work preferences, establish curriculum, and help participants make the most of the program. Pre-program activities included nominations, assessments (360-degree and the Insight Profile), and pre-program interviews. Each of these items will be briefly discussed. [C] - Ennis-Cole, 2019<br><br>Following the leadership development program, nine out of the 10 participants graduated from the program and moved into new or enhanced leadership positions. Participants reported that their capacity for collaboration increased and their new leadership skills were utilized in their new leadership roles. [M/O] - Ennis-Cole, 2019<br><br>SLDP participants rated themselves higher than non-participants on 8 of 14 skills items. Of these items, participants rated their skills significantly higher than non-participants in ability to create and articulate a vision (p=0.02) and ability to carry out performance evaluations and provide constructive feedback (p=0.01).

	[M] - Fassiotto, 2018<br><br>SLDP offered participants an opportunity to learn about and work on their strengths and weaknesses, preparing them for current leadership roles: I thought it was an incredible gift. It gave me basic tools I didn't have before to be a leader within every team I work on. It helped me to understand myself better too (Female, Assistant Professor at time of SLDP participation). [M] - Fassiotto, 2018<br><br>Participants rated themselves higher than non-participants across all ten items on leadership attitudes. Across three of the ten items, focused on perceptions of support from the School and colleagues, ratings were significantly higher ($p<0.05$) for participants. [M] - Fassiotto, 2018<br><br>Recurrent interview themes included: the overall program value for current leadership roles. [M] - Fassiotto, 2018<br><br>The skills I learned were immediately transferrable to my leadership position. The program prepared me very well for my current role as department chair (Female, Associate Professor). [C/M] - Fassiotto, 2018<br><br>Program expenses were jointly covered by the School of Medicine and Hospitals and both School and Hospital leadership nominated individuals for the program based on their potential for leadership. [C/M] - Fassiotto, 2018<br><br>Asian program participants were significantly more likely than Asian non-participants to have been promoted, and women participants were less likely to have left the institution than non-participants. [O] - Fassiotto, 2018<br><br>Respondents from 2009 to 2011 cohorts were also asked about current leadership roles (Table III). A higher percentage of participants reported current roles across all leadership levels, although differences were not significant across School leadership positions. [O] - Fassiotto, 2018<br><br>Participants were, however, significantly more likely to hold regional or national leadership titles ($p=0.02$) and to have taken on new leadership titles since SLDP nomination ($p<0.01$). [O] - Fassiotto, 2018<br><br>We also sought to analyze whether program participation impacted retention. Overall, 24 (18 percent) of 131 SLDP participants departed Stanford since their nomination, compared with 16 (20 percent) of 82 non-participants ($p>0.72$). [O] - Fassiotto, 2018<br><br>And thus, it is desirous to both avoid redundancy with other learning opportunities and avoid teaching skills not relevant to the practical needs of the participant as that would result in poor use of scarce training resources. When the ACOG NLI course was initially conceived, the 10 skills evaluated were chosen because they were recognized as important or core skills for physician leaders by the planning team and were used successfully in the FSLI program. [C] - Fernandez, 2016<br><br>Fellows endorse the selection of the course content in the following ways: Fellows reported that the course was beneficial to their practice as physician leaders, the course allowed them to provide better healthcare for their patients, and indicated the communication skills and leadership approaches learned in the ACOG NLI were incorporated into their day-to-day work responsibilities (see Table 5). [C/M] - Fernandez, 2016

	"This course definitely was a life-changing experience. I have used many of the skills I learned to organize/run meetings, communicate more effectively with people, and seek out opportunities that fit my style better." [M] - Fernandez, 2016<br><br>Interestingly, a large majority of respondents reported receiving a promotion or other similar expansion of role opportunity since completing the course, and all who reported such a job expansion indicated that the skills learned in the course helped prepare them for the new opportunity (see Table 5). [M/O] - Fernandez, 2016<br><br>Of the 26 respondents, 16 (62%) indicated that they had received a promotion, had a change of job, or had taken on new leadership opportunities since completing the course. [O] - Fernandez, 2016<br><br>The Tabriz health management fellowship training program was developed based on the educational need assessment of the district health managers in the north-west of Iran and for training them; it was supported by the Ministry of Health, Treatment and Medical education as well as the health deputy of the Tabriz University of Medical Sciences. [C] - Gholipour, 2018<br><br>Based on the findings, most specialized areas of knowledge met the health managers' training needs including quality improvement, managing the district, planning and evaluation, epidemiology and advocacy, and community participation (table 5). [C] - Gholipour, 2018<br><br>According to pretest and post-test results, after training, district health managers obtained the highest scores in managing the district (77 out of 100), planning and evaluation (69), chronic disease management (69), human resources and creativity (68), and epidemiology (67). Also, health information (44) and health resources management and health economics (53) gained the least score among health managers. Finally, the courses on managing the district (51%), research in the health system (42%), and human resources and creativity (37%) had the most positive differences between pretest and post-test scores (figure 1). [M] - Gholipour, 2018<br><br>The impetus for program development was a needs assessment conducted by the OAACD that identified an urgent need for faculty leadership development, as well as the institutional strategic plan that identified a need to invest in structured leadership development at all levels. **[C]** - Hackworth, 2018<br><br>Analysis of qualitative survey data provided by division directors indicated that on completion of the CLP, participants demonstrated improvements in leadership ability, confidence as a leader, listening skills, conflict management skills, and cross-disciplinary collaboration. [M] - Hackworth, 2018<br><br>The mean scores for all 4 cohorts combined for the items assessed were as follows: "Overall, I was satisfied with the quality of this educational program," 6.5; "This training program will significantly enhance leadership and/ or management capabilities at my organization," 6.6; "I will be able to apply the knowledge and skills learned from this training to my job," 6.5; "I learned new knowledge and skills from this training," 6.5; and "This training was a worthwhile investment for my career development," 6.7. When asked if they would recommend the program to others, 94% responded yes, 6% not sure, and 0% no. [C/M] - Hackworth, 2018

	Analysis of qualitative survey data provided by division directors indicated that on completion of the CLP, participants demonstrated improvements in leadership ability, confidence as a leader, listening skills, conflict management skills, and cross-disciplinary collaboration. [M] - Hackworth, 2018<br><br>The curriculum (Table 1) was created based on competency models, institutional needs assessments, and recurring topics found in other programs. [C] - Hopkins, 2018<br><br>Table 4 shows significant improvements in respondents knowledge, skills, and attitudes throughout the program. [M] - Hopkins, 2018<br><br>The intervention was designed with a focus on enquiry-based learning (EBL), as recent research suggests that deeper learning that is more applicable to practice takes place when learners are presented with a scenario enquiry and are encouraged to understand the context and reflect on the wider implications. Given that the participants were senior clinicians, EBL seemed appropriate given that "EBL differs from problem-based learning (PBL) in that it is less directive and empowers students to take ownership of the course".21 (p. 85) Participants already had high levels of expertise. [C] - Leggat, 2016<br><br>The program was regarded by the clinicians who participated and their organizational sponsors as exceptionally valuable. They viewed it as making a significant difference in two major ways. First, it strengthened their knowledge and skill in the field of quality and safety, their understanding of the thinking and rationale behind quality and safety initiatives and the evidence regarding their importance and impact. In addition, the interview participants reported a new-found appreciation for the importance of the patient and consumer in assuring quality and safety. It completely changed the way I practice medicine and interact with each patient. – Participant, metropolitan health service [C/M] - Leggat, 2016<br><br>Consistent with the quantitative results, the interview participants reported that the participants had acquired skills in strategic thinking and planning, communication, project management, reflective practice and change management. [M] - Leggat, 2016<br><br>With respect to the second broader system outcomes, the interview participants reported that the participants had developed an organisational and systems literacy that enabled participants to understand the bigger picture and engage constructively with the imperatives contained within it. This enabled them to collaboratively influence outcomes, rather than avoiding or resisting involvement or giving up when encountering barriers. This was identified as a critical factor in both clinician engagement and promoting projects that had clear organisational impact. [M] - Leggat, 2016<br><br>Has helped me see how my work and leadership fits with strategic plan; that I am working to something bigger, how the health service works and how I can work in it. – Participant, rural health service [M] - Leggat, 2016<br><br>The curriculum was developed after conducting a review of the literature on the status of women in academic medicine and then tailored to the specific environment of the JHUSOM. [C] - Levine, 2015<br><br>We found a significant improvement in participant's self- rated skills across a majority of leadership domains following participation in a longitudinal, cohort-based, experiential leadership program for women. Our findings suggest that for

	our population, we correctly identified important areas for development and that the content was delivered in a format that enhanced perception of learning for participants. [C/M] - Levine, 2015<br><br>Participant evaluation of the CLP: learning, value and willingness to take on leadership roles (2012 cohort). Of the 20 participants, 14 (70 per cent) completed the CLP evaluation survey (Table III). Respondents reported the duration of individual sessions and the program as a whole to be "just right" and were overwhelming positive about the quality of information, the speakers and the value of the program. [C/O] - Macphail, 2014<br><br>The CLP significantly increased willingness to take on leadership roles. Most participants (93 per cent) reported that they were more willing to take on a leadership role within their team. Fewer were willing to lead at the level of department (79 per cent) or organization (64 per cent). Five of the 11 participants from the 2011 program had taken on a new leadership role 18 months later. [M/O] - Macphail, 2014<br><br>Participant follow-up (2011 program). Of the 11 participants who completed the pilot CLP in 2011, 9 (82 per cent) remain employed at the HCO 18 months after completing the program. Four had been promoted to more senior or managerial roles, including one who was redeployed to another section to assist in facilitating change to practice. Another member of the group has since participated within two separate quality improvement projects within the organization. [O] - Macphail, 2014<br><br>Also, creating the PMLP at Memorial University allowed the program to be tailored to meet the unique needs of physician leaders in NL. [C] - Maddalena, 2015<br><br>Between January and April 2011, a Provincial Needs Assessment was conducted to gauge interest, seek input on learning needs and provide guidance for program development. [C] - Maddalena, 2015<br><br>Subject matter experts were engaged to develop module content which addressed identified educational needs and presented information from the health authority and provincial perspectives. [C] - Maddalena, 2015<br><br>They report observing increased application of skills in areas such as conflict resolution, problem-solving (i.e. ability to solve more issues themselves) and quality care initiatives. They also report observing a broader understanding of the administrative system at the RHA level. [M] - Maddalena, 2015<br><br>The majority of participant survey respondents (95.07 per cent) reported feeling more prepared for their leadership responsibilities as a result of their participation in PMLP. [M] - Maddalena, 2015<br><br>Feedback about the learning activities delivered by QFI Consulting was, overall, very positive. Most of the interviewees found it useful, appropriate, context relevant and directly applicable to work. [C] - Miani, 2013<br><br>While generally positive about the program, participants identified several areas for its future development. For example, some participants commented that the leadership course was 'basic' and perhaps more appropriate for junior staff. This was a particular concern among more senior staff who had previously participated in leadership courses. [C] - Miani, 2013

	On a personal development level, almost all program participants who were interviewed reported feeling more empowered. They spoke about feeling 'braver', 'more daring', being 'better equipped' to achieve goals, and being 'more persistent': I have the tools with which to do the job, so that's empowerment. (Int7) [M] - Miani, 2013<br><br>Participants reported that the taught modules mostly contributed to developing their leadership in areas such as confidence, communication skills, negotiation skills and team management skills. [M] - Miani, 2013<br><br>The program was also seen to have contributed to improving the 'marketability' of a cohort of Fellows by enhancing their managerial skills and developing their abilities as clinicians. Interviewees noted that the Trust sought to capitalize on this achievement by offering positions to several of the external Fellows (two in scheme B, one in Scheme A, locum positions for two other Scheme A external Fellows under consideration; Int8). Other external Fellows were reported to be able to secure consultant level posts in other NHS trusts. It would therefore seem that the program has had a positive impact on the career of most of the external Fellows. One respondent noted that such an achievement could be seen to enhance the reputation of the Trust within the NHS. Career perspectives appeared more limited for internal participants as they were not systematically offered promotions or new roles within the Trust (Int8, 12), but it was reported that two internal Fellows (Scheme B) were promoted by the end of the Programme (Int8). [M/O] - Miani, 2013<br><br>Program based on adult learning theory. All classroom-based modules employ case-based, small group discussions and draw on Africa-focused examples to maximize relevance to the learners. [C] - Monroe-Wise, 2016<br><br>The clinical leaders program has assisted me to recognize and accept that some of my instinctual behaviors can be considered leadership qualities and it has provided me with the confidence to act upon them (Participant 2). [M] - Monroe-Wise, 2016<br><br>Twenty-one (68 %) of 31 responding alumni reported that their positions at work had changed since completing the fellowship. Of those who had experienced changes to their position at work, 16 (76 %) believed that the change was due to experience gained through participation in Afya Bora. Promotions or new jobs were the most common types (75 %) of career advancement mentioned. Others had taken on more responsibilities at work, and two had enrolled in higher academic degree programs. [O] - Monroe-Wise, 2016<br><br>Several program alumni commented on how their training during Afya Bora led to promotions in their workplaces. Specifically, they cited both skills learned during didactic modules and during experiential attachment site blocks, underscoring the importance of both modalities in adult learning theory. The leadership and management skills obtained from the modules as well as the project on PMTCT [Prevention of mother-to-child transmission] [that] I undertook during my attachment enabled me to be recognized to fill the position of PMTCT program manager, which was a promotion. [M/O**]** - Monroe-Wise, 2016<br><br>The Leadership Development Program was designed based on a comprehensive needs assessment conducted using prior interviews with faculty surgeons who were selected for participation in the program, described in greater detail elsewhere. Based on leadership skills and knowledge that surgeons indicated

	would benefit their real-world practice, the inaugural program curriculum was structured around 4 major domains: leadership, team building, business acumen, and health care context (Table I, with representative quotes of impact in these four domains). [C/M] - Pradarelli, 2016<br><br>Lastly, the program helped faculty surgeons become more knowledgeable regarding leadership concepts, such as business acumen and organizational structure. [M] - Pradarelli, 2016<br><br>Many participants described self-empowerment for leadership roles as a strength of the program. Faculty surgeons across levels of academic rank felt not only enabled but also capable of effecting change in their local environments. One participant reported, "I'm more confident about stepping up as a leader," demonstrating an empowered perspective. [M] - Pradarelli, 2016<br><br>Principles guiding curriculum development:<br>■ Building a QI project into the training program enhances learning and increases the likelihood of subsequent implementation.<br>■ On-site training can reach more employees than off-site training opportunities.<br>■ On-site training is more likely to be effectively integrated into local work environments, thus facilitating the success of QI projects. [C] - Rask, 2011<br><br>Participants initially correctly answered only 14.0 (46.7%) of the 30 knowledge questions. After completing the course, the average participant answered 24.5 (81.7%) of the questions correctly, reflecting an absolute improvement of 35.0 percent- age points. [M] - Rask, 2011<br><br>The RLA was an important vehicle to prepare and promote women for intra-departmental leadership progression, creating role models in leadership positions and thus enhancing the value and culture of the organization. [M/O] - Rask, 2011<br><br>Leadership skills and attributes. Comments about interpersonal and relationship attributes dominated in this group, in response to the request to list the most important attributes of a leader. Comments about interpersonal skills included "Listening" (43% of respondents); the ability to relate to people, "understanding others/all sides of a situation" (M); and "Recognize certain folks will not change and move on" (M). Once again, the highest percentages of participants agreed that the skills improved by LAM were ability to recognize weaknesses and strengths (91% and 82%; Tables 7 and Figure 3). Only 42% agreed that LAM improved their ability to take risks. Fifty- four percent of respondents indicated that their perspectives about leadership had changed as a result of LAM. Eleven people referred to acquiring a better understanding of leadership: "Realizing what it takes to be a leader" (M), "Gathered an appreciation for skills required for effective leadership" (M), and "I better understand the differences between leading and managing" (W). Ten participants commented about a change in their interpersonal relationships: "I have a greater appreciation for how difficult it is at home" (M). Fourteen percent said that their perspectives had not changed, and 1 individual said prior knowledge was reinforced: "LAM redefined things I had learnt in other settings" (M). [M] - Sanfey, 2011<br><br>Career advancement. Sixty-six percent of respondents (75% of men, 53% of women) were invited to apply for or offered positions post-LAM. Comments were grouped as follows: people who were already in leadership positions (11%); those who were looking for leadership opportunities in general (25%), "I

	am interested in expanding my role in some of the projects in which I am currently involved.” (W); and those with a specific position in mind (7%), for example, “division chief ” (W). Fifty-eight percent of women, compared with 19% of men, $\chi2(1) = 4.68$, $p < .05$, were actively seeking leadership roles (Table 5). [O] - Sanfey, 2011<br><br>Participants experienced a transformational shift in how they constructed their identity as a physicians and leaders, and questioned assumptions about the physician’s role in healthcare. [M] - Smith, 2014<br><br>While there were a couple of physicians attending the FLA for a specific purpose of enhancing their current performance and positions and are not considering advancing into greater leadership roles, most of the group was interested in developing their leadership acumen further. - Smith, 2014<br><br>End of course evaluations for the eight content sessions yielded averages of 4.3 or higher on a five-point agreement scale across ten questions, with five of ten questions receiving a score of 5 for all sessions. Participants rated facilitators, content, location, meals, and applicability of content to their job. [C] - Throgmorton, 2015<br><br>I found it to be an overwhelming positive experience that was helpful to me. The only downside was all the information and trying [...] to remember it all and bring it into the way that you do things every day. [C] - Throgmorton, 2015<br><br>I came in with a minimal foundation of leadership and I left with tools [...] I took something away from every session [...] There are so many great things. I could give you a take home message on each session. The PLA made it clear that there are so many resources for us [...] Teaching us that foundation [...] I can’t even put into words how helpful it is. [M] - Throgmorton, 2015<br><br>There were some feelings expressed by more experienced physicians that much of the PLA content was not new to them. One stated: I have been in leadership positions for a lot of years. So I think over the years I developed a pretty good level of understanding of my style and so forth. Like when we did the DiSC, I thought it was interesting, but it did not tell me something I was not already aware of [...].[C] - Throgmorton, 2015<br><br>End of session responses to the survey question “I learned new knowledge and skills during this developmental session” yielded an average 4.5 response on a five-point agreement scale. [M] - Throgmorton, 2015<br><br>I do think the PLA has giving me a little bit of confidence to understand that while I may not be the most experienced one in the room that I have got something to offer. [M] - Throgmorton, 2015<br><br>Self-awareness gave me more confidence [...] I am much more reserved and it allows me to put myself out there just a little bit more. [M] - Throgmorton, 2015<br><br>Stakeholders targeted increased committee participation as an organization-impact result. This PLA cohort all served on a committee prior to the start of the PLA. [O] - Throgmorton, 2015<br><br>Practical application of acquired knowledge within interactive team- based learning during the residential components was mostly valued. [C] - Toma, 2020<br><br>SQSF was successful at consistently increasing awareness, knowledge and skills

	for most participants. Between 89%–96% of survey respondents indicated very strong agreement with statements regarding impacts on capability and confidence to practice (table 3). [M] - Toma, 2020<br><br>My skills around using QI methodology have massively improved. I've moved from being quite narrowly focused on my specific clinical area to having much broader horizons, and having the knowledge, skills and confidence to work on bigger service developments in areas that have been notoriously unimproved and unimprovable. (I14, Nurse) [M] - Toma, 2020<br><br>Several participants described how taking part in SQSF reinvigorated their career through attaining a more senior position with a designated QI national or organizational role, which often involved a change from 'doing' to 'leading' others: I am now able to influence more system wide change and will continue to inspire a network of improvers and innovators who have knowledge, skills and contacts to lead change and drive QI agenda in Scotland and beyond. (I07, Hospital Consultant) [M/O] - Toma, 2020<br><br>A minority of fellows have established or scaled national or international projects. Some examples include becoming active partners in strategic international collaborations with the aim of improving health and social care, at a significant scale and pace: I became a National Clinical Lead shortly after I finished my Fellowship. I am now advising a wide range of international partners in development of QI strategies, including Brazil, Canada and Australia. Allegedly I am now viewed as a QI expert and I feel like I am the 'go to' person for QI advice. (I16, Hospital Consultant). [M/O] - Toma, 2020<br><br>"Leaders Growing Leaders" became the name of our leadership development program comprised of 4 tiers (Table 1). For the cohort- based faculty learning communities in Tiers II and III, the leadership team (department chair, associate chair, and vice chair) carefully selected potential faculty participants and requested that they submit a brief declaration of their interest and commitment in the program. [C] - Torbeck, 2018<br><br>The decision to then become "selective" and form cohorts for Tiers II-IV is a strategy that we plan to continue because it allowed us to work on the "transfer of knowledge" in a more focused manner with people who we thought and hoped were most ready to engage. [C] - Torbeck, 2018<br><br>Reaction (satisfaction data)—Feedback from each post- session survey of our surgeons as leaders Tier I workshops indicated that respondents' overall reaction to the workshop was positive to extremely positive. Most respondents indicated that they were able to think about their current leadership style as it applied to the topic and planned to use some of the knowledge and skills learned in the future. Both the Tier II and III Leadership post series surveys showed all respondents valuing the topics as well as indicating that the timing was right for them in their careers. [C/M**]** - Torbeck, 2018<br><br>In terms of the Results (impact) outcome level, 2 out of the 3 faculty who recently interviewed for a vacant education leadership position in the department were participants of Tier II. Additionally, in the last 5 years, 5 of the 6 division chiefs have turned over and all have been replaced by internal candidates whom we have groomed through various leadership skill building programs. [O] - Torbeck, 2018<br><br>The UCSF-Coro FLC is a program administered within UCSF by Coro. Coro experiential training model was developed in the 1940s. The training model

	shares similar concepts of Kolb's experiential learning model [15], which was refined from Dewey's educational principles emphasizing concepts such as experience, experiment, observation and reflection, and purposeful learning [16]. Coro's design of curriculum and learning activities encourages participants to accept new tools introduced in the training, apply the tools, and to then adapt the tools, as needed, based on one's experiences and relevancy after a process of reflection. [C] - Tsoh, 2019<br><br>At an individual level, the program fostered development of leadership skills in conflict resolution, team management, and giving and receiving feedback. Since completion of the FLC, a majority indicated noticeable changes in leadership skills (98.6%), and attitudes or behaviors related to leadership (91.7%). [M] - Tsoh, 2019<br><br>A majority (91.7%) said the program had increased their understanding of UCSF as an organization; 79.2% credited the FLC to an increased ability to navigate UCSF as an organization. [M] - Tsoh, 2019<br><br>Specifically, the most commonly endorsed individual changes include a positive effect on ability to lead or manage through challenging times (93.1%), increased confidence in handling leadership roles or responsibilities (80.6%), and increased effectiveness in leadership roles (77.8%). [M] - Tsoh, 2019<br><br>As of 2017, 9.6% (n = 13) of the graduates attained leadership position at UCSF, defined as Dean (including Vice and Associate Dean positions), Department Chair, and Director of Organized Research Unit. [O] - Tsoh, 2019<br><br>Post completion of the FLC, while only 29% indicated volunteering more for committee service, 62.5% reported seeking out new leadership opportunities, and 61.1% were appointed to one or more committees as an outgrowth of the program. More women than men agreed their program participation encouraged them to expand their leadership roles in professional or volunteer organizations outside of UCSF (72.7% vs. 47.8%; $\chi2(1) = 4.1$, $p = 0.04$)." [O] - Tsoh, 2019<br><br>Two-thirds (66.2%) perceived a positive impact on recruiting or retaining faculty or agreed that participating in FLC increased their commitment to UCSF. [O] - Tsoh, 2019<br><br>The content of the program was based on a needs assessment conducted with faculty surgeons who were selected for participation. A full description of methods can be found in Jaffe et al. [C] - Vitous, 2019<br><br>When asked to reflect on how the LDP affected leadership style, many participants perceived that their approaches have become more collaborative and cited personal growth in a variety of areas, including the following: paying greater attention to strengths and weaknesses of colleagues, improving listening skills, decreasing agenda setting, and improving the ability to delegate. This transition has contributed to a more participatory approach to how decisions are made in the Department of Surgery at the University of Michigan. [M] - Vitous, 2019
<u>Valuing physician leaders and organizational commitment (CMO5)</u><br><br>If LDPs reflect that hospitals value physician leaders by facilitating program participation and taking the program seriously [C], physicians feel appreciated, commit to the organization, and are more willing to adopt new leadership roles [M]. This strengthens the leadership pipeline [O], which seems especially true for	

underrepresented groups in the organization's leadership pipeline [C].	
Studies that provide (partial) evidence	DeRusso, 2020; Ennis-Cole, 2019; Fassiotto, 2018; Hopkins, 2018; Levine, 2015; Macphail, 2014; Miani, 2013; Sanfey, 2011; Smith, 2019; Throgmorton, 2015; Tsoh, 2019.
Illustrative supporting evidence (text fragments, descriptions, data)	Participants felt honored to be selected for the program and indicated that protected time, sessions with senior physicians, training by experts in the business community, and the inclusion of only physicians were critical elements of the program. [C/M] - DeRusso, 2020<br><br>Survey results indicated that all 125 participants from the 4 cohorts agreed their leadership skills were enhanced, they felt more connected to the institution, and they were committed to contributing to the enterprise-wide mission. [M] - DeRusso, 2020<br><br>Across interviews, the participants stated that they felt their involvement in the leadership development program was an investment by the Center in their personal development and growth. They perceived that the investment meant that the Center believed in them. The participants agreed that their engagement was positively impacted by this perception. This is important to physicians because they do not like to stay in the wrong or be unappreciated. Cullum (2016) indicated that physician P08 commented: I'm going to stick around to see what it is like for people to invest in me other than clinical productivity and revenue. I'm staying as long as I'm feeling that investment. I'm going to be very loyal and very engaged and I have been because there is a continuous investment. [C/M/O] - Ennis-Cole, 2019<br><br>Following the leadership development program, nine out of the 10 participants graduated from the program and moved into new or enhanced leadership positions. Participants reported that their capacity for collaboration increased and their new leadership skills were utilized in their new leadership roles. [O] - Ennis-Cole, 2019<br><br>Three items from 'Attitudes: perceptions of institutional support' were significantly higher than non SLP participants. [M] - Fassiotto, 2018<br><br>I thought it was an incredible gift. It gave me basic tools I didn't have before to be a leader within every team I work on. It helped me to understand myself better too (Female, Assistant Professor at time of SLDP participation). [M] - Fassiotto, 2018<br><br>Given the literature on effects of the "bamboo ceiling" in career advancement for Asian professionals and the dearth of Asian healthcare leaders in academic medicine, this result is meaningful as it provides one potential mechanism, inclusive leadership training, by which Asian health professionals may advance in their careers (Hyun, 2006; AAMC, 2017). Additionally, our data revealed that women program participants had significantly higher odds than their non-participant counterparts of remaining at the institution. This finding highlights the importance of perceived organizational support for retention and its potentially greater impact on women's turnover intentions (Eisenberger et al., 2002; Jawahar and Hemmasi, 2006; Rhoads and Eisenberger, 2002). [C/O] - Fassiotto, 2018<br><br>Invitations to participate in the program were highly selective. [C] - Hopkins, 2018

	Learning: Significant improvements, were reported in knowledge, skills, and attitudes surrounding leadership competencies. Including: connected to the institution [M] - Hopkins, 2018<br><br>The specific goals of the LPWF are to develop Johns Hopkins University School of Medicine women leaders who will contribute to future initiatives throughout the school, retain emerging female leaders by providing a prestigious and challenging learning experience that may lead to new opportunities and promotion, and advance the school of medicine's core values of diversity and inclusion. [C] - Levine, 2015<br><br>Finally, women may be more likely to value themselves as leaders because the institution, through support of the LPWF, demonstrates that it values women leaders.[Discussion]. [C/M] - Levine, 2015<br><br>Executive and line manager support was believed to contribute to achieving the feasibility of the program. This imbued the program. with a greater sense of credibility and demonstrated that the CLP and its participants were valued by the HCO. [C/M] - Macphail, 2014<br><br>The CLP significantly increased willingness to take on leadership roles. Most participants (93 per cent) reported that they were more willing to take on a leadership role within their team. Fewer were willing to lead at the level of department (79 per cent) or organization (64 per cent). Five of the 11 participants from the 2011 program had taken on a new leadership role 18 months later. [M/O] - Macphail, 2014<br><br>Participant follow-up (2011 program). Of the 11 participants who completed the pilot CLP in 2011, 9 (82 per cent) remain employed at the HCO 18 months after completing the program. Four had been promoted to more senior or managerial roles, including one who was redeployed to another section to assist in facilitating change to practice. Another member of the group has since participated within two separate quality improvement projects within the organization. [O] - Macphail, 2014<br><br>There appeared to be consensus among programme participants that the level of support provided by senior management in the Trust was instrumental in the development and achievements of Programme goals. Interviews in particular highlighted the role of the chief executive officer (CEO) whose support was reported to be perceived as strong and visible, demonstrated in her attending some of the training sessions (Int12) and adopting an 'open door policy' for external Fellows (Int10). Other members of senior management were also acknowledged to provide important support. [C] - Miani, 2013<br><br>The program was also seen to have contributed to improving the 'marketability' of a cohort of Fellows by enhancing their managerial skills and developing their abilities as clinicians. Interviewees noted that the Trust sought to capitalize on this achievement by offering positions to several of the external Fellows (two in scheme B, one in Scheme A, locum positions for two other Scheme A external Fellows under consideration; Int8). Other external Fellows were reported to be able to secure consultant level posts in other NHS trusts. It would therefore seem that the program has had a positive impact on the career of most of the external Fellows. [O] - Miani, 2013<br><br>The percentage of women participating in LAM has been steadily increasing, and the age of women participants has been decreasing. This reflects the changing institutional policy to target women and emerging or potential leaders, instead

	of more-established leaders as in the earlier years. [C/O] - Sanfey, 2011<br><br>Seventy-four of the 110 (67%) participants were male. This is the opposite of the more recent 2008 cohort, which reflected an increased institutional effort to target emerging women leaders. [O] - Sanfey, 2011<br><br>Although minimal gains have been demonstrated nationally over the past decade, during the period described in this article, our department faculty gender composition went from 27% to 39% women. Of unique importance, dramatic changes in the makeup of faculty leadership positions of vice chairs, chiefs of service, and division directors were seen: from 6% at the inception of the RLA program to the 32% current representation of women faculty leaders. The RLA was an important vehicle to prepare and promote women for intra-departmental leadership progression, creating role models in leadership positions and thus enhancing the value and culture of the organization [C/O] - Smith, 2019<br><br>All those interviewed shared something of value to them personally and professionally. I thought it was a good program. I was very honored and flattered to be asked [...].[C/M] - Throgmorton, 2015<br><br>Of the 16 interviewed, 13 reported an enhanced connection to (organization)'s strategic plan (1 reported a strong connection already and 1 a minor connection). [M] - Throgmorton, 2015<br><br>Stakeholders targeted increased committee participation as an organization-impact result. This PLA cohort all served on a committee prior to the start of the PLA. [O] - Throgmorton, 2015<br><br>Most respondents (91.7%) agreed that the sponsorship of the FLC demonstrated the University's commitment to foster faculty development. [C] - Tsoh, 2019<br><br>'It is an important symbol to me, personally, of the university's interest in "growing its own" and, in particular, of this public institution's continued interest in trying to assure that its leaders look like the public the university serves.' [C/M] - Tsoh, 2019<br><br>When selecting applicants for admission to the program, the committee made a conscious effort to address issues of equity and inclusion (e.g., by gender, URM status, school, and department). [C] - Tsoh, 2019<br><br>'I feel more engaged with the university as a whole, and more interested in collaborating with others outside of my department.' [M] - Tsoh, 2019<br><br>As of 2017, 9.6% (n = 13) of the graduates attained leadership position at UCSF, defined as Dean (including Vice and Associate Dean positions), Department Chair, and Director of Organized Research Unit. [O] - Tsoh, 2019<br><br>Post completion of the FLC, while only 29% indicated volunteering more for committee service, 62.5% reported seeking out new leadership opportunities, and 61.1% were appointed to one or more committees as an outgrowth of the program. More women than men agreed their program participation encouraged them to expand their leadership roles in professional or volunteer organizations outside of UCSF (72.7% vs. 47.8%; $\chi2(1) = 4.1$, $p = 0.04$)." [O] - Tsoh, 2019<br><br>Two-thirds (66.2%) perceived a positive impact on recruiting or retaining faculty

	or agreed that participating in FLC increased their commitment to UCSF. [O] - Tsoh, 2019
**The interconnectedness of organizational culture, quality improvement, leadership pipeline**	
Studies that provide (partial) evidence	Berghout, 2020, Lewis, 2021, Miani, 2013, Rask, 2011, Smith, 2014<br>Smith, 2019
Illustrative supporting evidence (text fragments, descriptions, data)	It was not always a smooth transition when physicians returned to their own hospital. Physicians particularly experienced a lack of support from peers and hospital administrators with regard to their project and personal developments. In addition, they were not always granted the extensive time required for executing improvement projects because the daily pull of clinical work was perceived as too strong. This arguably hindered some physicians from wholeheartedly embracing their preferred identity as collaborative leader as the following quote illustrates: I experienced difficulties in finding my role. You're not a medical manager, you're not part of the (hospital/medical) board. So what's your role then? But there's expected a lot from you. You receive no formal support or feedback while you do need that. (Respondent 10, z in-house session 18 June 2018) [O culture - O leadership] - Berghout, 2020<br><br>Successful IAP implementation helped fellows enhance their visibility and reputation. Projects that aligned with institutional priorities enhanced the reputation of both the institution and fellow, either directly through project outcomes, or indirectly through development of the fellow as a future leader. When projects enhanced institutional missions and organizational culture, fellows had new leadership opportunities and their IAPs were more likely to achieve intended goals. [O culture – O quality - O leadership] **-** Lewis, 2021<br><br>This finding was supported in interviews, with mentions of opportunities to work better as a team (Int7) and to improve people management considered a strength of the Program: The team work, the group work, I mean talking about different people and how we impact, how we think about making changes for the benefit of the patient. All this is happening as well and I think this is a good change and if we keep doing that it will make a huge difference in the communication and eventually it will make a great difference to the patient. (Int15) [O culture - O quality] - Miani, 2013<br><br>The two-pronged approach was designed to reach multiple levels of the organization to more rapidly achieve culture change and promote QI adoption. The courses were initially offered four times a year to build a critical mass of trained employees and are now offered twice a year (in the spring and the fall), with a cohort of approximately 30 in each class to reach new employees and employees in clinical areas of strategic importance to the organization. [O culture - O leadership] - Rask, 2011<br><br>leadership positions of vice chairs, chiefs of service, and division directors were seen: from 6% at the inception of the RLA program to the 32% current representation of women faculty leaders. The RLA was an important vehicle to prepare and promote women for intra- departmental leadership progression, creating role models in leadership positions and thus enhancing the value and culture of the organization. [O culture - O leadership] - Smith, 2019<br><br>Projects often expanded into a sustainable process or program with positive business or cultural impact. Highlighted next are four such projects. [O culture - O quality] - Smith, 2019<br><br>Some physicians struggled with the decision to pursue leadership and the

	personal mental cost associated both internally and externally with their peers. This is a cultural barrier that is not easily traversed. In the medical culture, physicians who choose to move to a leadership position as opposed to continuing to practice in a clinical capacity are often negatively viewed. Mentoring would be an important addition for these physicians with physician leaders both inside the organization as well as outside of the region. [O culture - O leadership] - Smith, 2014
**Leadership ecosystems**	
Studies that provide (partial) evidence	Berghout, 2020, Bhalla, 2018, Christensen, 2016, Daniels, 2014, DeRusso, 2020, Fernandez, 2016, Hopkins, 2018, Howell, 2019, Macphail, 2014, Miani, 2013, O'Neil, 2019, Pradarelli, 2016, Rao, 2017, Sanfey, 2011, Shah, 2013, Smith, 2014, Smith, 2019, Steele, 2020, Toma, 2020, Torbeck, 2018, Tsoh, 2019
Illustrative supporting evidence (text fragments, descriptions, data)	Although the construction of the collaborative self was a key development in the MLDP trajectory, it was not always a smooth transition when physicians returned to their own hospital. Physicians particularly experienced a lack of support from peers and hospital administrators with regard to their project and personal developments. In addition, they were not always granted the extensive time required for executing improvement projects because the daily pull of clinical work was perceived as too strong. This arguably hindered some physicians from wholeheartedly embracing their preferred identity as collaborative leader as the following quote illustrates: I experienced difficulties in finding my role. You’re not a medical manager, you’re not part of the (hospital/medical) board. So what’s your role then? But there’s expected a lot from you. You receive no formal support or feedback while you do need that. (Respondent 10, z in-house session 18 June 2018). - Berghout, 2020<br><br>A lack of support by others led to identity violations as this obstructed some participants to be their preferred collaborative self. These identity violations caused participants stress and work dissatisfaction and hindered some participants from fully realizing their collaborative ambitions. - Berghout, 2020<br><br>However, from its inception, the CQFP curriculum has evolved each year based on changes in health care delivery models, health policy and payer priorities, and shifts in consumer demand. The passage of the Affordable Care Act (ACA) and the state health policy environment have had a significant impact on the curriculum, which now includes performance-based programs developed as part of the ACA and within the region. Refinements to the curriculum also are made annually based on feedback from the fellows and faculty. - Bhalla, 2018<br><br>The CQFP program actively continues. In addition to curriculum evolution related to new federal and state policy initiatives, regional faculty composition is being expanded. It is anticipated that interest in the program will continue to grow, as it has been associated with career progression. CQFP fellow alumni have expressed interest in becoming faculty or mentors for future classes. An annual “culminating dinner” for each class brings together graduating and prior fellows. A network of fellows is developing, all of whom receive UHF and GNYHA mailings on issues of health care policy and quality, and for whom a more formal social media networking vehicle is being evaluated. GNYHA and UHF also are exploring the option of connecting the CQFP with an academic institution to allow fellows interested in obtaining advanced degrees to receive credits for completing the CQFP. - Bhalla, 2018<br><br>Attendees are aided by executive coaches, connect with the broader network of course alumni through an electronic portal and network, and prepare a brief

	address framing their vision for their colleagues as a capstone experience of the course. To date, 114 individuals from 21 countries (including Australia) have attended the Samson Global Leadership Academy. - Christensen, 2016<br><br>At this time, Afya Bora includes web resources and professional networking for participants intended to foster further development and maintenance of positive health leadership practices. - Daniels, 2014<br><br>Our analysis showed limited evidence that participants reached the maintenance stage of the health leadership model. It is possible that the evaluation activities at three months post-fellowship did not allow fellows enough time to reach this stage. Alternatively, it may suggest that the ability to effect sustainable organizational change may require expanding the post-training interventions to support. - Daniels, 2014<br><br>The program has evolved in response to feedback and experiences from year to year. - DeRusso, 2020<br><br>It is important to note that the cohort surveyed completed the course prior to the initiation of a structured post-course skills support system, which was incorporated into the curriculum with the 2014 cohort. Thus, the Fellows in the analysis received no such ongoing learning supports made available to them from the course. - Fernandez, 2016<br><br>Minor adjustments were made to the curriculum and presenters over the 4 years of the program based on participant feedback. - Hopkins, 2018<br><br>Even though there were significant improvements at the end of the program in perceptions of institutional support and connectedness, participants still had concern about support for career development and general hospital support. In addition to a training program, organizations wanting to develop physician leaders need to look at how this role will be supported. - Hopkins, 2018<br><br>Pathology Leadership Academy will use participant and chair feedback for ongoing curricular development to ensure topics continue to address major needs of academic pathology. - Howell, 2019<br><br>Based on these considerations, it was considered practicable and valuable to continue the program on an ongoing basis. The HCO plans to expand the program to incorporate additional staff from other clinical departments (e.g. staff employed in nursing homes). The results of participant surveys in 2013 and 2014 will be considered by the executive to determine the need for a more comprehensive evaluation. Follow-up in five years of these two cohorts will be of interest to determine if there is any enduring effect. - Macphail, 2014<br><br>At the same time, there appeared to be a trend for negative attitudes from staff outside the program to have fallen over time, with the number of respondents reporting not having experienced disapproval from others increasing from under 20 percent to almost 40 percent as the program evolved. Reported reasons for lack of support and resistance towards program participants included a perceived general resistance to change and skepticism towards new ideas that some considered to be inherent in clinicians' culture. - Miani, 2013<br><br>The success of the APLA has generated a reinvigorated sense of organizational connection and physician engagement, and sparked further development of physician-led initiatives including the development of future cohorts of physician leaders at the director level as well as high potentials. In fact, twenty-

	six emerging physician leaders joined together from across the organization to kick off a second cohort of the APLA in January 2017. Several of the APLA participants from the first cohort served as AL sponsors to provide guidance and mentorship throughout the process. THS has only just begun their journey of developing physician leaders as change agents, but the results of the APLA program suggest great promise for positioning the organization for success in the future of value-based care delivery. - O'Neil, 2019<br><br>Finally, one of the most important aspects in designing a leadership development program is to ensure organizational systems are in place for graduates to continually apply their learnings and that there are opportunities for leadership growth in the form of promotion and career trajectory. Otherwise, future leadership development initiatives are met with apathy and skepticism – creating a sort of why bother if nothing comes out of it? The APLA participants in this program demonstrated a range of continued involvement in working together on their projects once the formal AL component was completed. Teams with sponsors who kept the momentum going tended to continue working on their projects; whereas teams without a 'push' from their sponsors, tended to lose steam on their projects. As such, we learned that designing a structured process for AL teams to continue project work post-graduation is crucial to sustaining the learning and momentum of the program. - O'Neil, 2019<br><br>The second iteration of the Leadership Development Program was changed in several ways based on feedback from the inaugural program described here. These immediate changes focused mainly on reformatting the curriculum to enhance the integration and delivery of information. Speakers who were thought to be less effective based on participants' responses were replaced. - Pradarelli, 2016<br><br>Previous CPIP graduates serve as coaches for current attendees, which helps broaden the learning resources for new students and reinforce previous training for coaches. - Rao, 2017<br><br>The two-pronged approach was designed to reach multiple levels of the organization to more rapidly achieve culture change and promote QI adoption. The courses were initially offered four times a year to build a critical mass of trained employees and are now offered twice a year (in the spring and the fall), with a cohort of approximately 30 in each class to reach new employees and employees in clinical areas of strategic importance to the organization. - Rao, 2017<br><br>Finally, there are curricular gaps in the related themes of sustainability of performance improvement and spread of interventions. When the course ends, participants typically have insufficient data to discern statistical signals that their interventions are having an impact; addressing sustainability at that time is somewhat premature. However, extending the duration of the course would require more time of the physicians. To address this, the research team is piloting a program in which select teams are supported for a postgraduate period during which they are exposed to a curriculum focused on these topics and continue to work on their project with faculty coaching. - Rao, 2017<br><br>The LAM faculty made a number of modifications to the program content and format in response to institutional needs and participant feedback. - Sanfey,2011<br><br>The comments by the long-term Post-LAM participants emphasize the ongoing

	struggle faced by people who have the ability to lead but are not nurtured: “Not at all successful due to lack of support, still just putting out fires” and “Still have difficulty saying No to things and getting overloaded.” - Sanfey, 2011<br><br>Although the benefits of LAM participation are sustained for some years, there is a need for reinforcement to prevent skill attrition. - Sanfey, 2011<br><br>The researcher suggests the course co-directors identify opportunities to be the bridge for successful clinicians engaging in leadership while still maintaining their identities as physicians. Some physicians struggled with the decision to pursue leadership and the personal mental cost associated both internally and externally with their peers. This is a cultural barrier that is not easily traversed. In the medical culture, physicians who choose to move to a leadership position as opposed to continuing to practice in a clinical capacity are often negatively viewed. Mentoring would be an important addition for these physicians with physician leaders both inside the organization as well as outside of the region. - Smith, 2014<br><br>Notably, 60% of RLA alumni continued their involvement with the program in one or more ways, such as serving as a presenter or panelist, team project coach, program planning contributor, or mentor to RLA participants. - Smith, 2019<br><br>Projects often expanded into a sustainable process or program with positive business or cultural impact. Highlighted next are four such projects. - Smith, 2019<br><br>A continuous improvement culture allowed the program to keep up with new challenges in the environment and changing organizational needs. - Smith, 2019<br><br>Over time, changes to the program have been implemented based on informal interviews with fellows. For example, leadership project guidelines were revised in 2018 such that the project needs to be beneficial to the fellow’s home institution, address a fellow’s self-identified leadership gaps, and be submitted to the COD Administrative Board for review (List 1 provides examples). Other changes that have been implemented include increasing the number of dean mentor shadowing opportunities from 1 to 2 (2013); providing recommended reading on leadership (2017); and providing information about the executive search, recruitment, and interview processes (2017). - Steele, 2020<br><br>Other fellows described how they contributed to establishing QI infrastructures such as QI academies that drive further related capability and capacity opportunities. - Toma, 2020<br><br>Key strengths included a strong historical commitment from the SQSF leadership and support team to routinely collect and collate good quality programme and evaluation data, which was made available for this study. - Toma, 2020<br><br>Third, our evidence suggests that despite overwhelmingly positive reactions and learning, similar to other comparable QI educational programmes,7 11 the extent to which participants were able to transfer training into the workplace was mediated by a wide range of interrelated situational factors such as internal motivations, supportive organizational culture, available resources and further coaching and feedback on an ongoing basis. - Toma, 2020<br><br>Consolidating post- fellowship mechanisms can also be useful so that participants can continue to network and collaborate with peers and colleagues

	about successes or challenges in creating and sustaining improvements. - Toma, 2020<br><br>Interestingly, several Tier III faculty were sorry to see the leadership series end and most indicated that they would like to continue on in some fashion with their leadership cohort. To that end, each Tier III faculty receives a “Weekly Leadership Pearl (WLP)” in the form of an e-mail. - Torbeck,2018<br><br>While subsequent iterations continue to evolve both content and structure, more can be done to sustain these multi-level impacts. Strategies might include (1) additional support beyond the program to maintain cross-institutional connections and peer support, (2) reinforcement and further skill-building (e.g., booster sessions to bring graduated cohorts together), and (3) support for graduates in accelerating transitions to new leadership positions (e.g., ‘follow-on experiences’ to sustain or reinforce new skills and move the graduates more swiftly towards efficacy in various leadership roles). - Tsoh, 2019<br><br>This article describes perspectives from our 12-year experience cultivating a formal faculty LDP within an academic health center and<br>longitudinal outcomes of our LDP.<br>More Full Professors agreed to the statement that the program improved the climate for UCSF than Associate or Assistant Professors (78.8% vs. 45.0%; $\chi2(1) = 7.8$, $p = 0.005$).). - Tsoh, 2019
